# The multicellularity genes of dictyostelid social amoebas

**DOI:** 10.1038/ncomms12085

**Published:** 2016-06-30

**Authors:** Gernot Glöckner, Hajara M. Lawal, Marius Felder, Reema Singh, Gail Singer, Cornelis J. Weijer, Pauline Schaap

**Affiliations:** 1 Institute of Biochemistry I, Medical Faculty, University of Cologne, Cologne D-50931, Germany; 2 Leibniz Institute of Freshwater Ecology and Inland Fisheries (IGB), Müggelseedamm 301, Berlin D-12587, Germany; 3 Division of Cell and Developmental Biology, School of Life Sciences, University of Dundee, Dundee DD15EH, UK; 4 Leibniz Institute on Aging, Beutenbergstr.11, Jena D-07745, Germany

## Abstract

The evolution of multicellularity enabled specialization of cells, but required novel signalling mechanisms for regulating cell differentiation. Early multicellular organisms are mostly extinct and the origins of these mechanisms are unknown. Here using comparative genome and transcriptome analysis across eight uni- and multicellular amoebozoan genomes, we find that 80% of proteins essential for the development of multicellular Dictyostelia are already present in their unicellular relatives. This set is enriched in cytosolic and nuclear proteins, and protein kinases. The remaining 20%, unique to Dictyostelia, mostly consists of extracellularly exposed and secreted proteins, with roles in sensing and recognition, while several genes for synthesis of signals that induce cell-type specialization were acquired by lateral gene transfer. Across Dictyostelia, changes in gene expression correspond more strongly with phenotypic innovation than changes in protein functional domains. We conclude that the transition to multicellularity required novel signals and sensors rather than novel signal processing mechanisms.

## Abstract (web summary)

Unicellular social amoebae aggregate to form a multicellular life stage, making them a model system for the evolution of multicellularity. Here, Glöckner *et al.* use a comparative genomic and transcriptomic approach to determine the origin of the genes essential for multicellularity in the social amoebae.

## Graphical abstract

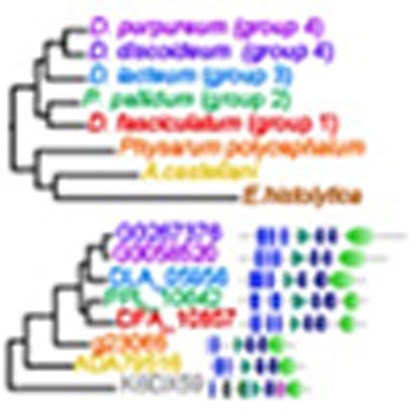

Multicellularity provided the opportunity for cell-type specialization and the positioning of specialized cells into complex structures. This organization offered an immense gain of function that is reflected in the complex physiology and behaviour of modern animals such as ourselves. To understand how multicellular organisms evolved, it is essential to retrace the origin of the cell–cell communication mechanisms that caused cell-type specialization. While the early ancestors of animals are long extinct, several multicellular eukaryotes still have a unicellular feeding stage, simplifying identification of genes that uniquely regulate multicellular development. The best-studied representative of this group is *Dictyostelium discoideum (DD)*, which forms multicellular structures by aggregation of starving amoebas, which then differentiate into four distinct cell types^1^. Dictyostelia are members of the mostly unicellular kingdom of Amoebozoa and can be subdivided into two major branches I and II, each containing two major groups^2^,^3^. Phenotypic mapping shows that these groups differ in the number of different cell types, the size and shape of multicellular structures, the presence of a freely migrating ‘slug' stage and light-orientated behaviour. The largest changes occurred at the transition between groups 3 and 4, with group 4 acquiring two novel cell types, cell-type proportioning, the ability to form larger multicellular structures and greater behavioural complexity^3^,^4^.

To understand the genetic changes that caused the evolution of multicellularity and the acquisition of multicellular complexity, we completed the set of genomes representing groups 1, 2 and 4 of Dictyostelia^5^,^6^,^7^ with the genome of the group 3 species *D. lacteum* (*DL*). The group 1–3 genomes were sequenced to deep coverage. Almost complete assembly was achieved by primer walking, and assisted by detailed fosmid maps of the genomes. In addition to the manually curated *DD* genome, these high-quality genomes allow accurate assessment of gene gain, modification and loss in the course of Dictyostelid evolution. We used existing^8^ and novel high-throughput RNA sequencing data to determine differences in gene expression between groups during the entire developmental programme. The genomes of three unicellular amoebozoans are also available: the obligatory parasite *Entamoeba histolytica*^9^ and the free-living amoebozoa *Acanthamoeba castellani*^10^ and *Physarum polycephalum*^11^. The availability of multiple uni- and multicellular amoebozoan genomes enabled us to also assess genetic changes at the transition from uni- to multicellularity.

In the model *DD*, ∼385 genes have been recognized by gene disruption to be essential for normal progression through multicellular development. We investigated the presence of these genes across five Dictyostelid and three unicellular Amoebozoan genomes, and identified their closest homologue outside of Amoebozoa. We next assessed changes in gene function by comparing the functional domain architecture of orthologous genes across species and we assessed changes in gene regulation by comparing their transcriptional profiles. We performed gene ontology (GO) enrichment analysis to assess which functional categories of genes were most subjected to change at either the transition from uni- to multicellularity or during the acquisition of multicellular complexity.

Our analysis shows that out of the 385 investigated genes, 305 are conserved throughout Dictyostelia and at least one unicellular amoebozoan. This set is enriched in cytosolic, nuclear and cell cortex proteins, and in protein kinases. The remaining 80 developmentally essential genes (DEGs), unique to Dictyostelia, mostly encode cell surface and secreted proteins, with roles in sensing and cell recognition. These proteins belong to expanded families that show extensive gene gain and loss between individual Dictyostelia and unicellular Amoebozoa. Three enzymes that synthesize signals that induce cell differentiation were acquired from bacteria by lateral gene transfer (LGT). In short, most mechanisms required for signal processing were already present in the unicellular ancestors, while the transition to multicellularity mainly required novel secreted and exposed signals and sensors to detect these signals.

## Results

### *DL* genome sequencing

The *DL* genome was sequenced to 29-fold coverage using the 454 Roche platform. Assembly was assisted by a detailed map of end-sequenced fosmids with average insert sizes of 32.5 kb. Remaining gaps in the scaffolds were closed by primer walking to yield a final assembly of 54 gap-free contigs. The *DL* genome was at 23.4 Mbp ∼30% smaller than the *DD* (group 4), *D. purpureum* (*DP*, group 4) *Polysphondylium pallidum* (*PP*, group 2) and *D. fasciculatum* (*DF*, group 1) genomes, which was due to fewer and smaller introns, smaller intergenic regions and fewer protein-coding genes (Supplementary Table 1). *DD* has unusual chromosome ends consisting of A/T-rich repeats, as are also present at the extrachromosomal ribosomal DNA palindrome ends^5^. Both the *DF* and *PP* chromosomes and palindromes have normal eukaryote GT-rich telomere repeats, while *DL* presents an intermediate situation with normal eukaryote chromosome telomeres and *DD-*like A/T-rich palindrome ends (Supplementary Fig. 1).

### Comparative analysis of DEGs

DEGs are defined as *DD* genes for which deletion causes defects in multicellular development. Around 385 DEGs were retrieved from Dictybase^12^. The DEGs were subdivided in sets, according to the developmental stage where the defect was first evident: growth, aggregation, mound/tip formation, slug formation and migration, culmination, pre(stalk) and pre(spore) differentiation. The genes are listed in Supplementary Data 1 together with their null mutant phenotype, their orthologues/homologues in *DP*, *DL*, *PP* and *DF*, and in the Amoebozoa *Physarum polycephalum* (*PhyP*), *Acanthamoeba castellani* (*AC*) and *Entamoeba histolytica* (*EH*), as well as their closest homologue outside Amoebozoa, determined as described below.

**Figure 1:**
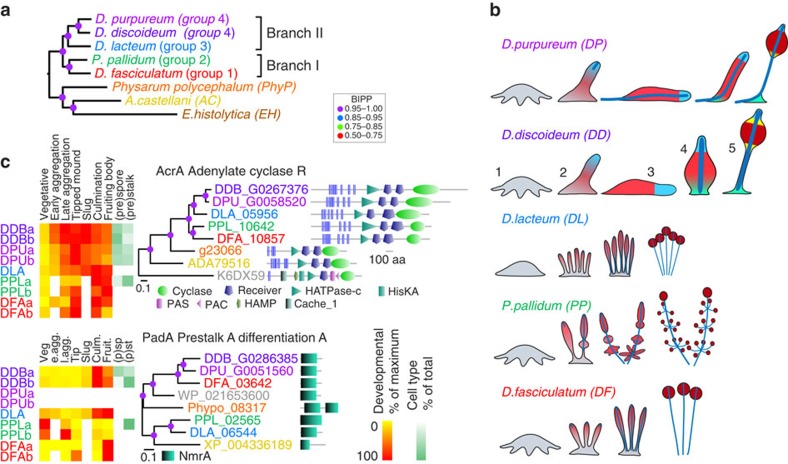
Dictyostelid phylogeny with life cycles and selected annotated gene trees. (**a**) Phylogenetic tree of dictyostelid and unicellular amoebozoan species with sequenced genomes, as inferred from 30 concatenated proteins by Bayesian inference^3^. (**b**) Schematic of life cycle complexity of the Dictyostelid test species. *DF*, *PP* and *DL* form multiple fruiting bodies directly from the aggregate. All cells first differentiate into prespore cells and then form the stalk by dedifferentiation of prespore cells at the tip. *DD* and *DP* form single fruiting bodies from aggregates and display an intermediate migratory ‘slug' in which cells pre-differentiate into prestalk and prespore cells. During fruiting body formation, two more cell types emerge that support the stalk and spore mass^3^,^4^. 1: aggregate, 2: early sorogen (slug), 3: migrating slug, 4: mid-culminant, 5: fruiting body. Light red: prespore; dark red: spore; light blue: prestalk; dark blue: stalk; green: basal disc or supporter; yellow: upper and lower cup. (**c**) Annotated gene trees. Examples of trees, based on the amino-acid sequence of homologues of *DD* DEG, annotated with protein functional domains and developmental expression profiles and inferred by Bayesian inference (see Methods for procedures). The AcrA tree follows the amoebozoan tree topology, identifying all amoebozoan homologs as orthologues. The PadA tree does not, identifying PPL_02565 and DLA_06544 as paralogues of *DD padA*.

Homologues of the *DD* genes were retrieved by BLASTp, and orthology was first assessed from the probability (*E*) values of best bidirectional hits (BBHs), and second from the node structure and branch lengths of trees generated by Bayesian inference of aligned protein sequences. In general, if the node structure of the protein tree follows that of the genome-based Amoebozoan phylogeny (Fig. 1a)^3^, genes were considered to be orthologous. Such correspondence with the Amoebozoan phylogeny is exemplified by the AcrA tree (Fig. 1c). If the homologues group more closely with genes of more distantly related species, or with different *DD* genes, they were likely to be paralogues. This is evident for the PadA tree (Fig. 1c). Conservation of protein length and functional domain architecture further assists the assignment of orthology, as does conservation of developmental regulation (Fig. 1c). However, changes in protein function and gene regulation may also highlight genetic changes in orthologous genes that have given rise to phenotypic innovation across Dictyostelid evolution (see, for example, Fig. 1b). They therefore carry less weight in orthology assessment, but are of considerable interest for evolutionary comparative studies.

To compare developmental gene expression profiles, published time series of high-throughput RNA expression data for *DD* and *DP*^8^ were expressed as percentage of maximal expression and stage-matched to new RNA expression data for *DL*, *PP* and *DF*. The latter three species do not complete development with the same timing as *DD* and *DP*, and we therefore isolated RNAs at the defined stages of growth, early and late aggregation, tipped mound, slug, early culmination and completed fruiting bodies. The developmental programme of *DL*, *PP* and *DF* is also less synchronous than that of *DD* and *DP* resulting in quenching of gene expression peaks. With this in mind, we only considered a gene to be differentially regulated between species when its expression profile clearly differed from profiles of at least two other species. All trees, annotated with protein functional domain architectures and transcription profile schematics, are listed in Supplementary Data 2.

**Figure 2:**
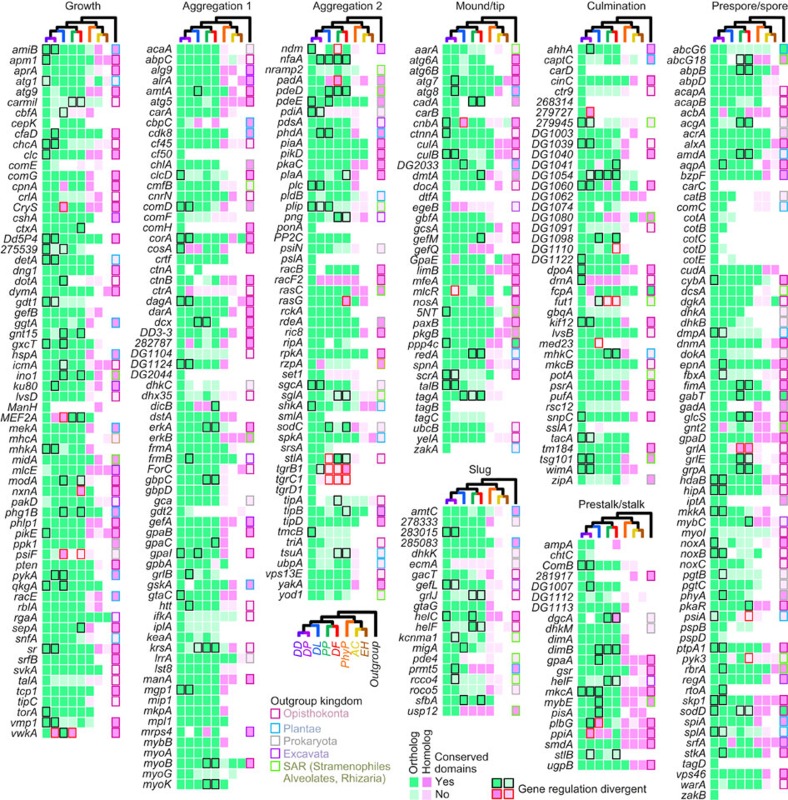
Conservation of 385 *D. discoideum* developmentally essential genes across and outside of Amoebozoa. The presence of validated orthologues and homologues of 385 *DD* DEGs is plotted over the amoebozoan phylogeny and the kingdom outside Amoebozoa with their closest homologue. DEGs are grouped according to the stage, where their null mutant defect is first evident. Box fill colours denote orthology/homology and functional domain conservation. Black and red borders for Dictyostelid orthologues and homologues, respectively, mark genes with developmental expression different from majority (when *n*≥3, all differ from each other). Coloured borders for outgroup species denote kingdom of origin.

**Figure 3:**
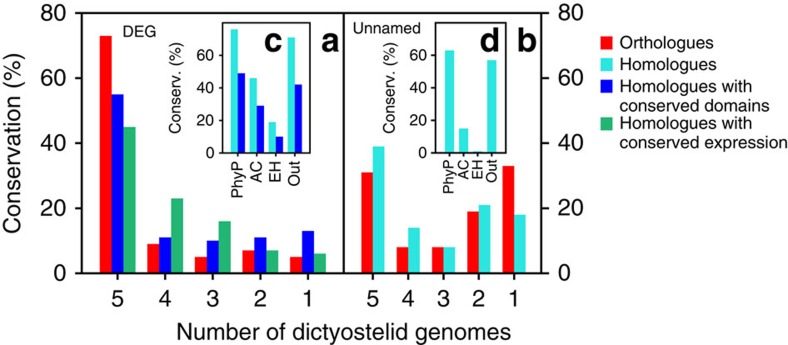
Quantification of conserved features. (**a**) Using the data in Supplementary Data 2, percentages of *DD* DEGs were determined which had orthologues, homologues with conserved domains or homologues with conserved expression over 1 (*DD* only) or 2–5 Dictyostelid genomes. (**b**) For 72 unnamed genes (no known function) at 0.4-Mb intervals along the genome, percentages of orthologues and homologues, only, were determined over 1–5 Dictyostelid genomes. Percentages of homologues and homologues with conserved domains of *DD* DEG (**c**) and unbiased genes (**d**) in each of the three amoebozoan genomes (*PhyP*, *AC* and *EH*) and the non-amoebozoan outgroup. Red: orthologues; light blue: homologues; dark blue: homologues with conserved domains; green: homologues with conserved gene expression.

A summary of the conservation of all DEGs, their functional domain architectures and expression profiles is presented in Fig. 2. Cursory observation shows that the greater majority of DEG is conserved in Dictyostelia, inclusive of their domain architecture and expression, and this is confirmed by quantitation of the number of orthologues, genes with conserved domains and genes with conserved expression profiles (Fig. 3). Seventy-three per cent of DEGs have orthologues in all five Dictyostelid genomes, while 5% of DEGs are present only in *DD*. For 55% of DEG, domain architecture is conserved across Dictyostelia, and for 45% the expression profile is conserved across Dictyostelia (Fig. 3a). The DEGs that regulate different developmental stages show no large differences in these aspects (Supplementary Table 2). Among unicellular amoebozoa, 76% of *DD* DEG have homologues in *PhyP*, followed by 46% in *AC* and 19% in *EH* (Fig. 3c). Despite these low levels of conservation in *AC* and *EH*, most (72%) *DD* DEGs have homologues outside of Amoebozoa, indicating that some amoebozoan taxa underwent considerable gene loss. We compared conservation of *DD* DEGs with a set of genes without known function (Supplementary Fig. 2). Of the latter set, only 31% are conserved as orthologues throughout Dictyostelia, with 33% unique to *DD. PhyP*, *AC* and *EH* genomes contained 63, 15 and 1% homologues, respectively, of *DD* genes with no known roles (Fig. 3b,d). The observation that 73% of DEGs were conserved in Dictyostelia, compared with 31% of genes with unknown function, indicates that genes with proven roles are more likely to be conserved.

### GO term enrichment of *DD* DEGs that are unique to Dictyostelia

**Figure 4:**
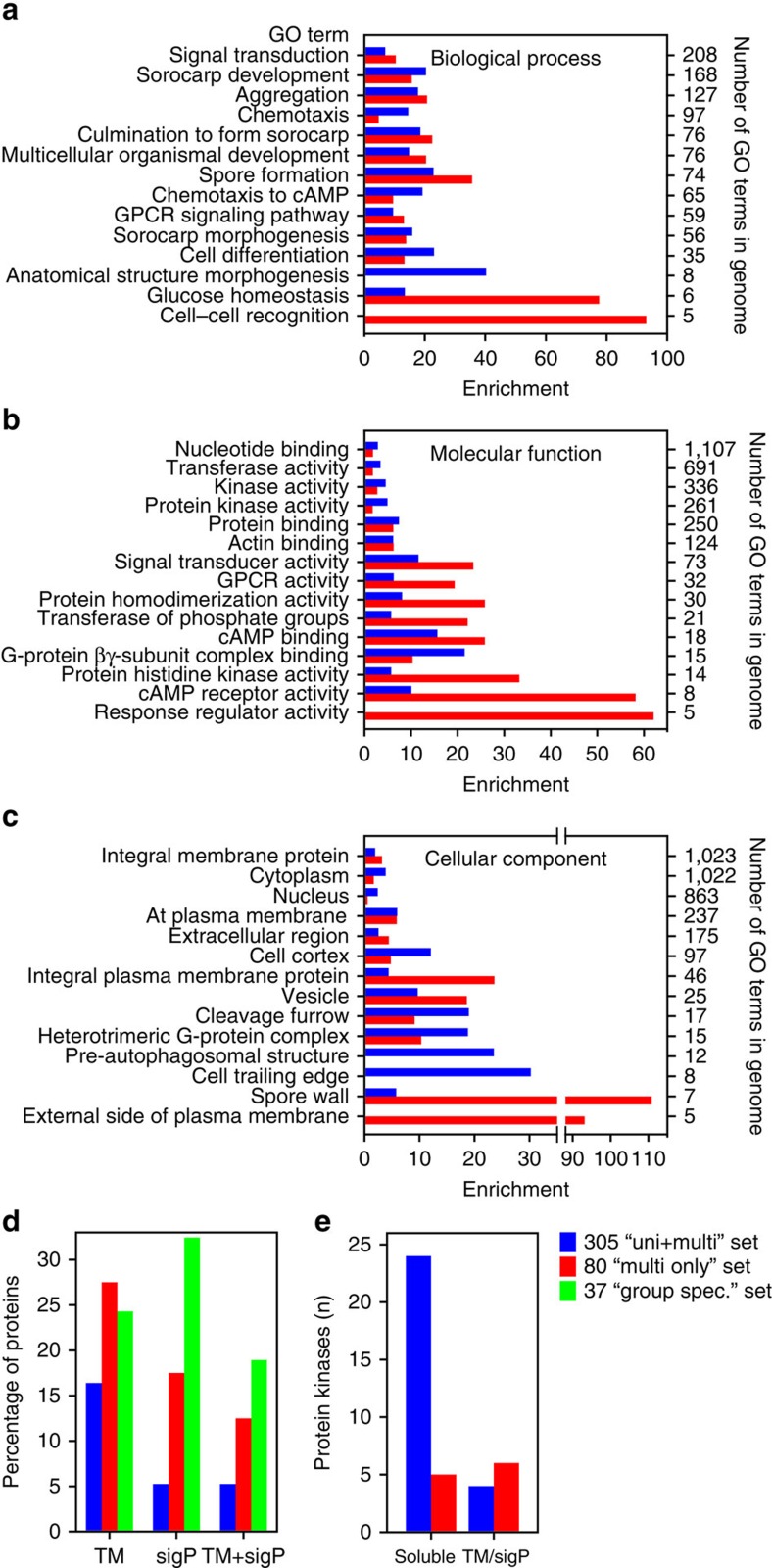
GO enrichment, signal peptides (SigP) and transmembrane (TM) domains. (**a-c**) Enrichment of GO terms in the categories ‘biological process' (a), ‘molecular function (**b**) and ‘cellular component' (**c**). The sets of 305 and 80 DEGs, which are respectively with and without homologues in unicellular amoebozoa, were analysed for GO term enrichment (Supplementary Data 3). The 10 terms with lowest *P* values in each set are compared with the same terms in the other set. The enrichment factor (frequency in set/frequency in genome) for each term is shown. Note that for GO terms that are very abundant in the genome (numbers on right axis) low-enrichment factors are still associated with very low *P* values. (**d**) SigP and TM domains. Proteins in the 305 and 80 sets, as well as 37 proteins with limited conservation within Dictyostelia (green) were analysed with Phobius^63^ for transmembrane domains and signal peptides (Supplementary Data 4). Percentages of proteins with either SigP or TM domains, or with both are presented. (**e**) Protein kinase localization. All protein kinases in the 305 and 80 sets (S/T/Y, histidine and alpha kinases) were analysed with Phobius (Supplementary Data 4) and numbers of proteins in each set without SigP or TM domains (soluble) and with either TM or SigP domains are presented.

A total of 80 *DD* DEGs are absent from unicellular Amoebozoa and are therefore potential ‘multicellularity' genes. We compared enrichment of GO terms between these multi-only DEGs and the remaining 305 uni+multi DEGs (Supplementary Data 3). Not surprisingly, both sets are enriched in development-associated biological process terms, but multi-only has more proteins involved in cell recognition and glucose homeostasis (Fig. 4a). Protein kinases and nucleotide-binding proteins are less prevalent in multi-only, while G-protein-coupled receptors and sensor histidine kinase-associated terms are highly enriched (Fig. 4b). The multi-only set also has less cytoplasmic and nuclear proteins than the uni+multi set, and is enriched in integral plasma membrane, spore wall and external proteins (Fig. 4c). Enrichment of transmembrane and external proteins in the multi-only set is substantiated by computational prediction of signal peptide and transmembrane domains (Fig. 4d; Supplementary Data 4). In multi-only, 58% of proteins have transmembrane domains, signal peptides or both, while for uni+multi this is only 27%. Strikingly, while there are six times more soluble than membrane-associated protein kinases in the uni+multi set, the few protein kinases in the multi-only set are mostly membrane-associated (Fig. 4e).

Overall, it appears that signalling or sensing processes at the plasma membrane are enriched in DEGs, unique to Dictyostelia, while intracellular signal transduction proteins are shared with unicellular Amoebozoa.

### Distribution of conserved features and outgroup homologues

**Figure 5:**
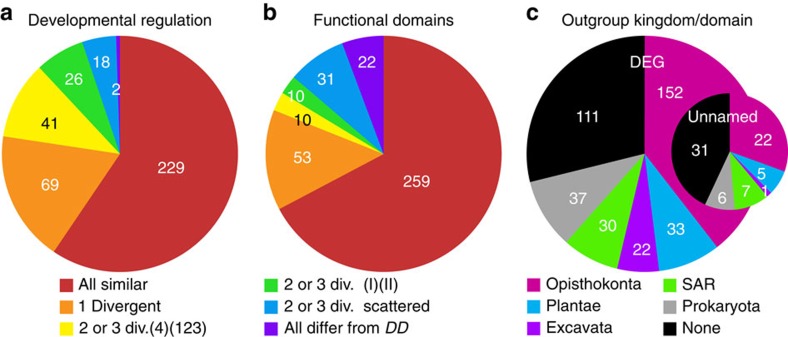
Phylogenetic distribution of conserved features and outgroup homologues. (**a**) Gene expression. Phylogenetic distribution of numbers of DEG with different patterns of conservation of gene expression. (4)(123) signifies that developmental expression was different between groups 4 and 1–3, while (I)(II) signifies different expression between branches I and II (Fig. 1a). (**b**) Protein domain architecture. Phylogenetic distribution of DEG with different patterns of functional domain conservation. (**c**) Origin of outgroup homologues. Numbers of DEG (out of 385) without no homologues outside Amoebozoa, or with outgroup homologues in each of the eukaryote kingdoms or in prokaryota. Inset; the same out of 72 unnamed genes.

Between Dictyostelia, gene expression profiles were generally conserved, with 69 being different in one species (Fig. 5a), 41 being different in group 4 and only 26 being different between the early diverging branches I and II (Fig. 1a). The prevalence of divergent gene regulation in group 4 is correlated with the major phenotypic innovation that occurred in this group^3^,^4^ (Fig. 1b). Functional domain architecture is also well conserved, but when different, the affected genes are more scattered across the phylogeny, suggesting no obvious links between domain change and phenotypic change (Fig. 5b).

For *DD* DEGs with homologues outside Amoebozoa, the sister kingdom Opisthokonta provided 152 outgroup homologues (Fig. 5c), while the remaining 122 were about equally distributed over other eukaryote kingdoms and prokaryotes. Eleven *DD* DEG (*acgA*, *cadA*, *chlA*, *dgcA*, *dhkA*, *dhkB*, *dhkC*, *dokA*, *iptA*, *phyA* and *ppk1*) have closest homologues in prokaryotes, but none in unicellular amoebozoa, suggesting acquisition by LGT. To validate these putative cases of LGT, we combined bidirectional searches with phylogenetic inference to assess whether the *DD* gene was indeed more closely related to its prokaryote homologue than to any eukaryote gene outside of Dictyostelia. The results of these analyses are listed in Supplementary Data 5 and summarized in Table 1.

For *acgA*, *dhkA*, *dhkB, dhkC, phyA* and *ppk1*, LGT appeared to have occurred more deeply in the eukaryote lineage, while for *cadA*, its homologue *cadC* is the more likely candidate for LGT. *DgcA* is very prevalent in bacteria and therefore a common contaminant of eukaryote genome sequences. However, in such cases, the LGT candidates are neither transcribed nor present in close relatives of the eukaryote host. The Dictyostelid *dgcA*s are present in all Dictyostelid genomes and were shown to have a biological role in *DD*^13^. *ChlA*, *dokA* and *iptA* also appeared to be genuine cases of LGT. For *iptA*, this was already proposed previously^5^. DgcA, chlA and iptA encode the enzymes that respectively synthesize c-di-GMP, DIF-1 and discadenine (Table 2), which, with cyclic AMP (cAMP) and 4-Methyl-5-pentylbenzene-1,3-diol (MBPD), represent all known differentiation-inducing signals in *DD* that are not of peptide origin^1^,^14^.

### Non-conserved DEGs

Genes that have a proven function in *DD*, but limited conservation in other Dictyostelia are of interest, since their appearance in the course of Dictyostelid evolution may have caused group-specific phenotypic innovation. Table 3 summarizes 37 *DD* DEGs that are unique to *DD*, to group 4 (*DD+DP*) or to branch II (*DD+DP+DL*). Twenty-six of these proteins contain a signal peptide or single transmembrane domain, indicating they are either secreted or exposed exteriorly. High enrichment of exteriorly exposed proteins was also evident for *DD* DEGs that are unique to Dictyostelia, indicating that this class of proteins is most frequently subjected to gain, loss and/or major modification in the course of multicellular evolution. Among them are four spore coat proteins (CotA, B, C and E), five matrix proteins with CTDC domains and three Tgr adhesion proteins. These three types of exposed proteins belong to large variable families that are present in all Dictyostelia, *PhyP* and for Tgrs also *AC* (Table 4). Binding of compatible Tgr variants mediates kin recognition, excluding unrelated cells from participating in the same fruiting body^15^,^16^. In aggregative multicellularity, this is an important feature, since unrelated cells entering the aggregate can cheat by forming predominantly spores and not stalk cells. In case of obligate cheaters, lack of stalk cell differentiation could lead to the collapse of multicellularity^17^,^18^. The mutual adhesiveness of cells with compatible Tgr proteins not only keeps them together in one aggregate, but also triggers signalling pathways that are required for post-aggregative development^19^. Obviously, for these proteins species specificity is essential for their function. Among the matrix proteins with CTDC domains, four (pdiA, psiA, psiF and psiN) also act as signals or signal regulators.

Among the other exposed DEGs are the two countin proteins, CtnB and CtnC, which are unique to group 4. With CtnA, these proteins are components of the counting factor complex of secreted proteins that regulates aggregate size^20^. Interestingly, SmlA and SslA1, two proteins that regulate secretion or processing of counting factors^21^,^22^ are also unique to group 4, suggesting that size regulation by counting factor complex was elaborated in this group. The remaining exposed/secreted proteins, ChtC, RtoA and SrsA, have roles in social cooperation^23^, cell-type proportioning^24^ and timely aggregation^25^, respectively, while two others, DDB_G0279727 and DDB_G0 268314, are needed for normal fruiting body formation^26^. None of these proteins have orthologues outside group 4, and their roles must also be recent.

Three intracellular proteins, DtfA, PonA and ManH, which are required for tip formation^27^, efficient chemotaxis^28^ and normal growth and development^26^, respectively, are unique to *DD.* Another intracellular protein, IptA, which synthesizes the spore germination inhibitor discadenine^29^, is unique to group 4. This agrees with the absence of discadenine from non-group 4 species^30^. CatB, a catalase required for H_2_O_2_ resistance of spores^31^, is an innovation of branch II.

Some transmembrane proteins involved in signal processing also show limited conservation. They are the cAMP phosphodiesterase Pde4, which is only present in branch II and is required for normal late development^32^, the ABC transporters TagB and TagD, which duplicated from TagC in *DD* only. TagB is with TagC required for prestalk differentiation^33^. The cAMP receptors CarB, CarC and CarD are also unique to group 4, with only CarA orthologues being present in groups 1–3, as evident from gene synteny and phylogenetic inference^34^,^35^. However, single and double gene duplications of CarA occurred in groups 2 and 1, respectively (Supplementary Data 2). *DD* CarB is essential for development beyond the mound stage^36^, while CarC and CarD have opposite effects on prespore/prestalk patterning by activating and inhibiting GSK3, respectively^37^,^38^. The duplicated CarA genes in *PP* have overlapping roles in fruiting body morphogenesis and induction of prespore differentiation by cAMP^34^,^35^, indicating that the roles of cARs B,C and D are novel to group 4. Zak1 and Zak2, two tyrosine kinases with overlapping roles in mediating CarC activation of GSK3 (refs 39, 40), are also unique to *DD* (Supplementary Data 2), as is the nuclear factor PslA. CarC, Zak1, Zak2, GSK3 and PslA are all proposed to mediate cAMP-induced prespore gene expression^37^,^39^,^41^,^42^, a process that also occurs in *PP*^34^, but evidently without CarC, Zak1, Zak2 or PslA. One study found no requirement for GSK3 in cAMP-induced prespore gene expression in *DD*^43^, while another excluded an essential role for CarC^44^. This evidence, together with the lack of most pathway components in non-group 4 species, encourages further study of this central pathway for Dictyostelid survival. The essential role of CarB in post-aggregative morphogenesis in *DD* is also enigmatic. To test whether its role may have been erroneously assigned, we recreated a *carb* null mutant in two different parent strains AX2 and AX3. Supplementary Figure 3 shows that both the AX2 and AX3 *carb* null mutants develop normally to fruiting bodies, indicating that *carB* is not of major importance for *DD* development. Evidently, essential central roles assigned to poorly conserved genes require careful scrutiny.

## Discussion

The *DL* genome completes the set of high-quality genome sequences representative of the four major groups of Dictyostelia. Earlier comparative genome analyses have focussed on conservation and change in gene families, of which at least some members have established functions in biological processes. These analyses provided global information on differences in protein-coding potential between *Dictyostelium* taxon groups, and between Dictyostelia and other eukaryotes^5^,^6^,^7^. We seek to understand the genetic changes that caused the evolution of multicellularity and cell-type specialization, combining results from comparative genome analysis with detailed comparative phenotypic mapping of Dictyostelia^3^,^4^. In this work, we therefore investigated genes that have an established role in multicellular development of *DD*, and compared their conservation and change across the group-representative Dictyostelid genomes and three genomes of unicellular Amoebozoa. Not all genes in this set of 385 DEGs will control development directly in a regulatory manner. Many genes have unknown molecular functions; several, such as the spore coat genes, define the phenotype of the differentiated cells; others, such as cytoskeletal components, enable cellular movements that cause morphogenesis. However, since the boundaries between these categories are not clearly defined, we cannot meaningfully use them to assign subsets. We have however excluded all genes that are essential for growth, although the 385 set contains 66 DEGs that in addition to their developmental defects also show mild growth defects, when deleted.

A major outcome of the work is that a large percentage (73%) of *DD* DEGs is conserved as orthologues across all Dictyostelid taxon groups, while 76% of DEGs have orthologues or homologues in *Physarum*, the closest unicellular relative of Dictyostelia. This finding indicates that most of the genes required for multicellular development were already present in the last common unicellular ancestor to Dictyostelia. The conserved DEG have mostly similar transcription profiles across species, but for those which have not, differences occur more frequently between groups 4 and 1–3 than between the more distantly related branch I (groups 1 and 2) and branch II (groups 3 and 4). Phenotypic mapping revealed that group 4 is also phenotypically most different from groups 1–3 (refs 3, 4). The 41 genes with different regulation in group 4 therefore deserve deeper study into possible involvement in the phenotypic innovation in this group. For one gene, *carA*, this role was already established. This gene, encoding the chemotactic cAMP receptor, is expressed both during and after aggregation in group 4, but only after aggregation in groups 1–3 (ref. 35). Pre-aggregative expression of *carA* in group 4 is associated with the use of cAMP as chemoattractant for aggregation in this group^3^,^34^.

Changes in gene function may also cause phenotypic innovation, and such changes could be evident from changes in the functional domain architecture of proteins. However, in this case, only 10 architectures were different between group 4 and 1–3, and an equal number between branch I and II. Many more (51) diverging architectures were scattered across the Dictyostelid phylogeny, suggesting that the change in gene function, assessed by this metric, is not correlated with the phenotypic change. It therefore appears that in Dictyostelia, the phenotypic change is more closely correlated with the changes in gene regulation than the changes in gene function. Morphological evolution in metazoa was proposed to be caused more frequently by the changes in gene expression rather than gene function and several instances of the former were reported^45^.

Among the genes with limited conservation within Dictyostelia, 70% encode secreted or exposed proteins with roles in cell recognition, adhesion or signalling. Many of the remaining genes cause only minor aberrations when deleted, while among the set of poorly conserved genes with essential roles, there may be some for which this role was assigned in error. By repeating a gene knockout experiment for the group 4 specific gene *carB*, we show that the essential role of *carB* in post-aggregative development was overstated.

For most *DD* DEGs, the closest homologues outside Amoebozoa are found in Opisthokonta, the sister group to Amoebozoa, with the remaining homologues being about equally distributed among each of the other eukaryote divisions and prokaryotes. Four genes that are present in Dictyostelia, but not in unicellular Amoebozoa, entered Dictyostelia by LGT from prokaryotes. Three of these genes, *dgcA*, *chlA* and *iptA*, encode enzymes that synthesize three out of the five known non-peptide signals in *DD* that induce cell differentiation.

The set of 80 DEGs that are present in Dictyostelia, but not in unicellular Amoebozoa, is strongly enriched in proteins that are either secreted or exposed to the cell exterior and have roles in cell adhesion, sensing and cell–cell recognition. In contrast, the set of DEGs that are also present in unicellular Amoebozoa is enriched in cytosolic and nuclear proteins, with roles in nucleotide binding and protein kinase activity. The enrichment of secreted or exteriorly exposed proteins in DEGs unique to Dictyostelia suggests that the transition from uni- to multicellularity mostly required novel genes that mediate cell–cell interactions, while the intracellular machinery for processing these interactions was already present in the unicellular ancestor. Similar findings were also reported for distinctions between the multicellular metazoa and their unicellular holozoan relatives. Intracellular signal transduction components are generally well conserved in holozoa, while externally exposed proteins and sensors are much more diversified in the metazoa^46^,^47^,^48^. This distinction is particularly striking for the tyrosine kinases, where the cytosolic kinases, but not the receptor tyrosine kinases, are mostly conserved in holozoa^48^. Also in Amoebozoa, the few protein kinases that are unique to the multicellular Dictyostelia are mostly membrane associated (Fig. 4e).

Many of the secreted and exposed proteins that are unique to Dictyostelia or taxon groups within Dictyostelia are members of large families of adhesion and matrix proteins, which also have members in unicellular Amoebozoa. In the unicellular forms, they have likely roles in adhesion to substrata, mates and prey. We propose that these families are the reservoirs from which primordial multicellularity genes, mediating cell–cell adhesion, were recruited. For the next step, cell-type specialization, Dictyostelia converted some matrix/adhesion proteins, such as PsiA, PsiF, PsiN, TgrB and TgrC into signal molecules, but additionally acquired some genes to synthesize differentiation-inducing signals by LGT from their bacterial prey.

The importance of matrix proteins for the evolution of multicellularity is also demonstrated by experiments showing that under simple gravity selection, unicellular yeast and green algae evolve into multicellular agglomerates within months, accompanied by increased adhesion for yeast and matrix deposition for algae^49^,^50^. We show here that most genes required for multicellular development were already present in unicellular relatives or were recruited from existing matrix/adhesion protein families. Combined, these separate approaches show that the major evolutionary transition from uni- to multicellularity depended more on environmental pressures than massive genetic change.

## Methods

### Genome sequencing and assembly

*DL* cells, grown in association with *Escherichia coli*, were freed from bacteria and starved for 6 h. For genomic DNA isolation, 10^8^ cells were resuspended in 0.5 ml 10 mM K-phosphate, pH 6.5, and cells were lysed by slowly adding the suspension to 3 ml ice-cold LB (0.32 M sucrose, 5 mM MgCl2, 1% Triton X-100 in 10 mM Tris (pH 7.5)). Nuclei were precipitated by centrifugation for 10 min at 7,500*g*, washed once and resuspended in 50 μl LB. The suspension was successively mixed with 0.5 ml of 10 μg ml^−1^ RNAseA and 10 mM EDTA in 10 mM Tris (pH 7.5), and 0.5 ml of 200 μg ml^−1^ proteinase K and 0.7% SDS in 10 mM Tris (pH 7.5), and incubated for 1 h at 65 °C and 1 h at 37 °C. After addition of 21 μl 5 M NaCl, the suspension was extracted with 1 ml 1:1 phenol/chloroform and once more with chloroform. DNA was precipitated by addition of 3.5 ml absolute ethanol to the supernatant fraction. DNA libraries for 454 Roche sequencing were prepared according to the manufacturer's protocols. Libraries were sequenced, using a Roche flx platform, yielding 1.5 million raw reads, which added up to a genome coverage of 29 × . Initial assembly was performed with the Newbler assembler^51^ and contigs >500 bases were entered in the Staden assembly package^52^, including the Newbler-derived quality values. Fosmid libraries with average insert sizes of 32.5 kb in vector pCC2FOS were prepared according to the manufacturer's instructions (Epicentre Biotechnologies). Fosmid ends were sequenced using the ABI BigDye kit, and standard forward primer 5′-GTACAACGACACCTAGAC-3′ and reverse primer 5′-CAGGAAACAGCCTAGGAA-3′. Pre-assembly trimming of sequences was performed with Phred^53^. The fosmid reads were mapped to the genome assembly using BLAT^54^. The fosmid end sequence information was used to assemble the contigs into scaffolds. Gaps in the scaffolds were closed by primer walking. The assembly and gap closure procedure resulted in 54 gap-free contigs spanning 23.4 Mb.

### Chromosome structure analysis

The consensus sequences of the telomere repeats were used to search for contigs and single reads containing these repeats using BLASTn in the whole *DL* genome. The assembly of all contigs with such repeats was checked manually for wrongly assembled reads, since repetitive sequences tend to cause faulty assembly. Consensus sequences of the *DD* repetitive elements were used as query to find similar sequences in the genomes of the other social amoebae. Regions adjacent to telomeres were searched for other repeated sequence motifs. Detected repeat units were used next as query to find additional identical sequences throughout the genome.

### Gene model prediction

Protein-coding genes were predicted using Augustus^55^ after training with *DL* transcript data obtained by Illumina sequencing (Supplementary Data 6). Specifically, single transcript reads were aligned to the reference genome sequence using BLAT^54^ and the resulting aligned positions were filtered against spurious hits. Intron–exon boundaries were extracted from the resulting alignments and used as a training set for Augustus. In addition, coding sequences from defined orthologues covered by transcript reads were used as a training set to define coding sequence features. The gene models used for comparative analysis in this manuscript were manually curated when alignment with orthologues indicated the presence of non-conserved indels.

### Identification of orthologues of DEGs across species

DEGs are defined as *DD* genes, for which deletion causes defects in multicellular development. Around 385 DEGs were retrieved from Dictybase^12^. To identify orthologues of *DD* DEGs in other *Dictyostelium* species, unicellular Amoebozoa and other kingdoms, we first used Blastp to identify BBHs by query of Genbank for *AC*, *EH* and non-amoebozoan proteins, and query of locally generated libraries for *DP*, *DL*, *PP*, *DF* and *PhyP* proteins. The *DP* protein sequences were downloaded from Dictybase (http://dictybase.org/) and the *Physarum* library was derived from the translated *Physarum* reference transcriptome^11^. When proteins were missing in a single species, the query was repeated with a tBlastn query of genome sequence to retrieve coding sequences that had escaped computational gene model prediction.

The BBH approach cannot distinguish between orthologues and paralogues with full confidence, particularly when genes are members of larger families. We therefore used Bayesian phylogenetic inference (see below) to determine relatedness between protein sequences, and assessed orthology from the node structure and branch lengths of the inferred phylogenies. Genes were validated as orthologues, when their phylogenetic position and branch length relative to that of the *DD* DEG mirrored that of the amoebozoan species tree, constructed from 30 concatenated protein sequences^3^. The term homologue comprises both paralogues and orthologues that could not be validated because tree nodes were not well resolved or genes had undergone recent duplications. The phylogenies were annotated with the functional domain architectures of the proteins using SMART^56^, which provided additional evidence for evaluation of orthology.

### Protein phylogenies

Protein sequences were aligned using Clustal Omega with five combined iterations^57^. Poorly aligned sections were deleted and phylogenetic relationships were determined using MrBayes v3.1.2 (ref. 58) after model selection in Topali^59^. Analyses were run for 1 million generations or until the standard deviation (SD) of split frequencies <0.01. Trees were midpoint rooted using Figtree v1.4.2 (ref. 60).

### Comparative transcriptomics

For the group 4 species, *DD* and *DP*, replicate gene expression profiles that cover the 24 h developmental programme, as well as genes expressed in purified prestalk and prespore cells, were prepared by others^8^. The development of *DL*, *PP* and *DF* mostly takes longer than 24 h and is less synchronous than in group 4. RNAs were therefore isolated at specific stages of development, rather than at regular time intervals. Cells were cultured on one-fifth SM agar in association with *E. coli*, collected and distributed at 5 × 10^5^ cells per cm^2^ on non-nutrient agar. Plates were incubated at 22 °C with charcoal pellets in the lids to improve synchronous development. RNA was isolated before plating cells (growth), at early and completed aggregation, tipped mound and slug stages, and at early and completed fruiting body formation. For *PP*, RNAs were also isolated from purified spores and stalks. First, unencapsulated cells from fruiting bodies were lysed with 0.1% Triton X-100 and next spores were separated from stalks by sieving though a 10-μm mesh. RNAs of amoeboid cells were isolated using the Qiagen RNAeasy kit. Fruiting bodies, spore and stalk samples were additionally vortexed for 15 min with glass beads in the first cell lysis step to break the cell walls. Poly-A RNA was isolated from ∼1 μg total RNA, and reverse-transcribed into complementary DNA (cDNA) using a random priming cDNA Synthesis kit (Roche).The cDNA was converted into a sequencing library for 454 sequencing using the GS FLX Titanium Rapid Library Preparation kit and sequenced on the Roche 454 platform. The resulting sequencing reads were mapped to the genome using BLAT^54^, and realigned with Exalign^61^ to determine the intron/exon boundaries. The expression levels were normalized by dividing the matching reads by the total number of reads obtained for each sample, multiplied by two times the average read length. The results are listed in Supplementary Data 6. Replicate developmental time series of *PP*, *DL* and *DF* were prepared in the same manner, but sequenced using the Illumina HiSeq platform. The results of these experiments are listed in Supplementary Data 6.

To compare developmental gene expression profiles between species, published expression data for *DD* and *DP*^8^ were stage-matched to new RNA expression data for *DL*, *PP* and *DF*. Normalized read counts from the individual time series were expressed as percentage of the maximum read count in the series. Normalized read counts for purified prestalk and prespore cells (*DD* and *DP*) or purified stalks and spores (PP) were expressed as percentage of the sum of the (pre)stalk and (pre)spore counts. For presentation, these percentages were converted into heatmaps as shown in Fig. 1c and Supplementary Data 2.

### Validation of LGT

To validate putative instances of LGT, we used the criteria that Dictyostelid genes should first have prokaryote genes as BBHs and second should have prokaryote genes as closest sister group in phylogenies constructed from the closest prokaryote and eukaryote homologues. To identify BBHs, we first used the *DD* protein sequence as bait to query all non-redundant genes in Genbank, with the exclusion of Dictyostelia. When the best hits of the search were prokaryotes, the top prokaryote hit was used to query all non-redundant eukaryote genes. The first 10–20 eukaryotes hits were then aligned with the *DD* protein and the top hits from the first query as described above for protein phylogenies. The alignment was subjected to phylogenetic inference using MrBayes v3.1.2 (ref. 58) after model selection in Topali^59^. Analysis were run for 1 million generations or until the SD of split frequencies <0.01. Trees were also inferred using RAXML in Topali^59^ with 100 bootstrap replicates after model selection. All BlastP output files and the unrooted trees are shown in Supplementary Data 5.

### *CarB* knockout

A 1.25-kB *carB* fragment was amplified from genomic DNA using primers *carB* sense (5′-AATTAAAAAATGACTATTATGTCAGAT-3′) and *carB* antisense (5′-TTATCACTTTTAAA TCATATCATTTTT-3′) and cloned blunt-end into a TOPO pCR2.1 plasmid vector. The actin15 blasticidin resistance cassette was obtained by digestion of pUCBsrΔBam^62^ with BamHI and HindIII, filled in with Klenow and blunt-end cloned into the filled in unique NdeI site of the *carB* fragment. For gene disruption, a knockout construct with the Bsr cassette in reverse orientation was amplified using the *carB* sense and *carB* antisense primers and transfected into *DD* AX2 and AX3 cells by electroporation. Gene disruption was confirmed by PCR with test primer carB-out (5′-AAAAAAACATCCCGAACA-3′) outside the knockout construct and primer Bsr2 (5′-AGCATTGTAATCTTCTCTGTCGCTACTTCTACT-3′) inside the blasticidin resistance cassette (Supplementary Fig. 3).

### Data availability

The *DL* genome can be browsed in SACGB (http://sacgb.fli-leibniz.de/cgi/index.pl). The whole *DL* genome shotgun project has been deposited at DDBJ/EMBL/GenBank under the accession LODT01000000.1

## Additional information

**How to cite this article:** Glöckner, G. *et al.* The multicellularity genes of dictyostelid social amoebas. *Nat. Commun.* 7:12085 doi: 10.1038/ncomms12085 (2016).

## Supplementary Material

Supplementary Information Supplementary Figures 1-3, Supplementary Tables 1-2 and Supplementary References

Supplementary Data 1 Spreadsheet listing all *D. discoideum* genes analysed in this work with their knock-out phenotypes, protein function and homologs in Amoebozoa

Supplementary Data 2 Phylogenies of *D. discoideum* developmentally essential genes and their homologs, annotated with domain architectures and developmental expression profiles

Supplementary Data 3 Spreadsheet listing gene ontology terms that are enriched in the set of developmentally essential genes that are present in both unicellular Amoebozoa and Dictyostelia and the set that is only present in Dictyostelia

Supplementary Data 4 Spreadsheet listing the presence of transmembrane domains and signal peptides in four subsets of *D. discoideum* developmentally essential genes

Supplementary Data 5 Spreadsheet listing the BLASTp queries and phylogenetic trees that provide the evidence in favour or against instances of lateral gene transfer for 11 *D. discoideum* genes

Supplementary Data 6 Sequencing statistics and read counts for high throughput RNA sequencing of *P. pallidum*, *D. fasciculatum* and *D. lacteum* developmental time courses

## Figures and Tables

**Table 1:** Analysis of candidate genes for lateral gene transfer.

**Gene**	**BBH with prokaryote gene**	**Closest relative is prokaryote**		**LGT**
		**Bayes**	**RAXML**	
acgA	No	No	No	No
cadA	No	Unclear	No	To cadC
chlA	Yes	Yes	Yes	Yes
dgcA	No*	Yes	Yes	Yes
dhkA	No	No	No	No
dhkB	No	No	No	No
dhkC	No	Yes	Yes	Unlikely
dokA	Yes	Yes	Yes	Yes
iptA	Yes	Yes	Yes	Yes
phyA	No	No	Yes	Unlikely
ppk1	No	No	Yes	Unlikely

BBH, best bidirectional hit; LGT, lateral gene transfer.

The listed genes all have their closest homologues in prokaryotes. BlastP query was used to assess whether the prokaryote homologue also had the *Dictyostelium* gene as best hit in eukaryotes (BBH). Next, Bayesian and maximum likelihood (RAXML) phylogenetic inference of prokaryote and eukaryote sequences that were most similar to the *Dictyostelium* gene and its prokaryote homologue were used to assess whether the *Dictyostelium* gene grouped with prokaryote genes rather than with other eukaryote genes. All analyses are listed in Supplementary Data 5.

^*^The top hits were bacterial contaminants of eukaryote genomes.

**Table 2:** *Dictyostelium* DEGs with closest homologues in prokaryotes.

**Gene**	**Essential for:**	**Molecular function**	**Catalyses/detects**	**References**
*chlA*	Basal disk differentiation	Flavin-dependent halogenase	DIF-1 chlorination	64,65
*dgcA*	Stalk cell differentiation	Diguanylate cyclase	c-di-GMP synthesis	13
*dokA*	Osmotic stress resistance and spore differentiation	Sensor histidine kinase	Osmolytes	66
*iptA*	Spore differentiation and spore dormancy	Isopentenyltransferase	Discadenine synthesis	29,67

DEGs, developmentally essential genes.

The table lists the biological role and molecular function of all *DD* DEGs that were acquired by lateral gene transfer from prokaryotes.

**Table 3:** Molecular functions of *DD* DEGs with limited conservation.

**Gene**	**Molecular function**	**S/T**	**Only**
*ampA*	Unknown	S	4
*carB*	cAMP receptor	T	4
*carC*	cAMP receptor	T	4*
*carD*	cAMP receptor	T	4*
*catB*	Catalase B	−	II
*chtC*	Unknown	T	4
*comC*	Has EGF domains	S+T	4
*comH*	GATA zinc finger	−	*DD*
*cotA*	Spore coat protein	S	II
*cotB*	Spore coat protein	S	4
*cotC*	Spore coat protein	S	4
*cotE*	Spore coat protein	S	II
*ctnB*	Has saposin domain	S	4
*ctnC*	Has saposin domain	S	4
*DDB_G 0268314*	Has EGF-like domains	T	*DD*
*DDB_G 0279727*	Unknown	S	4
*dtfA*	Unknown	−	*DD*
*ecmA*	Matrix protein	S	*DD*
*iptA*	Isopentenyltransferase	−	4
*manH*	Beta-mannosidase	−	*DD*
*pde4*	cAMP phosphodiesterase	T	II
*pdiA*	PdsA inhibitor	S	*DD*
*ponA*	Anchors actin to plasma membrane	−	*DD*
*psiA*	Secreted signal	S	II
*psiF*	Exposed signal	S	4
*psiN*	Exposed signal	S	4
*pslA*	Nuclear protein	−	*DD*
*rtoA*	Catalyses vesicle fusion	S	4
*smlA*	Unknown	−	4
*srsA*	Unknown	T	II
*sslA1*	Unknown	−	*DD*
*tagB*	ABC transporter	T	*DD*
*tgrB1*	Cell adhesion	S+T	4
*tgrC1*	Cell adhesion	S+T	*DD*
*tgrD1*	Cell adhesion	S+T	*DD*
*zak1*	Tyrosine kinase	−	*DD*
*zak2*	Tyrosine kinase	−	*DD*

cAMP, cyclic AMP; *DD*, *D. discoideum*; *DP*, *D. purpureum*; DL, *D. lacteum*; DEGs, developmentally essential genes.

*DD* DEGs with limited conservation across Dictyostelia are listed with their experimentally determined (underlined) or bioinformatically inferred (regular text) molecular function, presence of signal peptide (S) or transmembrane domain (T) and their conservation in *DD* only, group 4 (*DD*+*DP*) or branch II (*DD*+*DP*+*DL*). The phenotypes associated with deletion of these genes in *DD* are listed in Supplementary Data 1, while annotated gene trees are shown in Supplementary Data 2.

^*^Genes were detected by PCR in several group 4 species^34^.

**Table 4:** Amoebozoan families of exposed and secreted proteins.

***Type*****/examples**	**Domain**	**Number of proteins per genome**				
		***DD***	***DL***	***PP***	***PhyP***	***AC***
*Matrix*						
* *ecmA, ecmB, pdiA, psiA, psiF and PsiN	Dicty_CTDC	11	6	15	10	0
	PA14	21	12	4	57	0
	both	3	1	2	5	0
						
*Spore coat*						
* *cotA-E, pspB, pspD and sigD	Spore_N	2	4	3	1	0
	FOLN	4	3	13	1	0
	Both	7	4	3	0	0
						
*Cell adhesion*						
tgrB1, tgrC1 and tgrD1	IPT/TIG	55	76	110	42	5
	EGF-like	33	15	25	114	24
	Both	5	7	1	1	0

*DD*, *D. discoideum*; *DP*, *D. purpureum*; *DL*, *D. lacteum*; *PP*, *Polysphondylium pallidum*.

Species proteomes were queried for protein functional domains by Interproscan 5 (ref. 68) and all proteins that contain the domains listed above were isolated and counted. Interpro identifiers and full domain names: Dicty_CTDC, *Dictyostelium* (slime mold) repeat: IPR001673; PA14: IPR011658; Spore_N, Dictyostelium spore coat protein, N terminal: IPR007643; FOLN, Follistatin-like, N terminal: IPR003645; IPT/TIG, immunoglobulin-like fold domain: IPR002909; EGF (epidermal growth factor)-like comprises: EGF-like calcium-binding domain: IPR001881; EGF-like domain, extracellular: IPR013111; EGF-like, conserved site: IPR013032; and EGF-like, laminin: IPR002049.
