# Supplementary material for: The multicellularity genes of dictyostelid social amoebas
Source: Nat Commun. 2016 Jun 30;7:12085. doi: 10.1038/ncomms12085 (PMC4931340; doi:10.1038/ncomms12085)
Supplement: Supplementary Information — Supplementary Figures 1-3, Supplementary Tables 1-2 and Supplementary References [file ncomms12085-s1.pdf]

Supplementary Figures

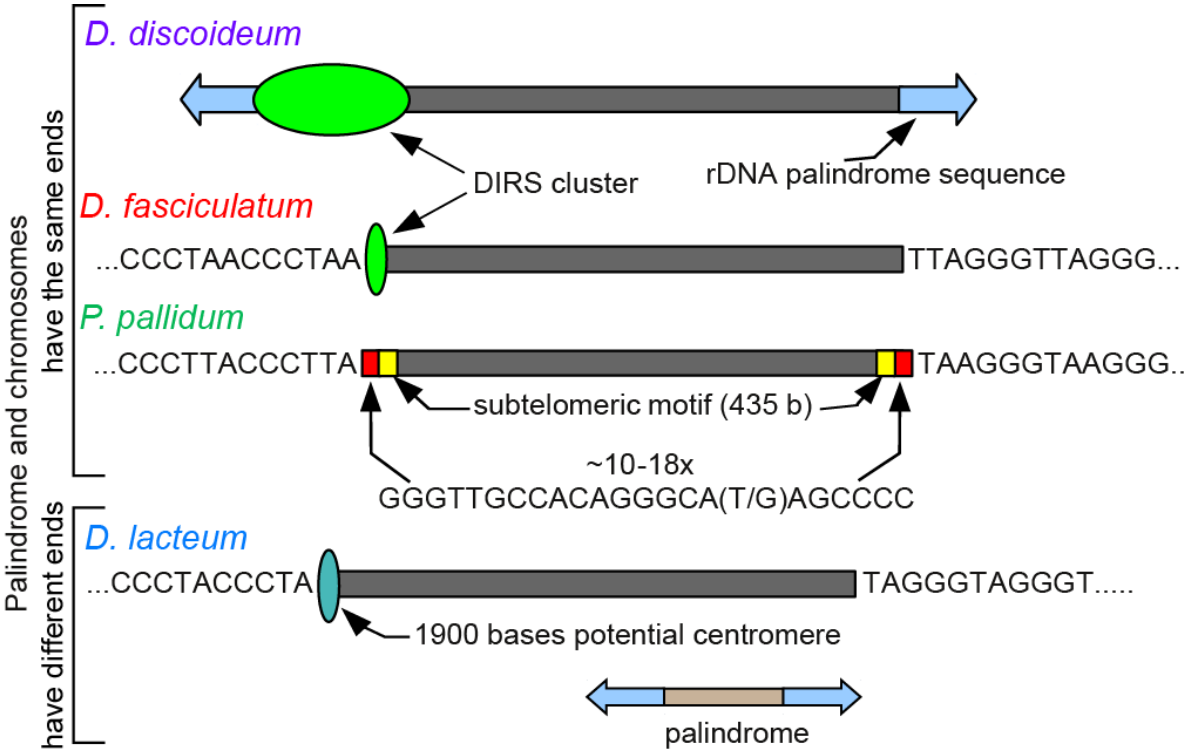

Supplementary Figure 1. Chromosome structure.
Previous studies of Dictyostelid genomes highlighted large differences in chromosome structure between *DD* on one hand and *PP* and *DF* on the other[1,2]. *DD* chromosomes have no eukaryote-type T/G-rich telomeric repeats, but instead harbour long A/T rich repeats at chromosome ends, which also make up the ends of the extrachromosomal rDNA palindrome. The centromere consists of a large cluster of DIRS transposable elements. In *PP* and *DF* both the chromosomes and the palindrome have eukaryote-type T/G-rich telomeric repeats. *DL*, which occupies a phylogenetic position in between *DD* on one hand, and *DF* and *PP* on the other, shows an intermediate situation with canonical eukaryote chromosome telomere repeats and *DD*-type A/T-rich repeats at the palindrome ends. A small DIRS cluster may represent the centromere in *DF*, while six A/T-rich 1900 nt regions were detected in the *DL* genome, which may represent the *DL* centromeres. *PP* has no recognizable centromere region, but chromosomes harbour conserved subtelomeric motifs at both ends.

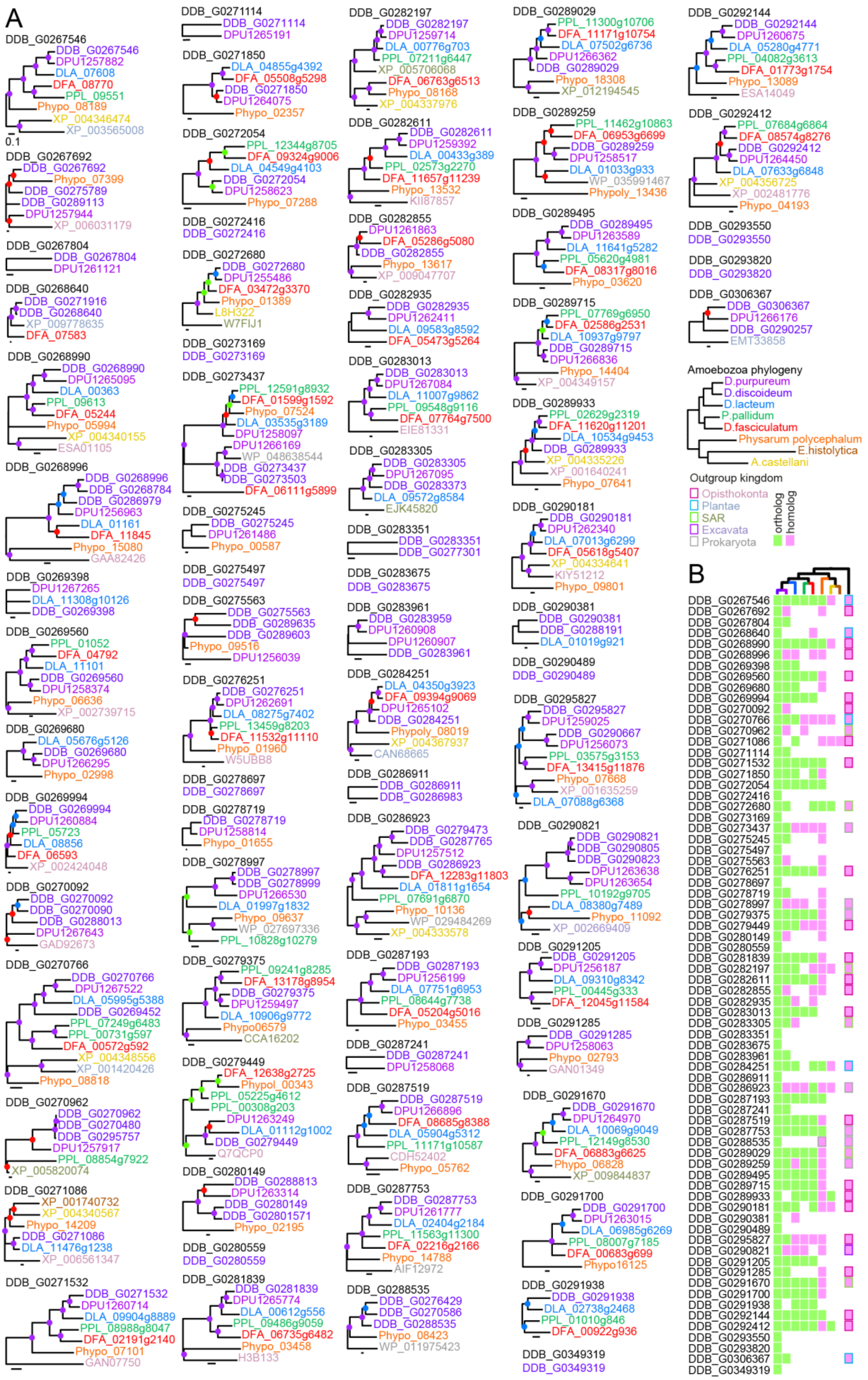
A
B
0.1
Amoebozoa phylogeny
D.purpureum
D.discoideum
D.lacteum
P.pallidum
D.fasciculatum
Physarum polycephalum
E.histolytica
A.castellani
Outgroup kingdom
Opisthokonta
Plantae
SAR
Excavata
Prokaryota
ortholog
homolog

Supplementary Figure 2. Gene conservation in genes without known function.
(**A**) Homologs of genes without known function. To assess to what extent genes without a proven biological role are conserved, we downloaded protein sequences for 72 *DD* genes from the Dictybase genome browser (http //dictybase.org) that were present at regular (0.4 Mb) intervals along each of the 6 chromosomes. We only selected genes without Demeric gene names (i.e. no known function) and yielding a protein > 200 amino acids, to minimize erroneously assigned coding sequences. If no gene with these criteria was present at the chosen location, the next 3' gene was selected. Homologs of all genes were identified by BlastP searches of 8 amoebozoan genomes and all non-redundant sequences in Genbank (see Methods). Protein sequences were aligned using ClustalOmega with 5 iterations. After deletion of regions that were not unambiguously aligned, alignments were subjected to Bayesian phylogenetic inference. The resulting trees are rooted at midpoint, with gene IDs and locus tags colour-coded to reflect the species or kingdom of origin.
(**B**) Gene conservation. The presence of orthologs or homologs of the 72 unnamed genes, as determined from the node structure and branch lengths of the protein trees shown in panel A, are plotted across a phylogeny of Amoebozoa and the outgroup that for each gene contained its closest homolog.

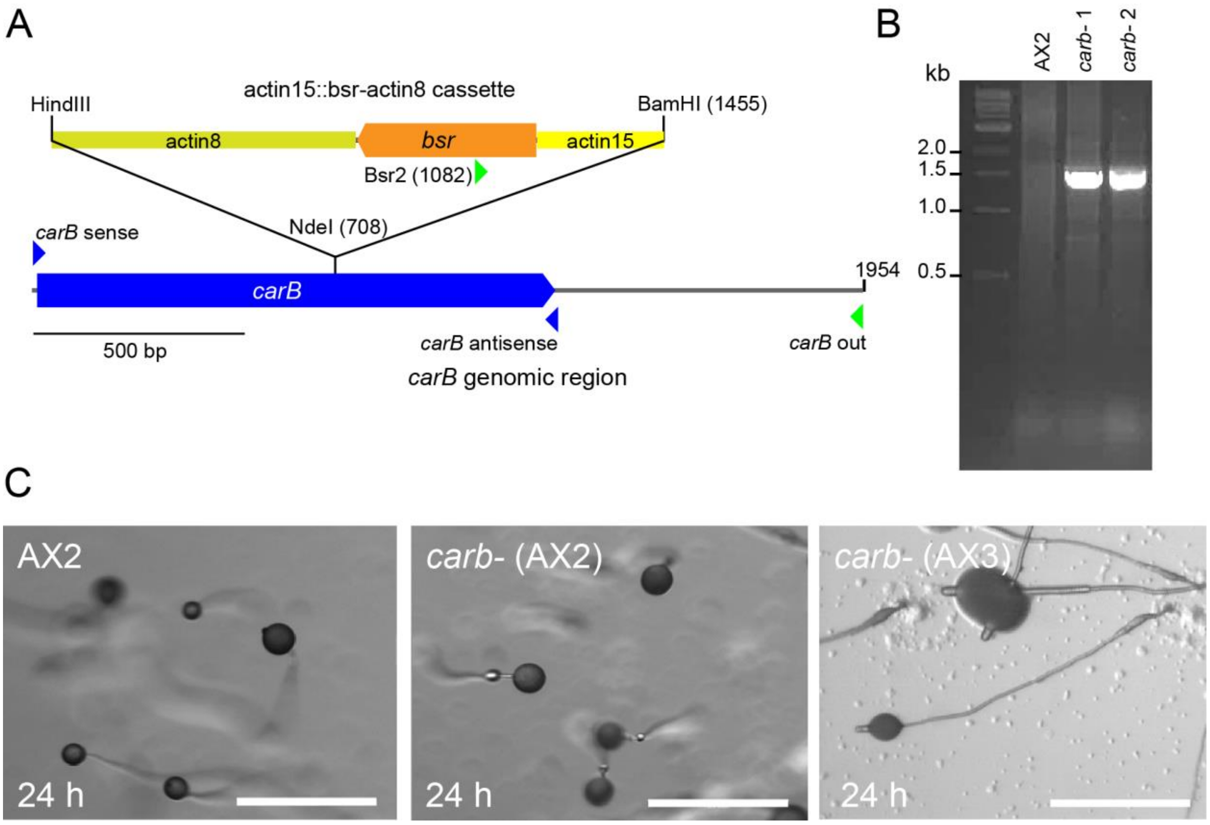

Supplementary Figure 3. *carB* gene knock-out.

(**A**). Knock-out construct. The actin15::Bsr selection-actin8 cassette was inserted into a *carB* fragment and the construct was introduced in *DD* AX2 and AX3 cells.

(**B**) Diagnosis. Genomic DNAs of blasticidin resistant clones were subjected to PCR using primers Bsr2 and carB-out, which should yield no amplification in AX2 or AX3 and a 1.6 kb products in *carb*- knockouts.

(**C**) Phenotype. The AX2 and AX3 *carb*- mutants developed normally into fruiting bodies. Scale bars:0.5 mm.

Supplementary Tables

Supplementary Table 1. *D.lacteum* genome sequencing statistics and genome features. The *D.lacteum* (*DL*) genome proved with a total size of 23.4 Mbp to be ~30% smaller than the *D.discoideum* (*DD*), *D.purpureum* (*DP*) *P.pallidum* (*PP*) and *D.fasciculatum* (*DF*) genomes[1-3]. The gene prediction package Augustus[4] predicted 10,232 protein coding genes, which is 1462 genes or 12.5% less than present in *PP* with a genome size of 32.9 Mbp. The average gene size was somewhat larger in *DL* than in the other genomes and the reduced number of genes is therefore not the only factor contributing to its smaller genome. Subtraction of the total length of DNA contained in coding sequences from the total genome length indicated a two-fold reduction of intergenic sequence in *DL*, which accounts for another 14% reduction in genome size compared to *PP*.

To investigate whether intron size and number were also reduced in *DL*, we compared these features in the set of manually curated developmentally essential orthologous genes, which were analysed for other purposes in this work. *DL* has the lowest number of introns per gene (1.3) and the lowest average size of introns (90 nt). Intron number (1.6) is not much larger in *DD*, but *DD* introns are 43% longer, while *PP* and *DF* have twice the number of introns of *DL* with a 10% greater length. The relatively small size of the *DL* genome therefore results from a combination of less genes, smaller gene family sizes, reduced intergenic DNA and reduced intron size and number. The average gene size of this set of developmentally essential genes is about twice as large as the average size of all genes in the genome, suggesting that small genes are less likely to have developmentally essential roles.

	DD	*DP*	*DL*	*PP*	*DF*
Contigs/supercontigs	226/6	1213/799	54/54	52/41	33/25
Total nucleotides (Mbp)	33.9	33.0	23.4	32.9	31.0
Average contig length (kbp)	155	27	432	320	1064
Nucleotide frequency (A/T%) overall/in CDS	77.6/72.6	75.5/ND	70.2/67.8	68/63.8	66.2/63.2
Palindrome arm size (kb)	45	ND	14	15	28
Mitochondrial genome size (kb)	55	ND	48	48	56
(Predicted) chromosome numbers	6	ND	~6	7	6
Repeat content	10%	4.4%	<1%	<1%	<1%
Telomere repeat structure	rDNA-like	ND	TAGGG	TAAGGG	TTAGGG
Centromeres	Large DIRS	ND	1900 nt AT-rich?	Small DIRS	Not iden-tifiable
Predicted tRNAs	401	353	57	273	198
Predicted coding sequences (CDS)	13,258	12,410	10,232	11,694	12,007
Average gene length	1604	1760	1712	1634	1696
Gene density (CDS per Mbp)	396	376	437	375	392
DNA in CDS (Mbp)	21.3	21.8	17.5	18.7	20.2
Intergenic DNA (Mbp)	12.6	11.2	5.8	14.2	10.8
Set of 100 orthologous genes:					
Average gene length (nt)	3272	3080	2880	2958	3106
Average protein length (aa)	1024	937	923	900	948
Introns per gene	1.6	2.4	1.3	2.7	2.8
Average intron length (nt)	129	112	90	99	99

ND: not determined

Supplementary Table 2. Conservation of *DD* DEG and unnamed genes.
(**A**) Using the data in Supplementary Data 2, percentages of *DD* DEG were determined which had orthologs (o), orthologs/homologs with conserved domains (hd) or conserved expression profiles (he) over 5, 4, 3, 2 or 1 Dictyostelid genomes. This was done for all DEG, and separately for DEG categorized according to the stage where the null mutant phenotype was first evident. Using the data in Supplementary Fig. 2, percentages of orthologs and homologs of unnamed genes with no known function were also determined over 5 to 1 Dictyostelid genomes.
(**B**) Percentages of homologs/orthologs (h) and homologs/orthologs with conserved domains (hd) of *DD* genes were determined separately over each of the three amoebozoan genomes and the non-amoebozoan outgroup. n = total number of genes in each category.

A		% of conserved genes across 5 Dictyostelid genomes														
		5			4			3			2			1 (*DD*)		
dev.essential	n	o	hd	he	o	hd	he	o	hd	he	o	hd	he	o	hd	he
any stage	385	73	54	45	9	11	23	5	10	16	8	11	8	6	14	6
growth	66	83	62	50	6	18	18	5	6	18	5	5	9	2	9	5
aggregation	124	73	54	44	10	9	27	5	12	18	7	11	6	6	14	6
mound	39	74	56	54	10	15	21	3	13	13	3	3	5	10	13	8
slug	21	76	48	52	5	14	29	5	10	19	10	14	0	5	14	0
culmination	42	67	52	43	14	7	29	2	10	14	5	10	2	12	21	12
(pre)stalk	22	68	64	36	9	9	23	5	5	18	18	18	18	0	5	5
(pre)spore	71	68	46	42	8	10	18	7	11	14	11	17	11	6	15	4
unnamed		o	h		o	h		o	h		o	h		o	h	
	72	31	39		8	14		8	8		19	21		33	18	

B		% of conserved genes in unicellular Amoebozoa and the outgroup							
		PhyP		*AC*		*EH*		outgroup	
dev.essential	n	h	hd	h	hd	h	hd	h	hd
any stage	385	76	49	46	29	19	10	71	42
growth	66	85	56	52	36	18	9	79	53
aggregation	124	73	44	39	23	12	6	65	37
mound	39	74	51	51	28	28	21	77	51
slug	21	95	48	38	10	10	0	81	29
culmination	42	71	43	33	26	5	0	60	36
(pre)stalk	22	73	50	45	36	50	32	55	45
(pre)spore	71	70	51	62	35	30	17	80	41
unnamed	72	63		15		1		57	

Supplementary References

1 Eichinger, L. *et al.* The genome of the social amoeba *Dictyostelium discoideum*. *Nature* **435**, 43-57 (2005).
2 Heidel, A. *et al.* Phylogeny-wide analysis of social amoeba genomes highlights ancient origins for complex intercellular communication. *Genome Res.*, 1882-1891, doi:10.1101/gr.121137.111 (2011).
3 Sucgang, R. *et al.* Comparative genomics of the social amoebae *Dictyostelium discoideum* and *Dictyostelium purpureum*. *Genome Biol.* **12**, R20, doi:10.1186/gb-2011-12-2-r20 (2011).

4 Stanke, M. & Morgenstern, B. AUGUSTUS: a web server for gene prediction in eukaryotes that allows user-defined constraints. *Nucleic Acids Res* **33**, W465-467, doi:10.1093/nar/gki458 (2005).
