# Supplementary material for: The multicellularity genes of dictyostelid social amoebas
Source: Nat Commun. 2016 Jun 30;7:12085. doi: 10.1038/ncomms12085 (PMC4931340; doi:10.1038/ncomms12085)
Supplement: Supplementary Data 2 — Phylogenies of D. discoideum developmentally essential genes and their homologs, annotated with domain architectures and developmental expression profiles [file ncomms12085-s3.pdf]

Supplementary Data File 2. Phylogenies of developmentally essential genes annotated with domain architectures and developmental expression profiles

Phylogenies of 385 *DD* DEG with their closest homologs in the Dictyostelids *DP*, *DL*, *PP* and *DF*, the unicellular amoebozoa *PhyP*, *AC* and *EH* and outside of Amoebozoa were prepared by Bayesian inference as outlined in Methods. Bayesian posterior probabilities of tree nodes are indicated by coloured dots. The trees are annotated with the protein domain architectures of the genes as determined by SMART[1], using simplified diagrams to allow size-reduction. PFAM[2] or SCOP[3] domains are represented by rectangles, filled with colour gradients starting from black or tan on the left side, respectively.

Developmental transcriptional profiles were retrieved for *DD* (DDBa, DDBb) and *DP* (DPUa, DPUb) from a replicate time course[4] and for *DF* and *PP* from two separate experiments, which were analysed using either the Roche 454 (DFAa, PPLa) or Illumina platforms (DFAb, PPLb). For *DL* a single experiment, analysed using the Illumina platform (DLA) was used. Due to differences in the duration of development between species, the profiles were stage-matched rather than time matched. These stage are vegetative (t0 for *DD*, *DP*), early aggregate (t4), completed aggregate (t8), tipped mound (t12), slug (t16), early culmination (t20), mature fruiting body (t24). The *DD* and *DP* experiments also contain transcript reads for purified prestalk (pst) and prespore cells (psp), while the *PP* 454 series contains reads from purified spore- and stalk cells.

For time series, normalized reads were expressed as percentage of the highest read count of the series and presented as yellow to red heatmaps. For (pre)stalk and (pre)spore isolates reads are expressed as percentage of the summed reads of both isolates, and presented as white to green heatmaps. Names of species or kingdoms (for non-Amoebozoa) are colour-coded, as indicated at the top of the figure. Genes are alphabetically ordered and can be found at the following page numbers:

**Gene name**	**page**	**Gene name**	**page**
aa-al	1	grp-ip	12
am-bz	2	kc-mhc	13
ca-cm	3	mhk-myoB	14
cn-co	4	myoG-pad	15
cp-cy	5	pak-pkg	16
dga-DG1039	6	pl-pte	17
DG1040-DG1122	7	ptp-ri	18
DG2033-dn	8	ro-snf	19
do-fr p9 fu-gefB	10	snp-sv	20
gefL-grl	11	ta-tsg	21
		tsu-zi	22

References

1 Schultz, J., Milpetz, F., Bork, P. & Ponting, C. P. SMART, a simple modular architecture research tool: identification of signaling domains. *Proc. Natl. Acad. Sci. USA* **95**, 5857-5864. (1998).

2 Bateman, A. *et al.* The Pfam protein families database. *Nucleic Acids Res.* **28**, 263-266. (2000).

3 Murzin, A. G., Brenner, S. E., Hubbard, T. & Chothia, C. SCOP: a structural classification of proteins database for the investigation of sequences and structures. *J Mol Biol* **247**, 536-540 (1995).

4 Parikh, A. *et al.* Conserved developmental transcriptomes in evolutionarily divergent species. *Genome Biol* **11**, R35, doi:gb-2010-11-3-r35 (2010).

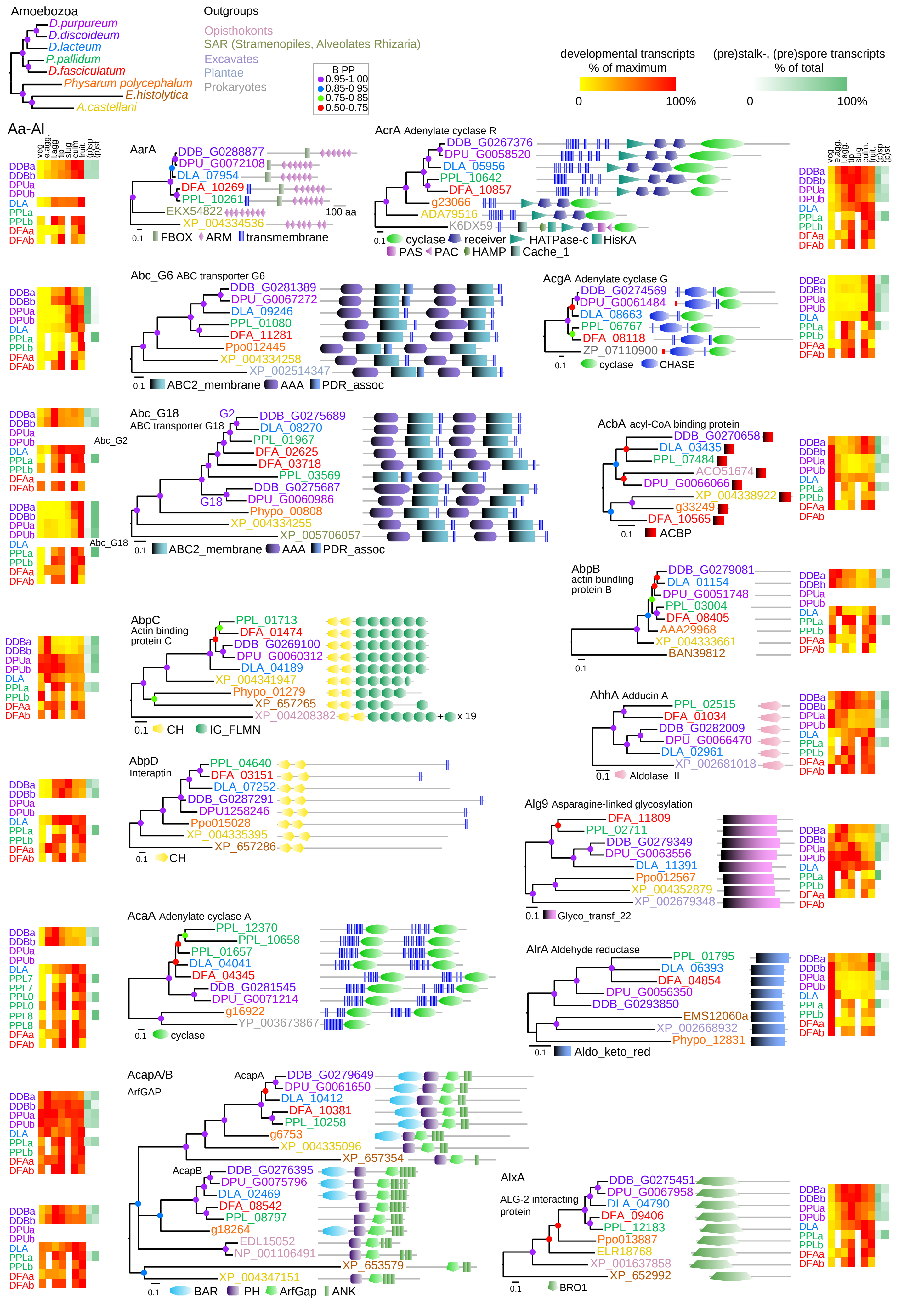

Amoebozoa
D.purpureum
D.discoideum
D.lacteum
P.pallidum
D.fasciculatum
Physarum polycephalum
E.histolytica
A.castellani
Outgroups
Opisthokonts
SAR (Stramenopiles, Alveolates Rhizaria)
Excavates
Plantae
Prokaryotes
B PP
0.95-1 00
0.85-0 95
0.75-0 85
0.50-0.75
developmental transcripts % of maximum
0
100%
(pre)stalk-, (pre)spore transcripts % of total
0
100%
Aa-Al
veg e.agg. l.agg. tip slug culm. fruit. (p)sp (p)st
DDBa DDBb DPUa DPUb DLA PPLa PPLb DFAa DFAb
AarA
DDB_G0288877
DPU_G0072108
DLA_07954
DFA_10269
PPL_10261
EKX54822
XP_004334536
100 aa
0.1
FBOX
ARM
transmembrane
AcrA Adenylate cyclase R
DDB_G0267376
DPU_G0058520
DLA_05956
PPL_10642
DFA_10857
g23066
ADA79516
K6DX59
cyclase
receiver
HATPase-c
HisKA
PAS
PAC
HAMP
Cache_1
Abc_G6 ABC transporter G6
DDB_G0281389
DPU_G0067272
DLA_09246
PPL_01080
DFA_11281
Ppo012445
XP_004334258
XP_002514347
ABC2_membrane
AAA
PDR_assoc
AcgA Adenylate cyclase G
DDB_G0274569
DPU_G0061484
DLA_08663
PPL_06767
DFA_08118
ZP_07110900
cyclase
CHASE
Abc_G18
ABC transporter G18
G2
DDB_G0275689
DLA_08270
PPL_01967
DFA_02625
DFA_03718
PPL_03569
DDB_G0275687
G18
DPU_G0060986
Phypo_00808
XP_004334255
XP_005706057
Abc_G2
Abc_G18
ABC2_membrane
AAA
PDR_assoc
AcbA acyl-CoA binding protein
DDB_G0270658
DLA_03435
PPL_07484
ACO51674
DPU_G0066066
XP_004338922
g33249
DFA_10565
ACBP
AbpB actin bundling protein B
DDB_G0279081
DLA_01154
DPU_G0051748
PPL_03004
DFA_08405
AAA29968
XP_004333661
BAN39812
AbpC Actin binding protein C
PPL_01713
DFA_01474
DDB_G0269100
DPU_G0060312
DLA_04189
XP_004341947
Phypo_01279
XP_657265
XP_004208382
+ x 19
CH
IG_FLMN
AhhA Adducin A
PPL_02515
DFA_01034
DDB_G0282009
DPU_G0066470
DLA_02961
XP_002681018
Aldolase_II
AbpD Interaptin
PPL_04640
DFA_03151
DLA_07252
DDB_G0287291
DPU1258246
Ppo015028
XP_004335395
XP_657286
CH
Alg9 Asparagine-linked glycosylation
DFA_11809
PPL_02711
DDB_G0279349
DPU_G0063556
DLA_11391
Ppo012567
XP_004352879
XP_002679348
Glyco_transf_22
AcaA Adenylate cyclase A
PPL_12370
PPL_10658
PPL_01657
DLA_04041
DFA_04345
DDB_G0281545
DPU_G0071214
g16922
YP_003673867
cyclase
PPL7 PPL7 PPL0 PPL0 PPL8 PPL8
AlrA Aldehyde reductase
PPL_01795
DLA_06393
DFA_04854
DPU_G0056350
DDB_G0293850
EMS12060a
XP_002668932
Phypo_12831
Aldo_keto_red
AcapA/B
ArfGAP
AcapA
DDB_G0279649
DPU_G0061650
DLA_10412
DFA_10381
PPL_10258
g6753
XP_004335096
XP_657354
AcapB
DDB_G0276395
DPU_G0075796
DLA_02469
DFA_08542
PPL_08797
g18264
EDL15052
NP_001106491
XP_653579
XP_004347151
BAR
PH
ArfGap
ANK
AlxA
ALG-2 interacting protein
DDB_G0275451
DPU_G0067958
DLA_04790
DFA_09406
PPL_12183
Ppo013887
ELR18768
XP_001637858
XP_652992
BRO1

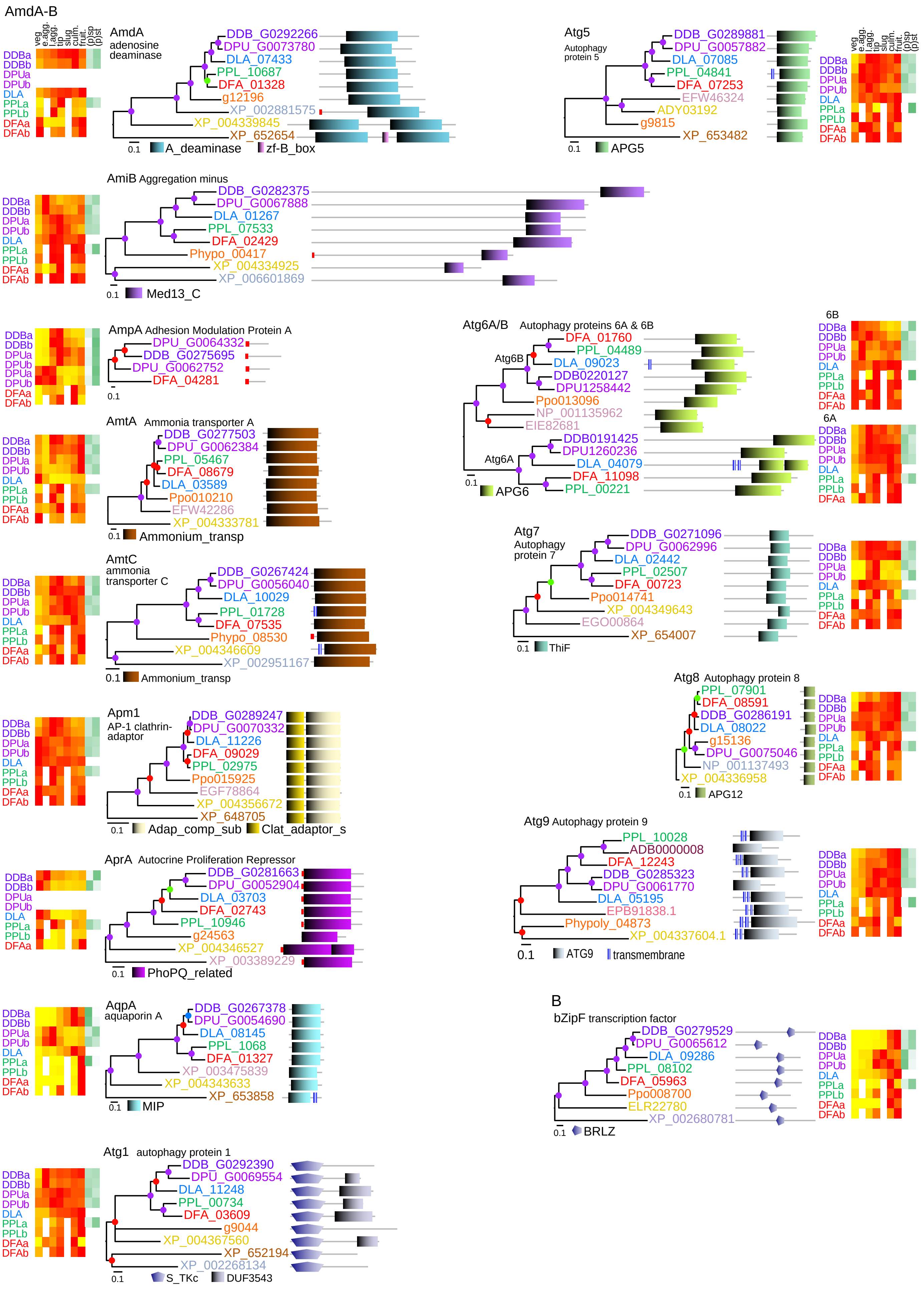
AmdA-B
veg
e.agg.
l.agg.
tip
slug
culm.
fruit.
(p)sp
(p)st
DDBa
DDBb
DPUa
DPUb
DLA
PPLa
PPLb
DFAa
DFAb
AmdA
adenosine deaminase
DDB_G0292266
DPU_G0073780
DLA_07433
PPL_10687
DFA_01328
g12196
XP_002881575
XP_004339845
XP_652654
0.1
A_deaminase
zf-B_box
Atg5
Autophagy protein 5
DDB_G0289881
DPU_G0057882
DLA_07085
PPL_04841
DFA_07253
EFW46324
ADY03192
g9815
XP_653482
APG5
AmiB Aggregation minus
DDB_G0282375
DPU_G0067888
DLA_01267
PPL_07533
DFA_02429
Phypo_00417
XP_004334925
XP_006601869
Med13_C
AmpA Adhesion Modulation Protein A
DPU_G0064332
DDB_G0275695
DPU_G0062752
DFA_04281
AmtA Ammonia transporter A
DDB_G0277503
DPU_G0062384
PPL_05467
DFA_08679
DLA_03589
Ppo010210
EFW42286
XP_004333781
Ammonium_transp
AmtC
ammonia transporter C
DDB_G0267424
DPU_G0056040
DLA_10029
PPL_01728
DFA_07535
Phypo_08530
XP_004346609
XP_002951167
Apm1
AP-1 clathrin-adaptor
DDB_G0289247
DPU_G0070332
DLA_11226
DFA_09029
PPL_02975
Ppo015925
EGF78864
XP_004356672
XP_648705
Adap_comp_sub
Clat_adaptor_s
AprA Autocrine Proliferation Repressor
DDB_G0281663
DPU_G0052904
DLA_03703
DFA_02743
PPL_10946
g24563
XP_004346527
XP_003389229
PhoPQ_related
AqpA
aquaporin A
DDB_G0267378
DPU_G0054690
DLA_08145
PPL_1068
DFA_01327
XP_003475839
XP_004343633
XP_653858
MIP
Atg1 autophagy protein 1
DDB_G0292390
DPU_G0069554
DLA_11248
PPL_00734
DFA_03609
g9044
XP_004367560
XP_652194
XP_002268134
S_TKc
DUF3543
Atg6A/B Autophagy proteins 6A & 6B
6B
6A
Atg6B
Atg6A
DFA_01760
PPL_04489
DLA_09023
DDB0220127
DPU1258442
Ppo013096
NP_001135962
EIE82681
DDB0191425
DPU1260236
DLA_04079
DFA_11098
PPL_00221
APG6
Atg7
Autophagy protein 7
DDB_G0271096
DPU_G0062996
DLA_02442
PPL_02507
DFA_00723
Ppo014741
XP_004349643
EGO00864
XP_654007
ThiF
Atg8 Autophagy protein 8
PPL_07901
DFA_08591
DDB_G0286191
DLA_08022
g15136
DPU_G0075046
NP_001137493
XP_004336958
APG12
Atg9 Autophagy protein 9
PPL_10028
ADB0000008
DFA_12243
DDB_G0285323
DPU_G0061770
DLA_05195
EPB91838.1
Phypoly_04873
XP_004337604.1
ATG9
transmembrane
B
bZipF transcription factor
DDB_G0279529
DPU_G0065612
DLA_09286
PPL_08102
DFA_05963
Ppo008700
ELR22780
XP_002680781
BRLZ

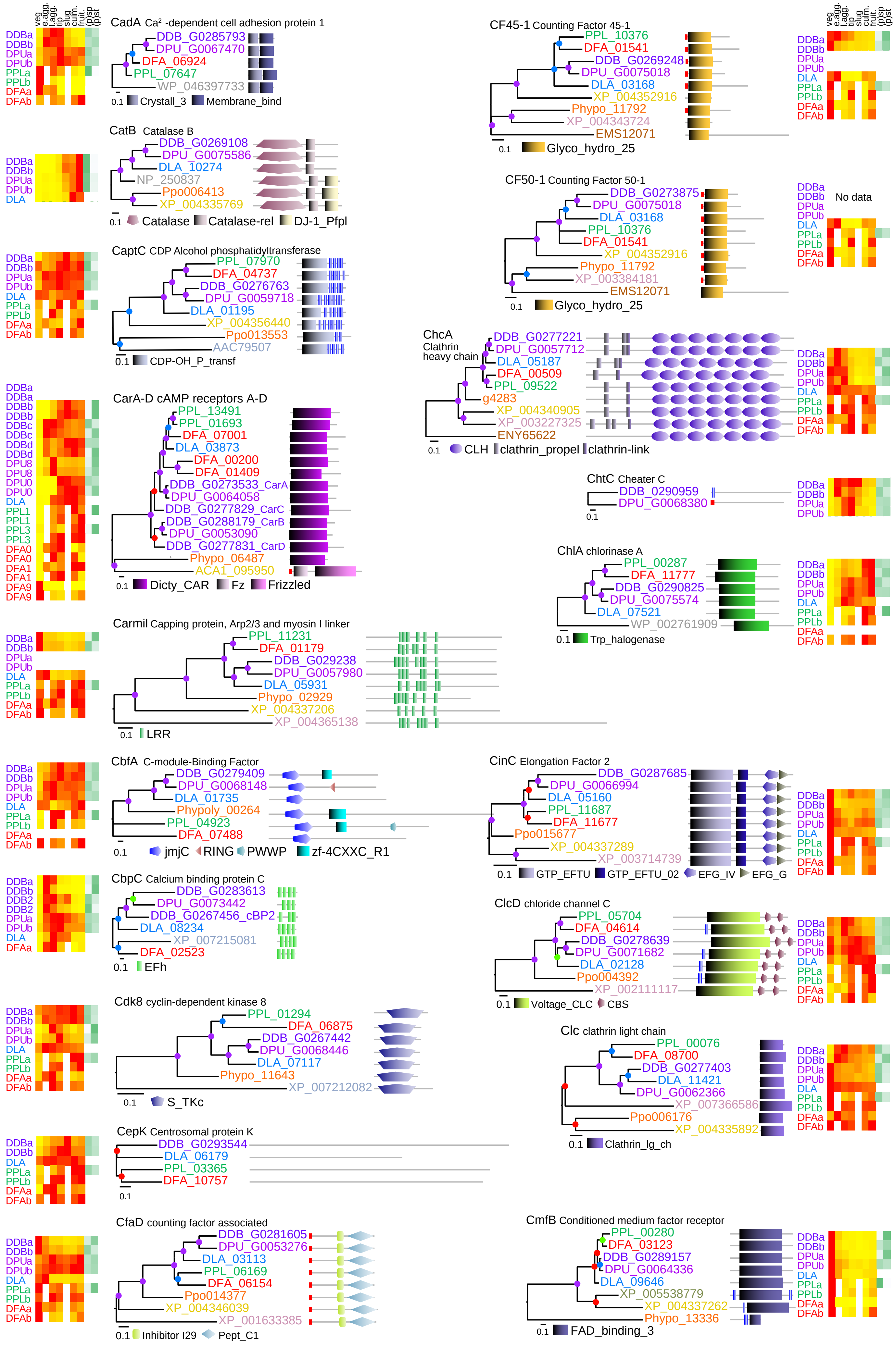
veg
e.agg.
l.agg.
tip
slug
culm.
fruit
(p)sp
(p)st
CadA Ca² -dependent cell adhesion protein 1
DDB_G0285793
DPU_G0067470
DFA_06924
PPL_07647
WP_046397733
0.1 Crystall_3 Membrane_bind
CatB Catalase B
DDB_G0269108
DPU_G0075586
DLA_10274
NP_250837
Ppo006413
XP_004335769
0.1 Catalase Catalase-rel DJ-1_Pfpl
CaptC CDP Alcohol phosphatidyltransferase
PPL_07970
DFA_04737
DDB_G0276763
DPU_G0059718
DLA_01195
XP_004356440
Ppo013553
AAC79507
0.1 CDP-OH_P_transf
CarA-D cAMP receptors A-D
PPL_13491
PPL_01693
DFA_07001
DLA_03873
DFA_00200
DFA_01409
DDB_G0273533_CarA
DPU_G0064058
DDB_G0277829_CarC
DDB_G0288179_CarB
DPU_G0053090
DDB_G0277831_CarD
Phypo_06487
ACA1_095950
0.1 Dicty_CAR Fz Frizzled
Carmil Capping protein, Arp2/3 and myosin I linker
PPL_11231
DFA_01179
DDB_G029238
DPU_G0057980
DLA_05931
Phypo_02929
XP_004337206
XP_004365138
0.1 LRR
CbfA C-module-Binding Factor
DDB_G0279409
DPU_G0068148
DLA_01735
Phypoly_00264
PPL_04923
DFA_07488
0.1 jmjC RING PWWP zf-4CXXC_R1
CbpC Calcium binding protein C
DDB_G0283613
DPU_G0073442
DDB_G0267456_cBP2
DLA_08234
XP_007215081
DFA_02523
0.1 EFh
Cdk8 cyclin-dependent kinase 8
PPL_01294
DFA_06875
DDB_G0267442
DPU_G0068446
DLA_07117
Phypo_11643
XP_007212082
0.1 S_TKc
CepK Centrosomal protein K
DDB_G0293544
DLA_06179
PPL_03365
DFA_10757
0.1
CfaD counting factor associated
DDB_G0281605
DPU_G0053276
DLA_03113
PPL_06169
DFA_06154
Ppo014377
XP_004346039
XP_001633385
0.1 Inhibitor I29 Pept_C1
CF45-1 Counting Factor 45-1
PPL_10376
DFA_01541
DDB_G0269248
DPU_G0075018
DLA_03168
XP_004352916
Phypo_11792
XP_004343724
EMS12071
0.1 Glyco_hydro_25
CF50-1 Counting Factor 50-1
DDB_G0273875
DPU_G0075018
DLA_03168
PPL_10376
DFA_01541
XP_004352916
Phypo_11792
XP_003384181
EMS12071
0.1 Glyco_hydro_25
No data
ChcA Clathrin heavy chain
DDB_G0277221
DPU_G0057712
DLA_05187
DFA_00509
PPL_09522
g4283
XP_004340905
XP_003227325
ENY65622
0.1 CLH clathrin_propel clathrin-link
ChtC Cheater C
DDB_0290959
DPU_G0068380
0.1
ChlA chlorinase A
PPL_00287
DFA_11777
DDB_G0290825
DPU_G0075574
DLA_07521
WP_002761909
0.1 Trp_halogenase
CinC Elongation Factor 2
DDB_G0287685
DPU_G0066994
DLA_05160
PPL_11687
DFA_11677
Ppo015677
XP_004337289
XP_003714739
0.1 GTP_EFTU GTP_EFTU_02 EFG_IV EFG_G
ClcD chloride channel C
PPL_05704
DFA_04614
DDB_G0278639
DPU_G0071682
DLA_02128
Ppo004392
XP_002111117
0.1 Voltage_CLC CBS
Clc clathrin light chain
PPL_00076
DFA_08700
DDB_G0277403
DLA_11421
DPU_G0062366
XP_007366586
Ppo006176
XP_004335892
0.1 Clathrin_lg_ch
CmfB Conditioned medium factor receptor
PPL_00280
DFA_03123
DDB_G0289157
DPU_G0064336
DLA_09646
XP_005538779
XP_004337262
Phypo_13336
0.1 FAD_binding_3
DDBa
DDBb
DPUa
DPUb
DLA
PPLa
PPLb
DFAa
DFAb

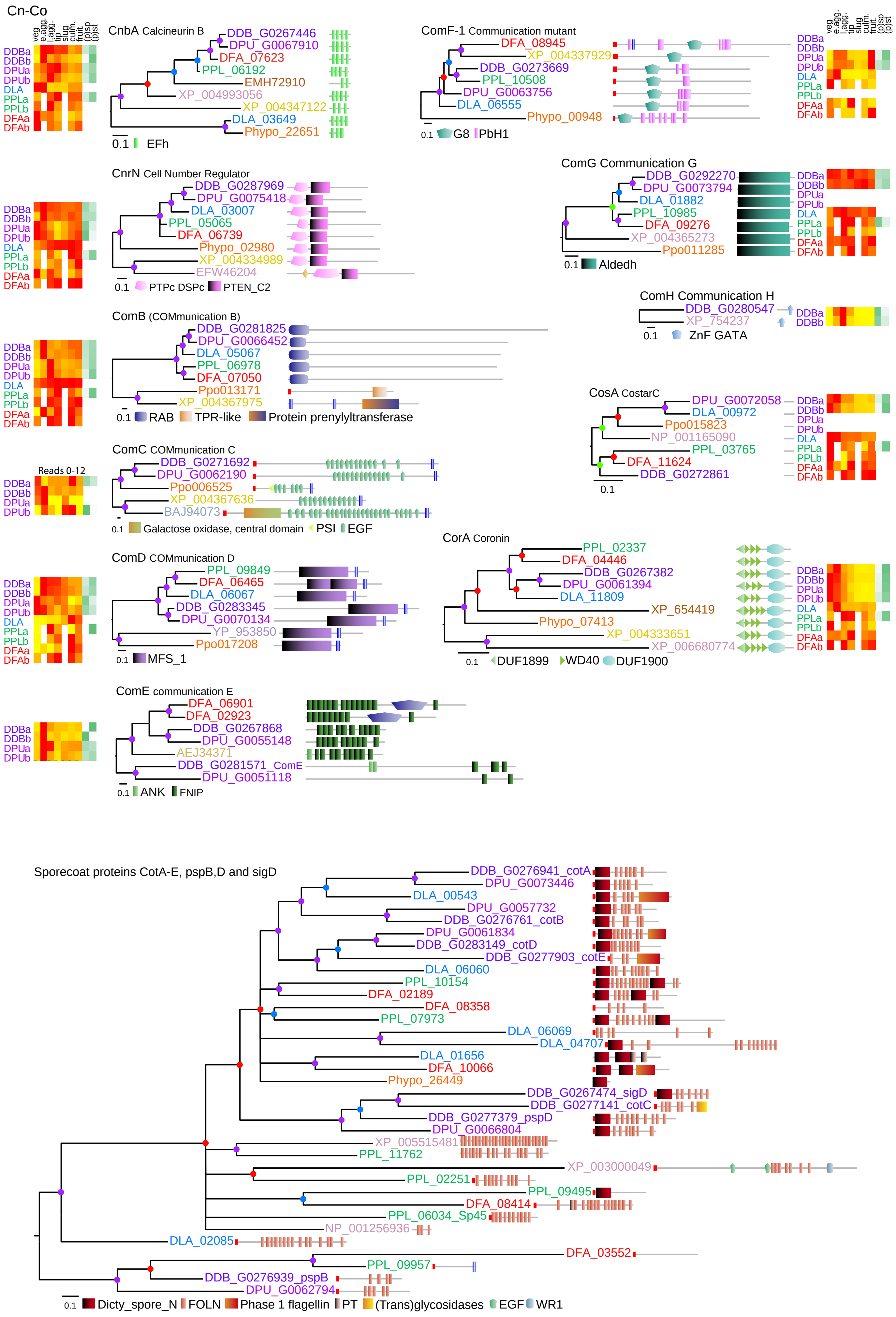
Cn-Co
veg
e.agg.
l.agg.
tip
slug
culm.
fruit.
(p)sp
(p)st
DDBa
DDBb
DPUa
DPUb
DLA
PPLa
PPLb
DFAa
DFAb
CnbA Calcineurin B
DDB_G0267446
DPU_G0067910
DFA_07623
PPL_06192
EMH72910
XP_004993056
XP_004347122
DLA_03649
Phypo_22651
0.1
EFh
ComF-1 Communication mutant
DFA_08945
XP_004337929
DDB_G0273669
PPL_10508
DPU_G0063756
DLA_06555
Phypo_00948
G8
PbH1
CnrN Cell Number Regulator
DDB_G0287969
DPU_G0075418
DLA_03007
PPL_05065
DFA_06739
Phypo_02980
XP_004334989
EFW46204
PTPc DSPc
PTEN_C2
ComG Communication G
DDB_G0292270
DPU_G0073794
DLA_01882
PPL_10985
DFA_09276
XP_004365273
Ppo011285
Aldedh
ComH Communication H
DDB_G0280547
XP_754237
ZnF GATA
ComB (COMmunication B)
DDB_G0281825
DPU_G0066452
DLA_05067
PPL_06978
DFA_07050
Ppo013171
XP_004367975
RAB
TPR-like
Protein prenylyltransferase
CosA CostarC
DPU_G0072058
DLA_00972
Ppo015823
NP_001165090
PPL_03765
DFA_11624
DDB_G0272861
ComC COMmunication C
Reads 0-12
DDB_G0271692
DPU_G0062190
Ppo006525
XP_004367636
BAJ94073
Galactose oxidase, central domain
PSI
EGF
CorA Coronin
PPL_02337
DFA_04446
DDB_G0267382
DPU_G0061394
DLA_11809
XP_654419
Phypo_07413
XP_004333651
XP_006680774
DUF1899
WD40
DUF1900
ComD COMmunication D
PPL_09849
DFA_06465
DLA_06067
DDB_G0283345
DPU_G0070134
YP_953850
Ppo017208
MFS_1
ComE communication E
DFA_06901
DFA_02923
DDB_G0267868
DPU_G0055148
AEJ34371
DDB_G0281571_ComE
DPU_G0051118
ANK
FNIP
Sporecoat proteins CotA-E, pspB,D and sigD
DDB_G0276941_cotA
DPU_G0073446
DLA_00543
DPU_G0057732
DDB_G0276761_cotB
DPU_G0061834
DDB_G0283149_cotD
DDB_G0277903_cotE
DLA_06060
PPL_10154
DFA_02189
DFA_08358
PPL_07973
DLA_06069
DLA_04707
DLA_01656
DFA_10066
Phypo_26449
DDB_G0267474_sigD
DDB_G0277141_cotC
DDB_G0277379_pspD
DPU_G0066804
XP_005515481
PPL_11762
XP_003000049
PPL_02251
PPL_09495
DFA_08414
PPL_06034_Sp45
NP_001256936
DLA_02085
DFA_03552
PPL_09957
DDB_G0276939_pspB
DPU_G0062794
Dicty_spore_N
FOLN
Phase 1 flagellin
PT
(Trans)glycosidases
EGF
WR1

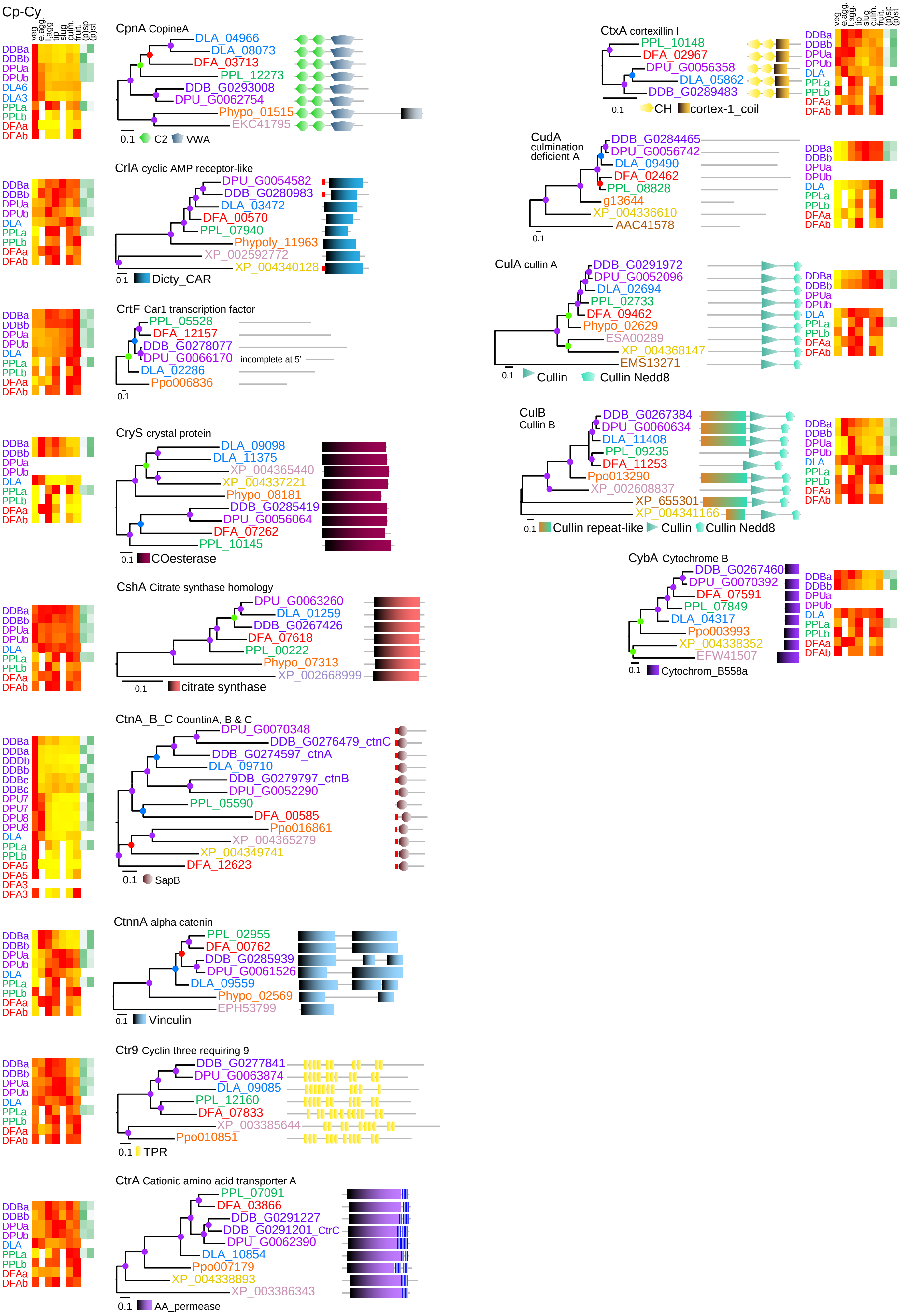
Cp-Cy
veg
e.agg.
l.agg.
tip
slug
culm.
fruit.
(p)sp
(p)st
CpnA CopineA
DLA_04966
DLA_08073
DFA_03713
PPL_12273
DDB_G0293008
DPU_G0062754
Phypo_01515
EKC41795
0.1 C2 VWA
CrlA cyclic AMP receptor-like
DPU_G0054582
DDB_G0280983
DLA_03472
DFA_00570
PPL_07940
Phypoly_11963
XP_002592772
XP_004340128
0.1 Dicty_CAR
CrtF Car1 transcription factor
PPL_05528
DFA_12157
DDB_G0278077
DPU_G0066170 incomplete at 5'
DLA_02286
Ppo006836
0.1
CryS crystal protein
DLA_09098
DLA_11375
XP_004365440
XP_004337221
Phypo_08181
DDB_G0285419
DPU_G0056064
DFA_07262
PPL_10145
0.1 COesterase
CshA Citrate synthase homology
DPU_G0063260
DLA_01259
DDB_G0267426
DFA_07618
PPL_00222
Phypo_07313
XP_002668999
0.1 citrate synthase
CtnA_B_C CountinA, B & C
DPU_G0070348
DDB_G0276479_ctnC
DDB_G0274597_ctnA
DLA_09710
DDB_G0279797_ctnB
DPU_G0052290
PPL_05590
DFA_00585
Ppo016861
XP_004365279
XP_004349741
DFA_12623
0.1 SapB
DDBa DDBa DDDb DDBb DDBc DDBc DPU7 DPU7 DPU8 DPU8 DLA PPLa PPLb DFA5 DFA5 DFA3 DFA3
CtnnA alpha catenin
PPL_02955
DFA_00762
DDB_G0285939
DPU_G0061526
DLA_09559
Phypo_02569
EPH53799
0.1 Vinculin
Ctr9 Cyclin three requiring 9
DDB_G0277841
DPU_G0063874
DLA_09085
PPL_12160
DFA_07833
XP_003385644
Ppo010851
0.1 TPR
CtrA Cationic amino acid transporter A
PPL_07091
DFA_03866
DDB_G0291227
DDB_G0291201_CtrC
DPU_G0062390
DLA_10854
Ppo007179
XP_004338893
XP_003386343
0.1 AA_permease
CtxA cortexillin I
PPL_10148
DFA_02967
DPU_G0056358
DLA_05862
DDB_G0289483
0.1 CH cortex-1_coil
CudA culmination deficient A
DDB_G0284465
DPU_G0056742
DLA_09490
DFA_02462
PPL_08828
g13644
XP_004336610
AAC41578
0.1
CulA cullin A
DDB_G0291972
DPU_G0052096
DLA_02694
PPL_02733
DFA_09462
Phypo_02629
ESA00289
XP_004368147
EMS13271
0.1 Cullin Cullin Nedd8
CulB Cullin B
DDB_G0267384
DPU_G0060634
DLA_11408
PPL_09235
DFA_11253
Ppo013290
XP_002608837
XP_655301
XP_004341166
0.1 Cullin repeat-like Cullin Cullin Nedd8
CybA Cytochrome B
DDB_G0267460
DPU_G0070392
DFA_07591
PPL_07849
DLA_04317
Ppo003993
XP_004338352
EFW41507
0.1 Cytochrom_B558a
DDBa DDBb DPUa DPUb DLA PPLa PPLb DFAa DFAb

Da-DG1039

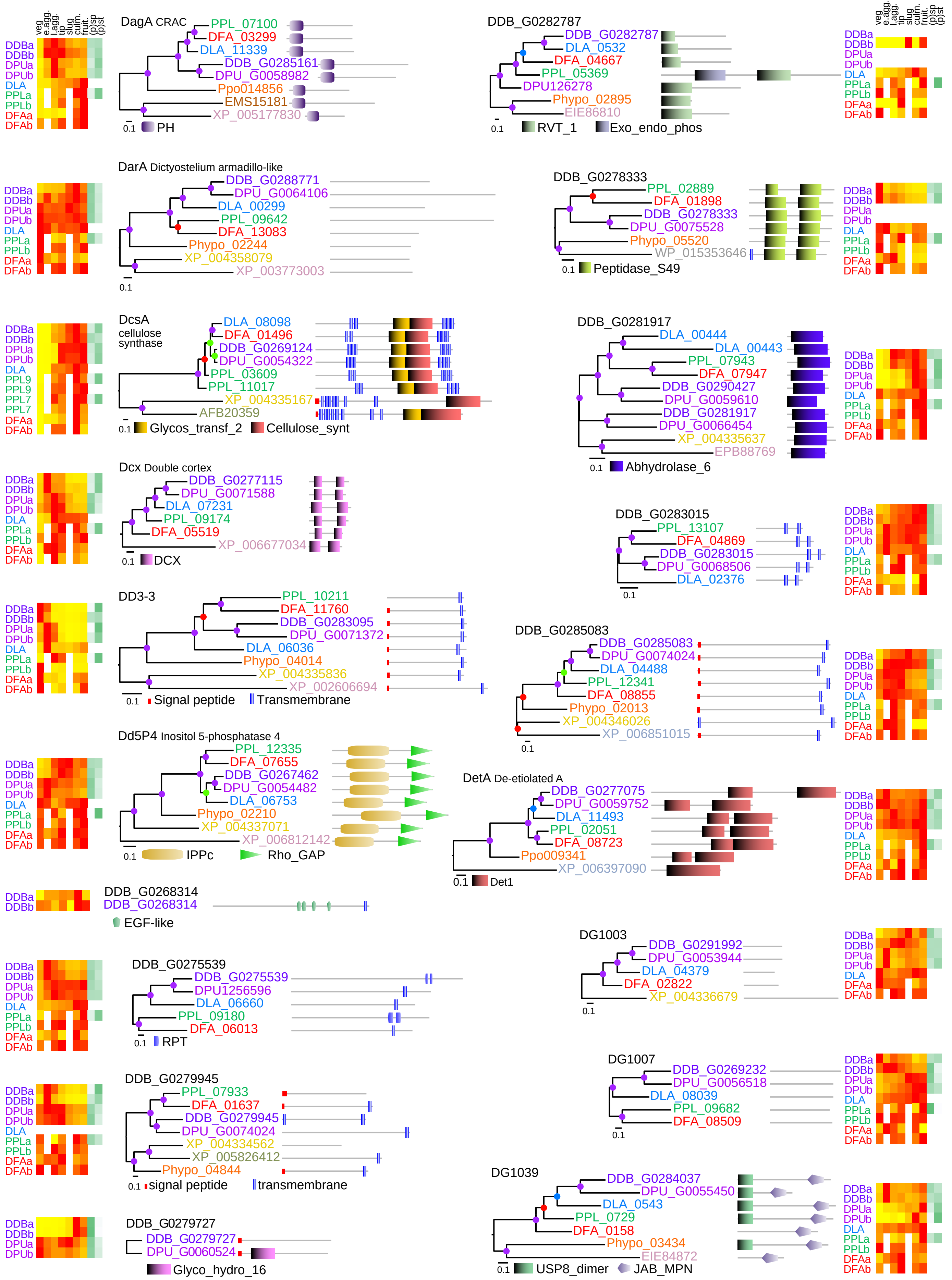
veg
e.agg.
l.agg.
tip
slug
culm.
fruit.
(p)sp
(p)st
DDBa
DDBb
DPUa
DPUb
DLA
PPLa
PPLb
DFAa
DFAb
DagA CRAC
PPL_07100
DFA_03299
DLA_11339
DDB_G0285161
DPU_G0058982
Ppo014856
EMS15181
XP_005177830
0.1
PH
DDB_G0282787
DDB_G0282787
DLA_0532
DFA_04667
PPL_05369
DPU126278
Phypo_02895
EIE86810
0.1
RVT_1
Exo_endo_phos
DarA Dictyostelium armadillo-like
DDB_G0288771
DPU_G0064106
DLA_00299
PPL_09642
DFA_13083
Phypo_02244
XP_004358079
XP_003773003
0.1
DDB_G0278333
PPL_02889
DFA_01898
DDB_G0278333
DPU_G0075528
Phypo_05520
WP_015353646
0.1
Peptidase_S49
DcsA cellulose synthase
DLA_08098
DFA_01496
DDB_G0269124
DPU_G0054322
PPL_03609
PPL_11017
XP_004335167
AFB20359
0.1
Glycos_transf_2
Cellulose_synt
PPL9
PPL7
DDB_G0281917
DLA_00444
DLA_00443
PPL_07943
DFA_07947
DDB_G0290427
DPU_G0059610
DDB_G0281917
DPU_G0066454
XP_004335637
EPB88769
0.1
Abhydrolase_6
Dcx Double cortex
DDB_G0277115
DPU_G0071588
DLA_07231
PPL_09174
DFA_05519
XP_006677034
0.1
DCX
DDB_G0283015
PPL_13107
DFA_04869
DDB_G0283015
DPU_G0068506
DLA_02376
0.1
DD3-3
PPL_10211
DFA_11760
DDB_G0283095
DPU_G0071372
DLA_06036
Phypo_04014
XP_004335836
XP_002606694
0.1
Signal peptide
Transmembrane
DDB_G0285083
DDB_G0285083
DPU_G0074024
DLA_04488
PPL_12341
DFA_08855
Phypo_02013
XP_004346026
XP_006851015
0.1
Dd5P4 Inositol 5-phosphatase 4
PPL_12335
DFA_07655
DDB_G0267462
DPU_G0054482
DLA_06753
Phypo_02210
XP_004337071
XP_006812142
0.1
IPPc
Rho_GAP
DetA De-etiolated A
DDB_G0277075
DPU_G0059752
DLA_11493
PPL_02051
DFA_08723
Ppo009341
XP_006397090
0.1
Det1
DDB_G0268314
DDB_G0268314
EGF-like
DG1003
DDB_G0291992
DPU_G0053944
DLA_04379
DFA_02822
XP_004336679
0.1
DDB_G0275539
DDB_G0275539
DPU1256596
DLA_06660
PPL_09180
DFA_06013
0.1
RPT
DG1007
DDB_G0269232
DPU_G0056518
DLA_08039
PPL_09682
DFA_08509
0.1
DDB_G0279945
PPL_07933
DFA_01637
DDB_G0279945
DPU_G0074024
XP_004334562
XP_005826412
Phypo_04844
0.1
signal peptide
transmembrane
DG1039
DDB_G0284037
DPU_G0055450
DLA_0543
PPL_0729
DFA_0158
Phypo_03434
EIE84872
0.1
USP8_dimer
JAB_MPN
DDB_G0279727
DDB_G0279727
DPU_G0060524
Glyco_hydro_16

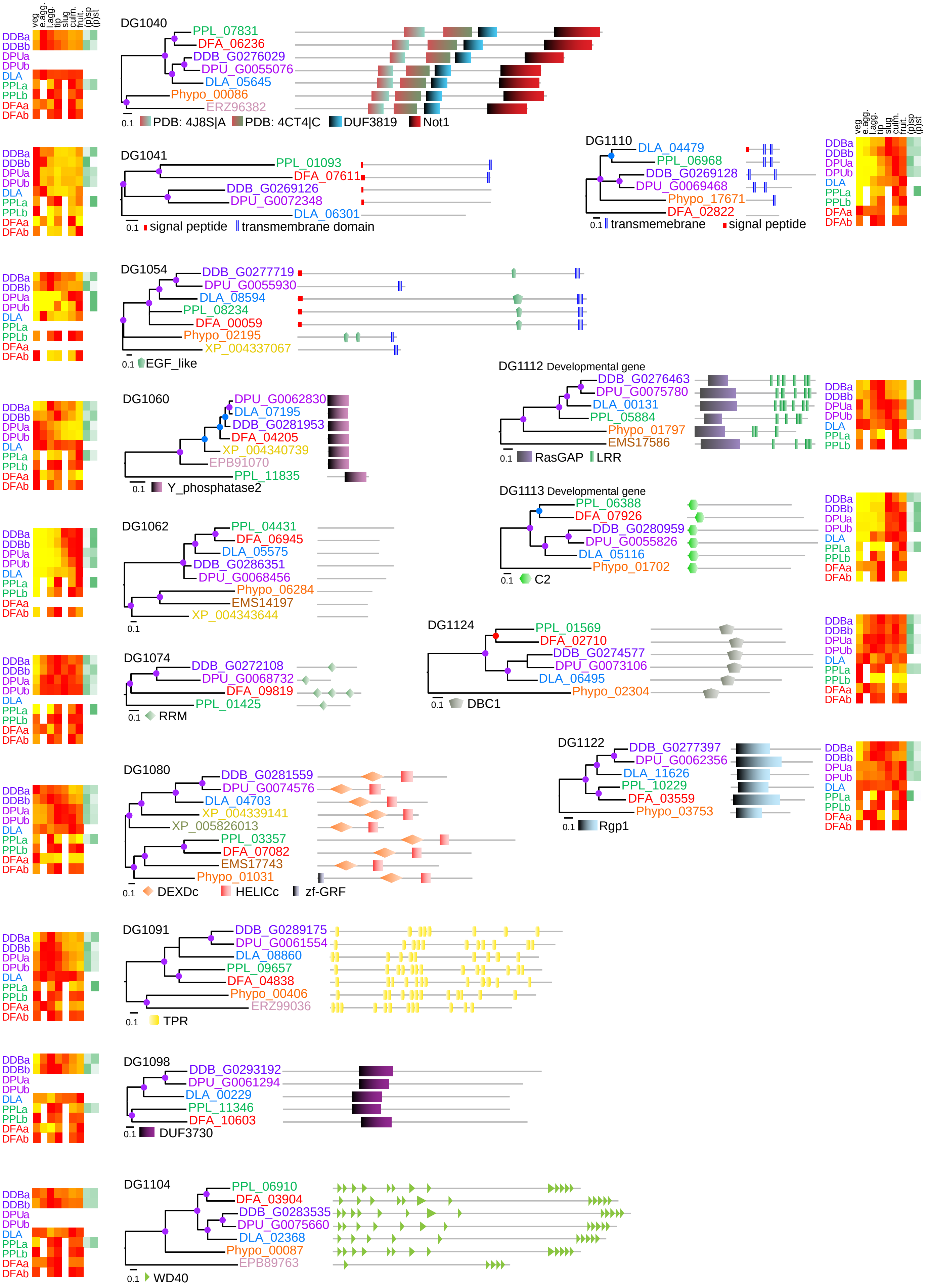
veg e.agg. l.agg. tip slug culm. fruit. (p)sp (p)st
DDBa DDBb DPUa DPUb DLA PPLa PPLb DFAa DFAb
DG1040
PPL_07831
DFA_06236
DDB_G0276029
DPU_G0055076
DLA_05645
Phypo_00086
ERZ96382
0.1 PDB: 4J8S|A PDB: 4CT4|C DUF3819 Not1
DG1041
PPL_01093
DFA_07611
DDB_G0269126
DPU_G0072348
DLA_06301
0.1 signal peptide transmembrane domain
DG1110
DLA_04479
PPL_06968
DDB_G0269128
DPU_G0069468
Phypo_17671
DFA_02822
0.1 transmemebrane signal peptide
DG1054
DDB_G0277719
DPU_G0055930
DLA_08594
PPL_08234
DFA_00059
Phypo_02195
XP_004337067
0.1 EGF_like
DG1112 Developmental gene
DDB_G0276463
DPU_G0075780
DLA_00131
PPL_05884
Phypo_01797
EMS17586
0.1 RasGAP LRR
DG1060
DPU_G0062830
DLA_07195
DDB_G0281953
DFA_04205
XP_004340739
EPB91070
PPL_11835
0.1 Y_phosphatase2
DG1113 Developmental gene
PPL_06388
DFA_07926
DDB_G0280959
DPU_G0055826
DLA_05116
Phypo_01702
0.1 C2
DG1062
PPL_04431
DFA_06945
DLA_05575
DDB_G0286351
DPU_G0068456
Phypo_06284
EMS14197
XP_004343644
0.1
DG1124
PPL_01569
DFA_02710
DDB_G0274577
DPU_G0073106
DLA_06495
Phypo_02304
0.1 DBC1
DG1074
DDB_G0272108
DPU_G0068732
DFA_09819
PPL_01425
0.1 RRM
DG1122
DDB_G0277397
DPU_G0062356
DLA_11626
PPL_10229
DFA_03559
Phypo_03753
0.1 Rgp1
DG1080
DDB_G0281559
DPU_G0074576
DLA_04703
XP_004339141
XP_005826013
PPL_03357
DFA_07082
EMS17743
Phypo_01031
0.1 DEXDc HELICc zf-GRF
DG1091
DDB_G0289175
DPU_G0061554
DLA_08860
PPL_09657
DFA_04838
Phypo_00406
ERZ99036
0.1 TPR
DG1098
DDB_G0293192
DPU_G0061294
DLA_00229
PPL_11346
DFA_10603
0.1 DUF3730
DG1104
PPL_06910
DFA_03904
DDB_G0283535
DPU_G0075660
DLA_02368
Phypo_00087
EPB89763
0.1 WD40

DG2033-Dn

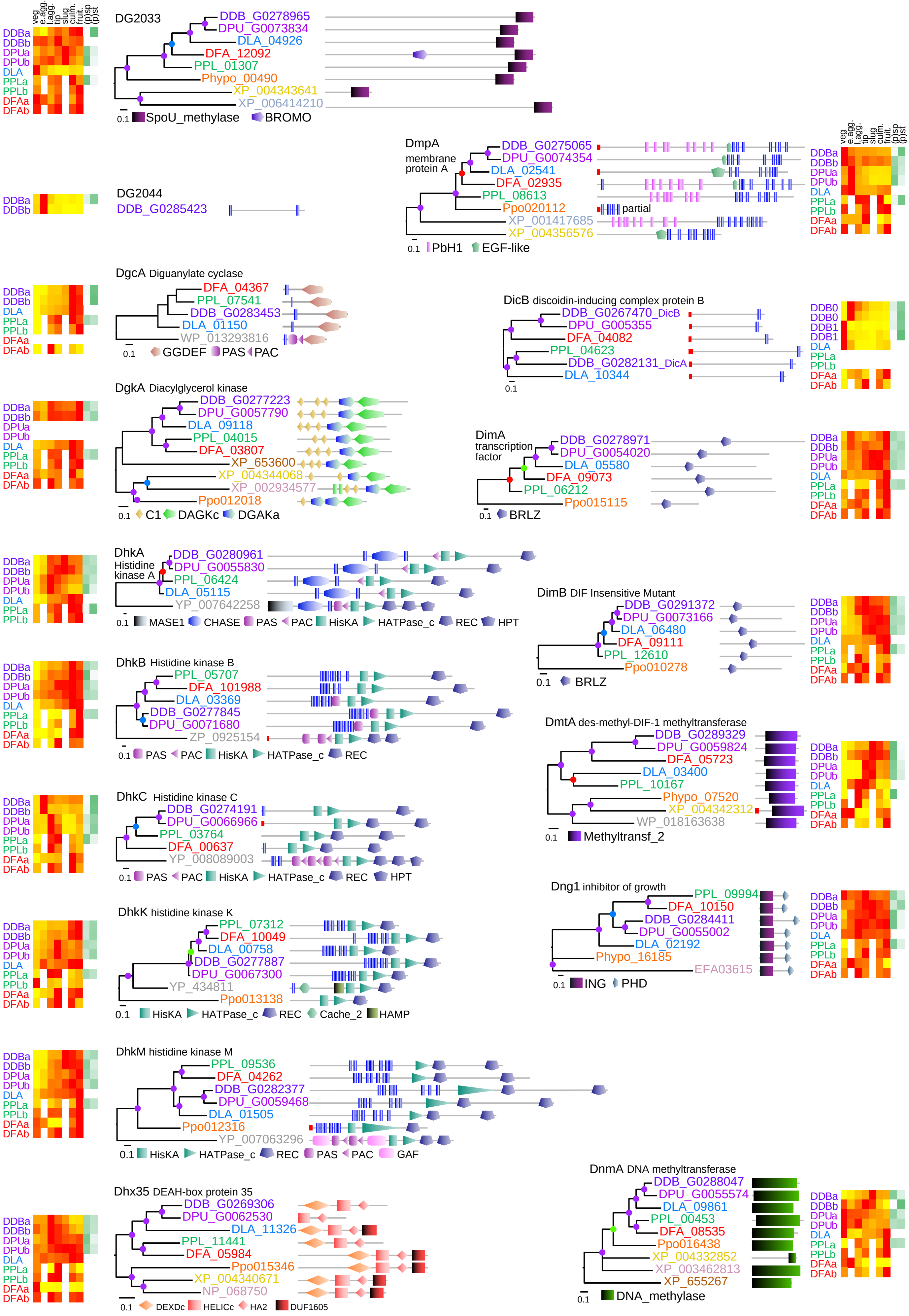

veg e.agg. l.agg. tip slug culm. fruit. (p)sp (p)st
DDBa DDBb DPUa DPUb DLA PPLa PPLb DFAa DFAb
DG2033
DDB_G0278965
DPU_G0073834
DLA_04926
DFA_12092
PPL_01307
Phypo_00490
XP_004343641
XP_006414210
0.1 SpoU_methylase BROMO
DG2044
DDB_G0285423
DmpA membrane protein A
DDB_G0275065
DPU_G0074354
DLA_02541
DFA_02935
PPL_08613
Ppo020112 partial
XP_001417685
XP_004356576
0.1 PbH1 EGF-like
DgcA Diguanylate cyclase
DFA_04367
PPL_07541
DDB_G0283453
DLA_01150
WP_013293816
0.1 GGDEF PAS PAC
DicB discoidin-inducing complex protein B
DDB_G0267470_DicB
DPU_G005355
DFA_04082
PPL_04623
DDB_G0282131_DicA
DLA_10344
DDB0 DDB0 DDB1 DDB1 DLA PPLa PPLb DFAa DFAb
DgkA Diacylglycerol kinase
DDB_G0277223
DPU_G0057790
DLA_09118
PPL_04015
DFA_03807
XP_653600
XP_004344068
XP_002934577
Ppo012018
0.1 C1 DAGKc DGAKa
DimA transcription factor
DDB_G0278971
DPU_G0054020
DLA_05580
DFA_09073
PPL_06212
Ppo015115
0.1 BRLZ
DhkA Histidine kinase A
DDB_G0280961
DPU_G0055830
PPL_06424
DLA_05115
YP_007642258
0.1 MASE1 CHASE PAS PAC HisKA HATPase_c REC HPT
DimB DIF Insensitive Mutant
DDB_G0291372
DPU_G0073166
DLA_06480
DFA_09111
PPL_12610
Ppo010278
0.1 BRLZ
DhkB Histidine kinase B
PPL_05707
DFA_101988
DLA_03369
DDB_G0277845
DPU_G0071680
ZP_0925154
0.1 PAS PAC HisKA HATPase_c REC
DmtA des-methyl-DIF-1 methyltransferase
DDB_G0289329
DPU_G0059824
DFA_05723
DLA_03400
PPL_10167
Phypo_07520
XP_004342312
WP_018163638
0.1 Methyltransf_2
DhkC Histidine kinase C
DDB_G0274191
DPU_G0066966
PPL_03764
DFA_00637
YP_008089003
0.1 PAS PAC HisKA HATPase_c REC HPT
Dng1 inhibitor of growth
PPL_09994
DFA_10150
DDB_G0284411
DPU_G0055002
DLA_02192
Phypo_16185
EFA03615
0.1 ING PHD
DhkK histidine kinase K
PPL_07312
DFA_10049
DLA_00758
DDB_G0277887
DPU_G0067300
YP_434811
Ppo013138
0.1 HisKA HATPase_c REC Cache_2 HAMP
DhkM histidine kinase M
PPL_09536
DFA_04262
DDB_G0282377
DPU_G0059468
DLA_01505
Ppo012316
YP_007063296
0.1 HisKA HATPase_c REC PAS PAC GAF
DnmA DNA methyltransferase
DDB_G0288047
DPU_G0055574
DLA_09861
PPL_00453
DFA_08535
Ppo016438
XP_004332852
XP_003462813
XP_655267
0.1 DNA_methylase
Dhx35 DEAH-box protein 35
DDB_G0269306
DPU_G0062530
DLA_11326
PPL_11441
DFA_05984
Ppo015346
XP_004340671
NP_068750
0.1 DEXDc HELICc HA2 DUF1605

Do-Fr

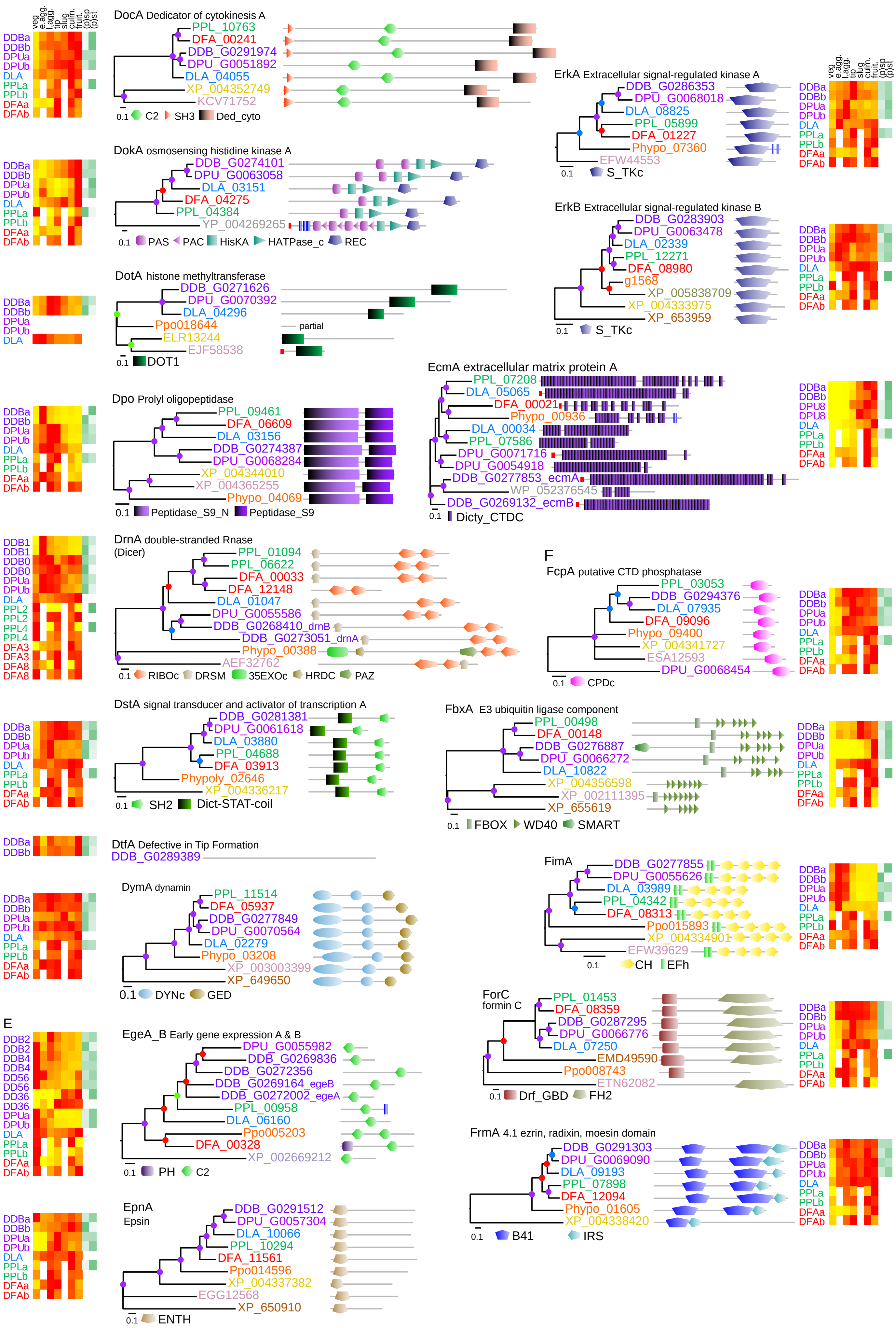

veg
e.agg.
l.agg.
tip
slug
culm.
fruit.
(p)sp
(p)st
DocA Dedicator of cytokinesis A
PPL_10763
DFA_00241
DDB_G0291974
DPU_G0051892
DLA_04055
XP_004352749
KCV71752
0.1
C2
SH3
Ded_cyto
DokA osmosensing histidine kinase A
DDB_G0274101
DPU_G0063058
DLA_03151
DFA_04275
PPL_04384
YP_004269265
PAS
PAC
HisKA
HATPase_c
REC
DotA histone methyltransferase
DDB_G0271626
DPU_G0070392
DLA_04296
Ppo018644
ELR13244
EJF58538
partial
DOT1
Dpo Prolyl oligopeptidase
PPL_09461
DFA_06609
DLA_03156
DDB_G0274387
DPU_G0068284
XP_004344010
XP_004365255
Phypo_04069
Peptidase_S9_N
Peptidase_S9
DrnA double-stranded Rnase
(Dicer)
PPL_01094
PPL_06622
DFA_00033
DFA_12148
DLA_01047
DPU_G0055586
DDB_G0268410_drnB
DDB_G0273051_drnA
Phypo_00388
AEF32762
RIBOc
DRSM
35EXOc
HRDC
PAZ
DstA signal transducer and activator of transcription A
DDB_G0281381
DPU_G0061618
DLA_03880
PPL_04688
DFA_03913
Phypoly_02646
XP_004336217
SH2
Dict-STAT-coil
DtfA Defective in Tip Formation
DDB_G0289389
DymA dynamin
PPL_11514
DFA_05937
DDB_G0277849
DPU_G0070564
DLA_02279
Phypo_03208
XP_003003399
XP_649650
DYNc
GED
E
EgeA_B Early gene expression A & B
DPU_G0055982
DDB_G0269836
DDB_G0272356
DDB_G0269164_egeB
DDB_G0272002_egeA
PPL_00958
DLA_06160
Ppo005203
DFA_00328
XP_002669212
PH
EpnA
Epsin
DDB_G0291512
DPU_G0057304
DLA_10066
PPL_10294
DFA_11561
Ppo014596
XP_004337382
EGG12568
XP_650910
ENTH
ErkA Extracellular signal-regulated kinase A
DDB_G0286353
DPU_G0068018
DLA_08825
PPL_05899
DFA_01227
Phypo_07360
EFW44553
S_TKc
ErkB Extracellular signal-regulated kinase B
DDB_G0283903
DPU_G0063478
DLA_02339
PPL_12271
DFA_08980
g1568
XP_005838709
XP_004333975
XP_653959
EcmA extracellular matrix protein A
PPL_07208
DLA_05065
DFA_00021
Phypo_00936
DLA_00034
PPL_07586
DPU_G0071716
DPU_G0054918
DDB_G0277853_ecmA
WP_052376545
DDB_G0269132_ecmB
Dicty_CTDC
F
FcpA putative CTD phosphatase
PPL_03053
DDB_G0294376
DLA_07935
DFA_09096
Phypo_09400
XP_004341727
ESA12593
DPU_G0068454
CPDc
FbxA E3 ubiquitin ligase component
PPL_00498
DFA_00148
DDB_G0276887
DPU_G0066272
DLA_10822
XP_004356598
XP_002111395
XP_655619
FBOX
WD40
SMART
FimA
DDB_G0277855
DPU_G0055626
DLA_03989
PPL_04342
DFA_08313
Ppo015893
XP_004334901
EFW39629
CH
EFh
ForC
formin C
PPL_01453
DFA_08359
DDB_G0287295
DPU_G0066776
DLA_07250
EMD49590
Ppo008743
ETN62082
Drf_GBD
FH2
FrmA 4.1 ezrin, radixin, moesin domain
DDB_G0291303
DPU_G0069090
DLA_09193
PPL_07898
DFA_12094
Phypo_01605
XP_004338420
B41
IRS

Fu-GefB

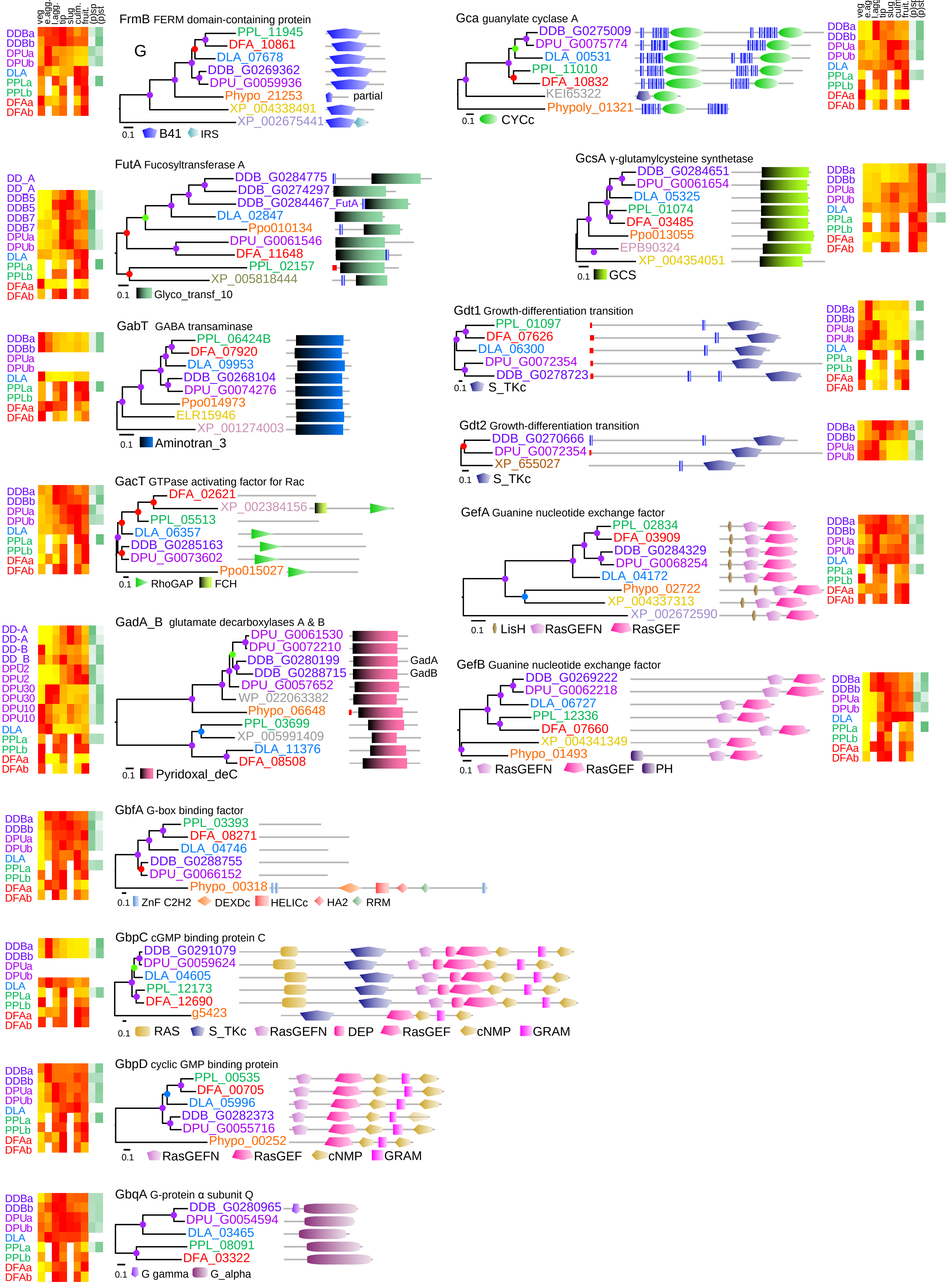
veg
e.agg.
l.agg.
tip
slug
culm.
fruit.
(p)sp
(p)st
FrmB FERM domain-containing protein
G
PPL_11945
DFA_10861
DLA_07678
DDB_G0269362
DPU_G0059936
Phypo_21253
XP_004338491
XP_002675441
partial
B41
IRS
Gca guanylate cyclase A
DDB_G0275009
DPU_G0075774
DLA_00531
PPL_11010
DFA_10832
KEI65322
Phypoly_01321
CYCc
FutA Fucosyltransferase A
DD_A
DDB5
DDB7
DDB_G0284775
DDB_G0274297
DDB_G0284467_FutA
DLA_02847
Ppo010134
DPU_G0061546
DFA_11648
PPL_02157
XP_005818444
Glyco_transf_10
GcsA γ-glutamylcysteine synthetase
DDB_G0284651
DPU_G0061654
DLA_05325
PPL_01074
DFA_03485
Ppo013055
EPB90324
XP_004354051
GCS
GabT GABA transaminase
PPL_06424B
DFA_07920
DLA_09953
DDB_G0268104
DPU_G0074276
Ppo014973
ELR15946
XP_001274003
Aminotran_3
Gdt1 Growth-differentiation transition
PPL_01097
DFA_07626
DLA_06300
DPU_G0072354
DDB_G0278723
S_TKc
Gdt2 Growth-differentiation transition
DDB_G0270666
DPU_G0072354
XP_655027
S_TKc
GacT GTPase activating factor for Rac
DFA_02621
XP_002384156
PPL_05513
DLA_06357
DDB_G0285163
DPU_G0073602
Ppo015027
RhoGAP
FCH
GefA Guanine nucleotide exchange factor
PPL_02834
DFA_03909
DDB_G0284329
DPU_G0068254
DLA_04172
Phypo_02722
XP_004337313
XP_002672590
LisH
RasGEFN
RasGEF
GadA_B glutamate decarboxylases A & B
DD-A
DD-B
DPU2
DPU30
DPU10
DPU_G0061530
DPU_G0072210
DDB_G0280199
DDB_G0288715
DPU_G0057652
WP_022063382
Phypo_06648
PPL_03699
XP_005991409
DLA_11376
DFA_08508
GadA
GadB
Pyridoxal_deC
GefB Guanine nucleotide exchange factor
DDB_G0269222
DPU_G0062218
DLA_06727
PPL_12336
DFA_07660
XP_004341349
Phypo_01493
RasGEFN
RasGEF
PH
GbfA G-box binding factor
PPL_03393
DFA_08271
DLA_04746
DDB_G0288755
DPU_G0066152
Phypo_00318
ZnF C2H2
DEXDc
HELICc
HA2
RRM
GbpC cGMP binding protein C
DDB_G0291079
DPU_G0059624
DLA_04605
PPL_12173
DFA_12690
g5423
RAS
S_TKc
RasGEFN
DEP
RasGEF
cNMP
GRAM
GbpD cyclic GMP binding protein
PPL_00535
DFA_00705
DLA_05996
DDB_G0282373
DPU_G0055716
Phypo_00252
RasGEFN
RasGEF
cNMP
GRAM
GbqA G-protein α subunit Q
DDB_G0280965
DPU_G0054594
DLA_03465
PPL_08091
DFA_03322
G gamma
G_alpha
DDBa
DDBb
DPUa
DPUb
DLA
PPLa
PPLb
DFAa
DFAb
0.1

GefL-Grl

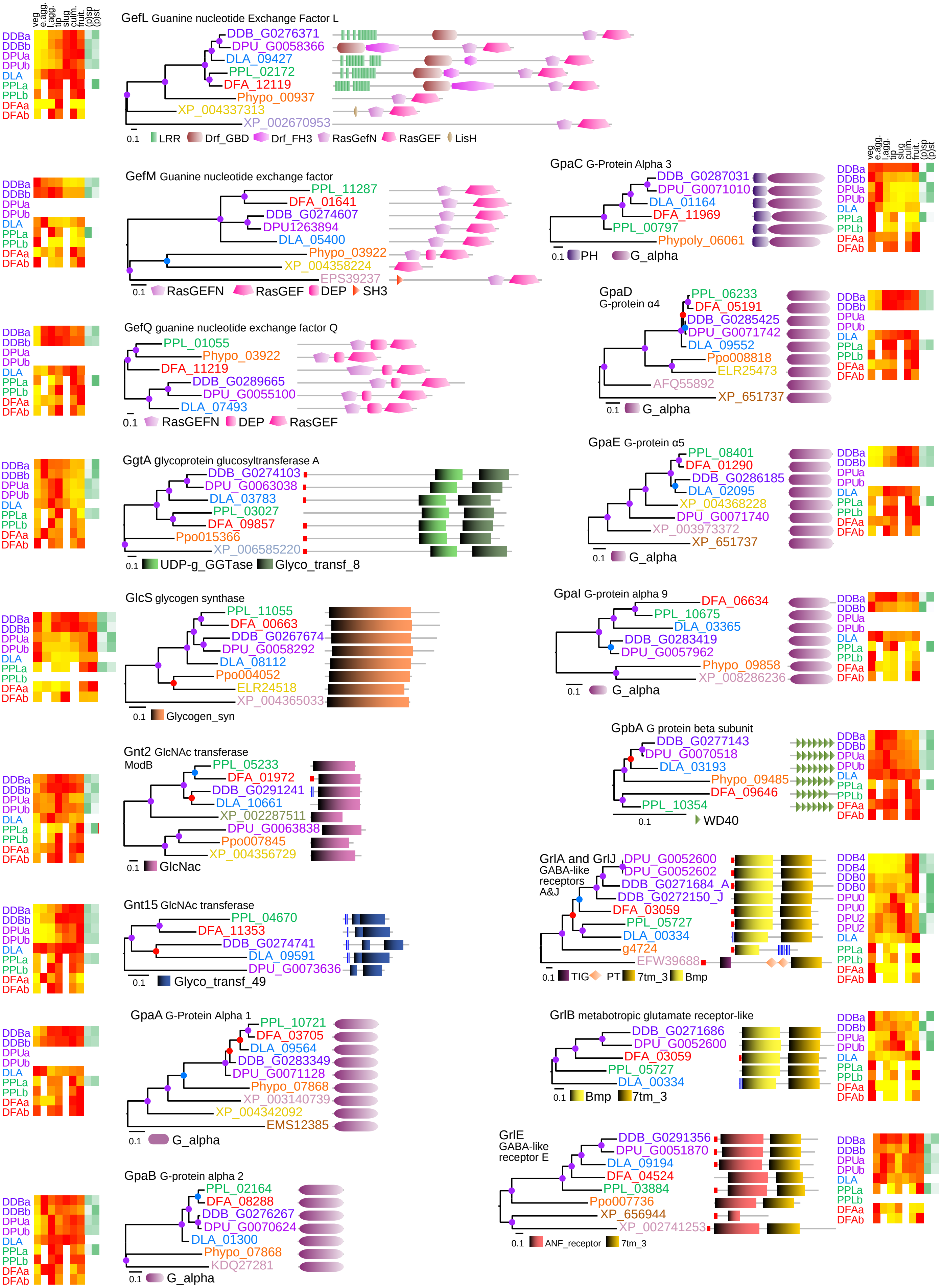
veg
e.agg.
l.agg.
tip
slug
culm.
fruit.
(p)sp
(p)st
DDBa
DDBb
DPUa
DPUb
DLA
PPLa
PPLb
DFAa
DFAb
GefL Guanine nucleotide Exchange Factor L
DDB_G0276371
DPU_G0058366
DLA_09427
PPL_02172
DFA_12119
Phypo_00937
XP_004337313
XP_002670953
0.1
LRR
Drf_GBD
Drf_FH3
RasGefN
RasGEF
LisH
GefM Guanine nucleotide exchange factor
PPL_11287
DFA_01641
DDB_G0274607
DPU1263894
DLA_05400
Phypo_03922
XP_004358224
EPS39237
RasGEFN
RasGEF
DEP
SH3
GefQ guanine nucleotide exchange factor Q
PPL_01055
Phypo_03922
DFA_11219
DDB_G0289665
DPU_G0055100
DLA_07493
GgtA glycoprotein glucosyltransferase A
DDB_G0274103
DPU_G0063038
DLA_03783
PPL_03027
DFA_09857
Ppo015366
XP_006585220
UDP-g_GGTase
Glyco_transf_8
GlcS glycogen synthase
PPL_11055
DFA_00663
DDB_G0267674
DPU_G0058292
DLA_08112
Ppo004052
ELR24518
XP_004365033
Glycogen_syn
Gnt2 GlcNAc transferase
ModB
PPL_05233
DFA_01972
DDB_G0291241
DLA_10661
XP_002287511
DPU_G0063838
Ppo007845
XP_004356729
GlcNac
Gnt15 GlcNAc transferase
PPL_04670
DFA_11353
DDB_G0274741
DLA_09591
DPU_G0073636
Glyco_transf_49
GpaA G-Protein Alpha 1
PPL_10721
DFA_03705
DLA_09564
DDB_G0283349
DPU_G0071128
Phypo_07868
XP_003140739
XP_004342092
EMS12385
G_alpha
GpaB G-protein alpha 2
PPL_02164
DFA_08288
DDB_G0276267
DPU_G0070624
DLA_01300
Phypo_07868
KDQ27281
GpaC G-Protein Alpha 3
DDB_G0287031
DPU_G0071010
DLA_01164
DFA_11969
PPL_00797
Phypoly_06061
PH
GpaD
G-protein α4
PPL_06233
DFA_05191
DDB_G0285425
DPU_G0071742
DLA_09552
Ppo008818
ELR25473
AFQ55892
XP_651737
GpaE G-protein α5
PPL_08401
DFA_01290
DDB_G0286185
DLA_02095
XP_004368228
DPU_G0071740
XP_003973372
XP_651737
GpaI G-protein alpha 9
DFA_06634
PPL_10675
DLA_03365
DDB_G0283419
DPU_G0057962
Phypo_09858
XP_008286236
GpbA G protein beta subunit
DDB_G0277143
DPU_G0070518
DLA_03193
Phypo_09485
DFA_09646
PPL_10354
WD40
GrlA and GrlJ
GABA-like
receptors
A&J
DPU_G0052600
DPU_G0052602
DDB_G0271684_A
DDB_G0272150_J
DFA_03059
PPL_05727
DLA_00334
g4724
EFW39688
TIG
PT
7tm_3
Bmp
DDB4
DDB0
DPU0
DPU2
GrlB metabotropic glutamate receptor-like
DDB_G0271686
DPU_G0052600
DFA_03059
PPL_05727
DLA_00334
GrlE
GABA-like
receptor E
DDB_G0291356
DPU_G0051870
DLA_09194
DFA_04524
PPL_03884
Ppo007736
XP_656944
XP_002741253
ANF_receptor

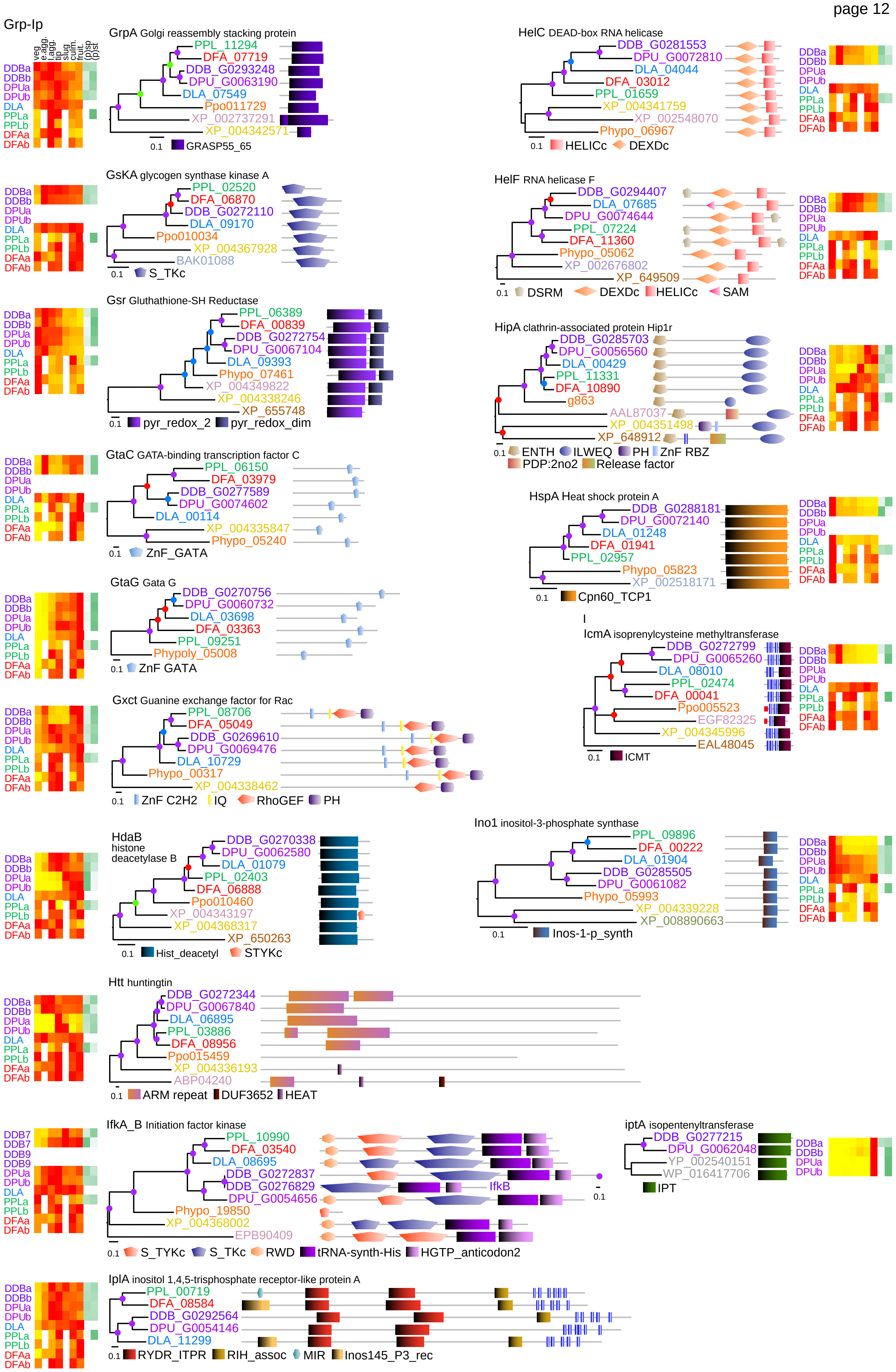

Grp-Ip
veg
e.agg.
l.agg.
tip
slug
culm.
fruit.
(p)sp
(p)st
DDBa
DDBb
DPUa
DPUb
DLA
PPLa
PPLb
DFAa
DFAb
GrpA Golgi reassembly stacking protein
PPL_11294
DFA_07719
DDB_G0293248
DPU_G0063190
DLA_07549
Ppo011729
XP_002737291
XP_004342571
0.1
GRASP55_65
GsKA glycogen synthase kinase A
PPL_02520
DFA_06870
DDB_G0272110
DLA_09170
Ppo010034
XP_004367928
BAK01088
0.1
S_TKc
Gsr Gluthathione-SH Reductase
PPL_06389
DFA_00839
DDB_G0272754
DPU_G0067104
DLA_09393
Phypo_07461
XP_004349822
XP_004338246
XP_655748
0.1
pyr_redox_2
pyr_redox_dim
GtaC GATA-binding transcription factor C
PPL_06150
DFA_03979
DDB_G0277589
DPU_G0074602
DLA_00114
XP_004335847
Phypo_05240
0.1
ZnF_GATA
GtaG Gata G
DDB_G0270756
DPU_G0060732
DLA_03698
DFA_03363
PPL_09251
Phypoly_05008
0.1
ZnF GATA
Gxct Guanine exchange factor for Rac
PPL_08706
DFA_05049
DDB_G0269610
DPU_G0069476
DLA_10729
Phypo_00317
XP_004338462
0.1
ZnF C2H2
IQ
RhoGEF
PH
HdaB
histone
deacetylase B
DDB_G0270338
DPU_G0062580
DLA_01079
PPL_02403
DFA_06888
Ppo010460
XP_004343197
XP_004368317
XP_650263
0.1
Hist_deacetyl
STYKc
Htt huntingtin
DDB_G0272344
DPU_G0067840
DLA_06895
PPL_03886
DFA_08956
Ppo015459
XP_004336193
ABP04240
0.1
ARM repeat
DUF3652
HEAT
IfkA_B Initiation factor kinase
DDB7
DDB7
DDB9
DDB9
PPL_10990
DFA_03540
DLA_08695
DDB_G0272837
DDB_G0276829
DPU_G0054656
Phypo_19850
XP_004368002
EPB90409
IfkB
0.1
S_TYKc
S_TKc
RWD
tRNA-synth-His
HGTP_anticodon2
IplA inositol 1,4,5-trisphosphate receptor-like protein A
PPL_00719
DFA_08584
DDB_G0292564
DPU_G0054146
DLA_11299
0.1
RYDR_ITPR
RIH_assoc
MIR
Inos145_P3_rec
HelC DEAD-box RNA helicase
DDB_G0281553
DPU_G0072810
DLA_04044
DFA_03012
PPL_01659
XP_004341759
XP_002548070
Phypo_06967
0.1
HELICc
DEXDc
HelF RNA helicase F
DDB_G0294407
DLA_07685
DPU_G0074644
PPL_07224
DFA_11360
Phypo_05062
XP_002676802
XP_649509
0.1
DSRM
DEXDc
HELICc
SAM
HipA clathrin-associated protein Hip1r
DDB_G0285703
DPU_G0056560
DLA_00429
PPL_11331
DFA_10890
g863
AAL87037
XP_004351498
XP_648912
0.1
ENTH
ILWEQ
PH
ZnF RBZ
PDP:2no2
Release factor
HspA Heat shock protein A
DDB_G0288181
DPU_G0072140
DLA_01248
DFA_01941
PPL_02957
Phypo_05823
XP_002518171
0.1
Cpn60_TCP1
IcmA isoprenylcysteine methyltransferase
DDB_G0272799
DPU_G0065260
DLA_08010
PPL_02474
DFA_00041
Ppo005523
EGF82325
XP_004345996
EAL48045
0.1
ICMT
Ino1 inositol-3-phosphate synthase
PPL_09896
DFA_00222
DLA_01904
DDB_G0285505
DPU_G0061082
Phypo_05993
XP_004339228
XP_008890663
0.1
Inos-1-p_synth
iptA isopentenyltransferase
DDB_G0277215
DPU_G0062048
YP_002540151
WP_016417706
IPT

Kc-MhkA

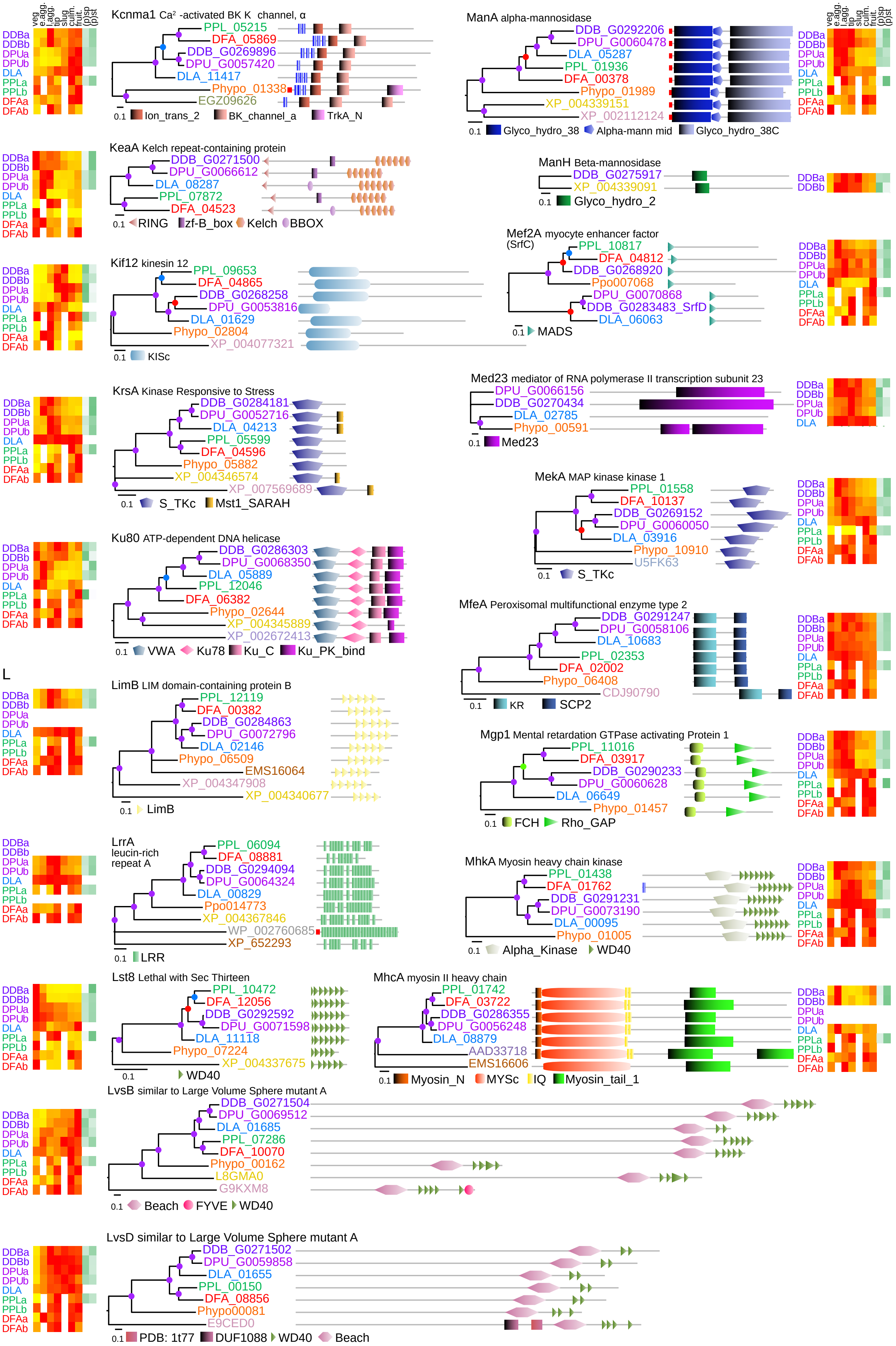
veg. e.agg. l.agg. tip slug culm. fruit. (p)sp (p)st
DDBa DDBb DPUa DPUb DLA PPLa PPLb DFAa DFAb
Kcnma1 Ca2 -activated BK K channel, α
PPL_05215
DFA_05869
DDB_G0269896
DPU_G0057420
DLA_11417
Phypo_01338
EGZ09626
0.1 Ion_trans_2 BK_channel_a TrkA_N
KeaA Kelch repeat-containing protein
DDB_G0271500
DPU_G0066612
DLA_08287
PPL_07872
DFA_04523
0.1 RING zf-B_box Kelch BBOX
Kif12 kinesin 12
PPL_09653
DFA_04865
DDB_G0268258
DPU_G0053816
DLA_01629
Phypo_02804
XP_004077321
0.1 KISc
KrsA Kinase Responsive to Stress
DDB_G0284181
DPU_G0052716
DLA_04213
PPL_05599
DFA_04596
Phypo_05882
XP_004346574
XP_007569689
0.1 S_TKc Mst1_SARAH
Ku80 ATP-dependent DNA helicase
DDB_G0286303
DPU_G0068350
DLA_05889
PPL_12046
DFA_06382
Phypo_02644
XP_004345889
XP_002672413
0.1 VWA Ku78 Ku_C Ku_PK_bind
L
LimB LIM domain-containing protein B
PPL_12119
DFA_00382
DDB_G0284863
DPU_G0072796
DLA_02146
Phypo_06509
EMS16064
XP_004347908
XP_004340677
0.1 LimB
LrrA leucin-rich repeat A
PPL_06094
DFA_08881
DDB_G0294094
DPU_G0064324
DLA_00829
Ppo014773
XP_004367846
WP_002760685
XP_652293
0.1 LRR
Lst8 Lethal with Sec Thirteen
PPL_10472
DFA_12056
DDB_G0292592
DPU_G0071598
DLA_11118
Phypo_07224
XP_004337675
0.1 WD40
LvsB similar to Large Volume Sphere mutant A
DDB_G0271504
DPU_G0069512
DLA_01685
PPL_07286
DFA_10070
Phypo_00162
L8GMA0
G9KXM8
0.1 Beach FYVE WD40
LvsD similar to Large Volume Sphere mutant A
DDB_G0271502
DPU_G0059858
DLA_01655
PPL_00150
DFA_08856
Phypo00081
E9CED0
0.1 PDB: 1t77 DUF1088 WD40 Beach
M
ManA alpha-mannosidase
DDB_G0292206
DPU_G0060478
DLA_05287
PPL_01936
DFA_00378
Phypo_01989
XP_004339151
XP_002112124
0.1 Glyco_hydro_38 Alpha-mann mid Glyco_hydro_38C
ManH Beta-mannosidase
DDB_G0275917
XP_004339091
0.1 Glyco_hydro_2
Mef2A myocyte enhancer factor (SrfC)
PPL_10817
DFA_04812
DDB_G0268920
Ppo007068
DPU_G0070868
DDB_G0283483_SrfD
DLA_06063
0.1 MADS
Med23 mediator of RNA polymerase II transcription subunit 23
DPU_G0066156
DDB_G0270434
DLA_02785
Phypo_00591
0.1 Med23
MekA MAP kinase kinase 1
PPL_01558
DFA_10137
DDB_G0269152
DPU_G0060050
DLA_03916
Phypo_10910
U5FK63
0.1 S_TKc
MfeA Peroxisomal multifunctional enzyme type 2
DDB_G0291247
DPU_G0058106
DLA_10683
PPL_02353
DFA_02002
Phypo_06408
CDJ90790
0.1 KR SCP2
Mgp1 Mental retardation GTPase activating Protein 1
PPL_11016
DFA_03917
DDB_G0290233
DPU_G0060628
DLA_06649
Phypo_01457
0.1 FCH Rho_GAP
MhkA Myosin heavy chain kinase
PPL_01438
DFA_01762
DDB_G0291231
DPU_G0073190
DLA_00095
Phypo_01005
0.1 Alpha_Kinase WD40
MhcA myosin II heavy chain
PPL_01742
DFA_03722
DDB_G0286355
DPU_G0056248
DLA_08879
AAD33718
EMS16606
0.1 Myosin_N MYSc IQ Myosin_tail_1

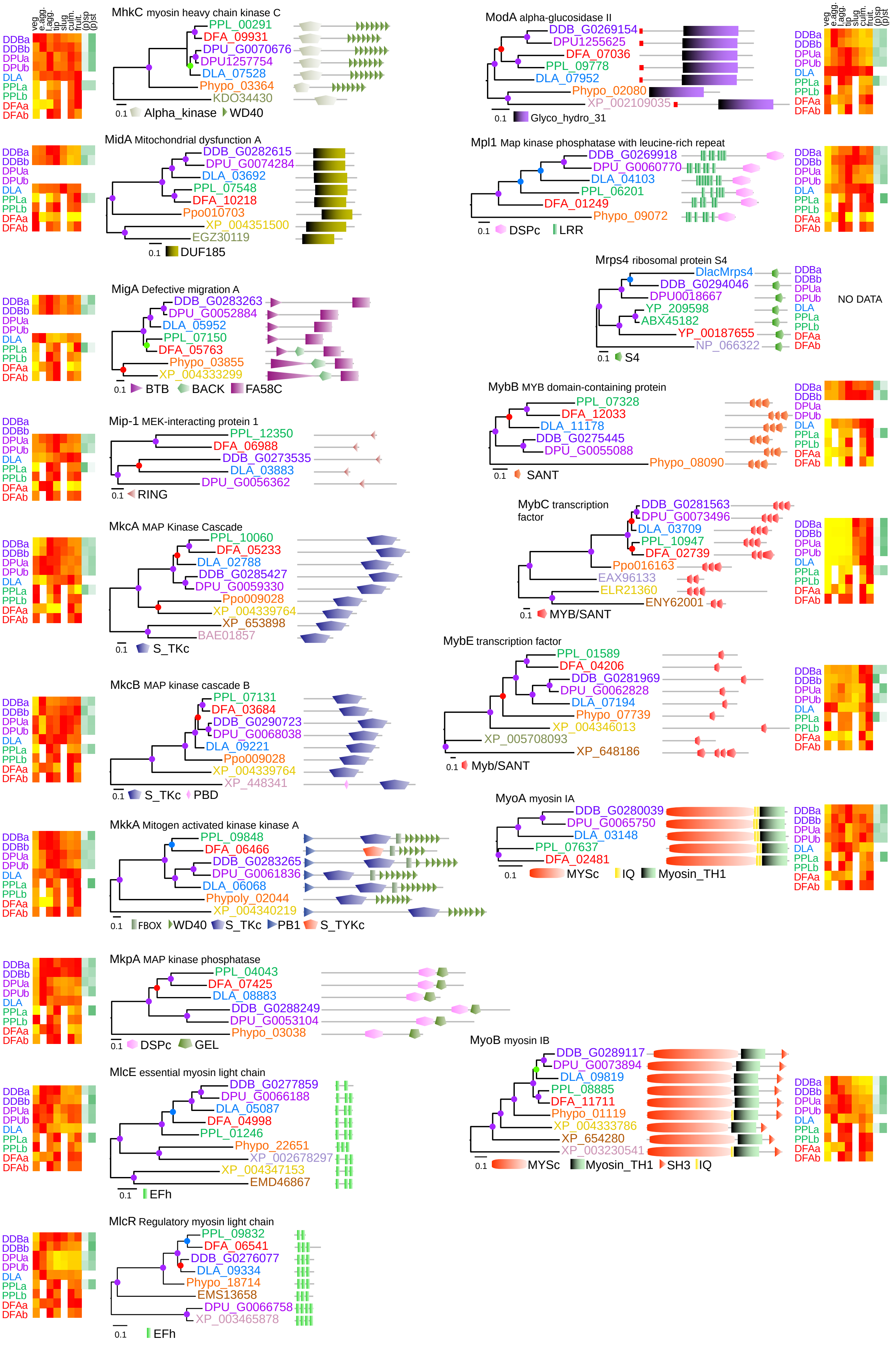
veg
e.agg.
l.agg.
tip
slug
culm.
fruit.
(p)sp
(p)st
MhkC myosin heavy chain kinase C
PPL_00291
DFA_09931
DPU_G0070676
DPU1257754
DLA_07528
Phypo_03364
KDO34430
0.1
Alpha_kinase
WD40
MidA Mitochondrial dysfunction A
DDB_G0282615
DPU_G0074284
DLA_03692
PPL_07548
DFA_10218
Ppo010703
XP_004351500
EGZ30119
DUF185
MigA Defective migration A
DDB_G0283263
DPU_G0052884
DLA_05952
PPL_07150
DFA_05763
Phypo_03855
XP_004333299
BTB
BACK
FA58C
Mip-1 MEK-interacting protein 1
PPL_12350
DFA_06988
DDB_G0273535
DLA_03883
DPU_G0056362
RING
MkcA MAP Kinase Cascade
PPL_10060
DFA_05233
DLA_02788
DDB_G0285427
DPU_G0059330
Ppo009028
XP_004339764
XP_653898
BAE01857
S_TKc
MkcB MAP kinase cascade B
PPL_07131
DFA_03684
DDB_G0290723
DPU_G0068038
DLA_09221
Ppo009028
XP_004339764
XP_448341
PBD
MkkA Mitogen activated kinase kinase A
PPL_09848
DFA_06466
DDB_G0283265
DPU_G0061836
DLA_06068
Phypoly_02044
XP_004340219
FBOX
PB1
S_TYKc
MkpA MAP kinase phosphatase
PPL_04043
DFA_07425
DLA_08883
DDB_G0288249
DPU_G0053104
Phypo_03038
DSPc
GEL
MlcE essential myosin light chain
DDB_G0277859
DPU_G0066188
DLA_05087
DFA_04998
PPL_01246
Phypo_22651
XP_002678297
XP_004347153
EMD46867
EFh
MlcR Regulatory myosin light chain
PPL_09832
DFA_06541
DDB_G0276077
DLA_09334
Phypo_18714
EMS13658
DPU_G0066758
XP_003465878
ModA alpha-glucosidase II
DDB_G0269154
DPU1255625
DFA_07036
PPL_09778
DLA_07952
Phypo_02080
XP_002109035
Glyco_hydro_31
Mpl1 Map kinase phosphatase with leucine-rich repeat
DDB_G0269918
DPU_G0060770
DLA_04103
PPL_06201
DFA_01249
Phypo_09072
LRR
Mrps4 ribosomal protein S4
DlacMrps4
DDB_G0294046
DPU0018667
YP_209598
ABX45182
YP_00187655
NP_066322
S4
NO DATA
MybB MYB domain-containing protein
PPL_07328
DFA_12033
DLA_11178
DDB_G0275445
DPU_G0055088
Phypo_08090
SANT
MybC transcription factor
DDB_G0281563
DPU_G0073496
DLA_03709
PPL_10947
DFA_02739
Ppo016163
EAX96133
ELR21360
ENY62001
MYB/SANT
MybE transcription factor
PPL_01589
DFA_04206
DDB_G0281969
DPU_G0062828
DLA_07194
Phypo_07739
XP_004346013
XP_005708093
XP_648186
Myb/SANT
MyoA myosin IA
DDB_G0280039
DPU_G0065750
DLA_03148
PPL_07637
DFA_02481
MYSc
IQ
Myosin_TH1
MyoB myosin IB
DDB_G0289117
DPU_G0073894
DLA_09819
PPL_08885
DFA_11711
Phypo_01119
XP_004333786
XP_654280
XP_003230541
SH3
DDBa
DDBb
DPUa
DPUb
DLA
PPLa
PPLb
DFAa
DFAb

MyoG-Pad

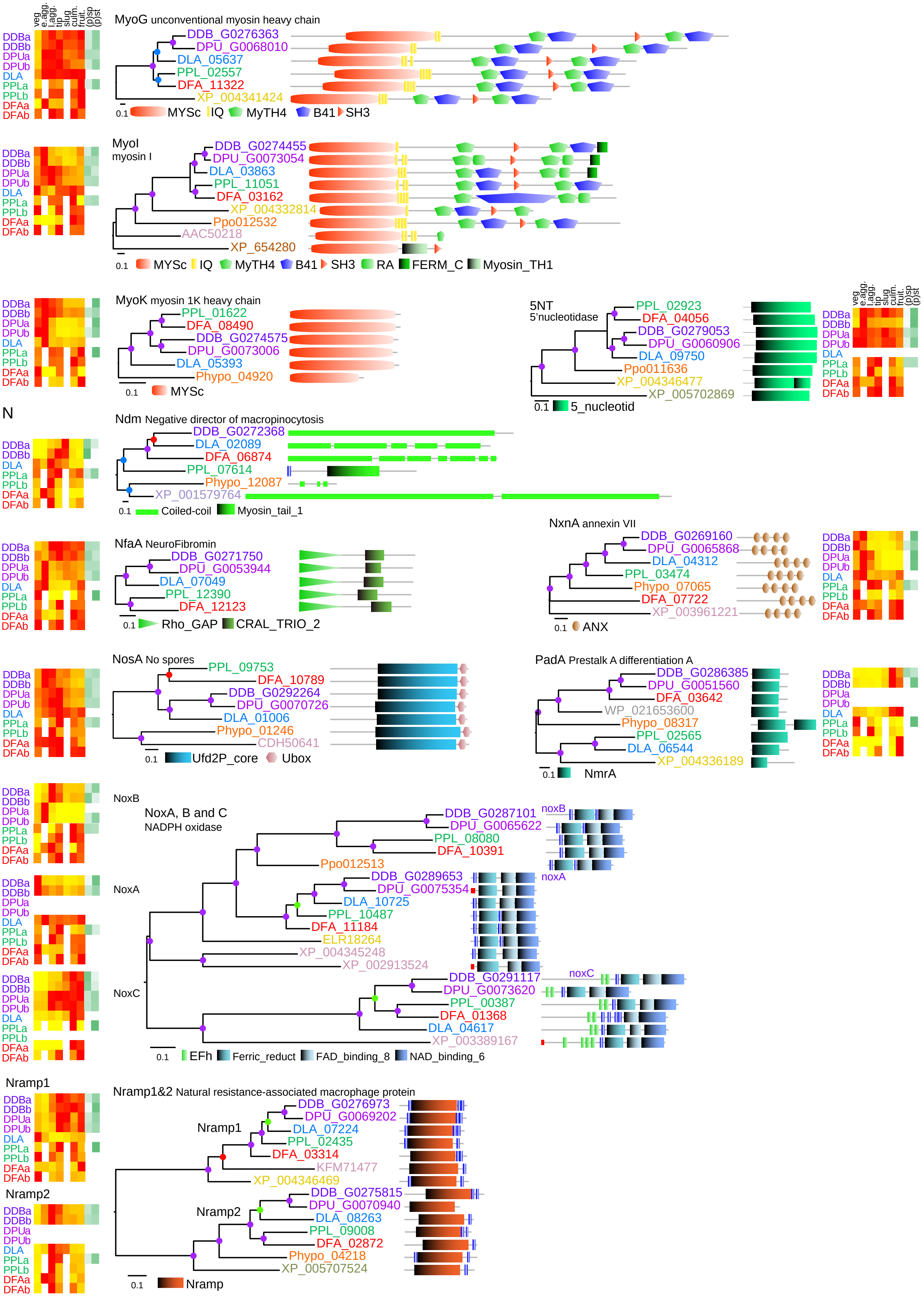

veg
e.agg.
l.agg.
tip
slug
culm.
fruit.
(p)sp
(p)st
DDBa
DDBb
DPUa
DPUb
DLA
PPLa
PPLb
DFAa
DFAb
MyoG unconventional myosin heavy chain
DDB_G0276363
DPU_G0068010
DLA_05637
PPL_02557
DFA_11322
XP_004341424
0.1
MYSc
IQ
MyTH4
B41
SH3
MyoI
myosin I
DDB_G0274455
DPU_G0073054
DLA_03863
PPL_11051
DFA_03162
XP_004332814
Ppo012532
AAC50218
XP_654280
0.1
MYSc
IQ
MyTH4
B41
SH3
RA
FERM_C
Myosin_TH1
MyoK myosin 1K heavy chain
PPL_01622
DFA_08490
DDB_G0274575
DPU_G0073006
DLA_05393
Phypo_04920
0.1
MYSc
5NT
5'nucleotidase
PPL_02923
DFA_04056
DDB_G0279053
DPU_G0060906
DLA_09750
Ppo011636
XP_004346477
XP_005702869
0.1
5_nucleotid
N
Ndm Negative director of macropinocytosis
DDB_G0272368
DLA_02089
DFA_06874
PPL_07614
Phypo_12087
XP_001579764
0.1
Coiled-coil
Myosin_tail_1
NxnA annexin VII
DDB_G0269160
DPU_G0065868
DLA_04312
PPL_03474
Phypo_07065
DFA_07722
XP_003961221
0.1
ANX
NfaA NeuroFibromin
DDB_G0271750
DPU_G0053944
DLA_07049
PPL_12390
DFA_12123
0.1
Rho_GAP
CRAL_TRIO_2
NosA No spores
PPL_09753
DFA_10789
DDB_G0292264
DPU_G0070726
DLA_01006
Phypo_01246
CDH50641
0.1
Ufd2P_core
Ubox
PadA Prestalk A differentiation A
DDB_G0286385
DPU_G0051560
DFA_03642
WP_021653600
Phypo_08317
PPL_02565
DLA_06544
XP_004336189
0.1
NmrA
NoxB
NoxA
NoxC
NoxA, B and C
NADPH oxidase
DDB_G0287101
DPU_G0065622
PPL_08080
DFA_10391
Ppo012513
DDB_G0289653
DPU_G0075354
DLA_10725
PPL_10487
DFA_11184
ELR18264
XP_004345248
XP_002913524
DDB_G0291117
DPU_G0073620
PPL_00387
DFA_01368
DLA_04617
XP_003389167
noxB
noxA
noxC
0.1
EFh
Ferric_reduct
FAD_binding_8
NAD_binding_6
Nramp1
Nramp2
Nramp1&2 Natural resistance-associated macrophage protein
DDB_G0276973
DPU_G0069202
DLA_07224
PPL_02435
DFA_03314
KFM71477
XP_004346469
DDB_G0275815
DPU_G0070940
DLA_08263
PPL_09008
DFA_02872
Phypo_04218
XP_005707524
0.1
Nramp

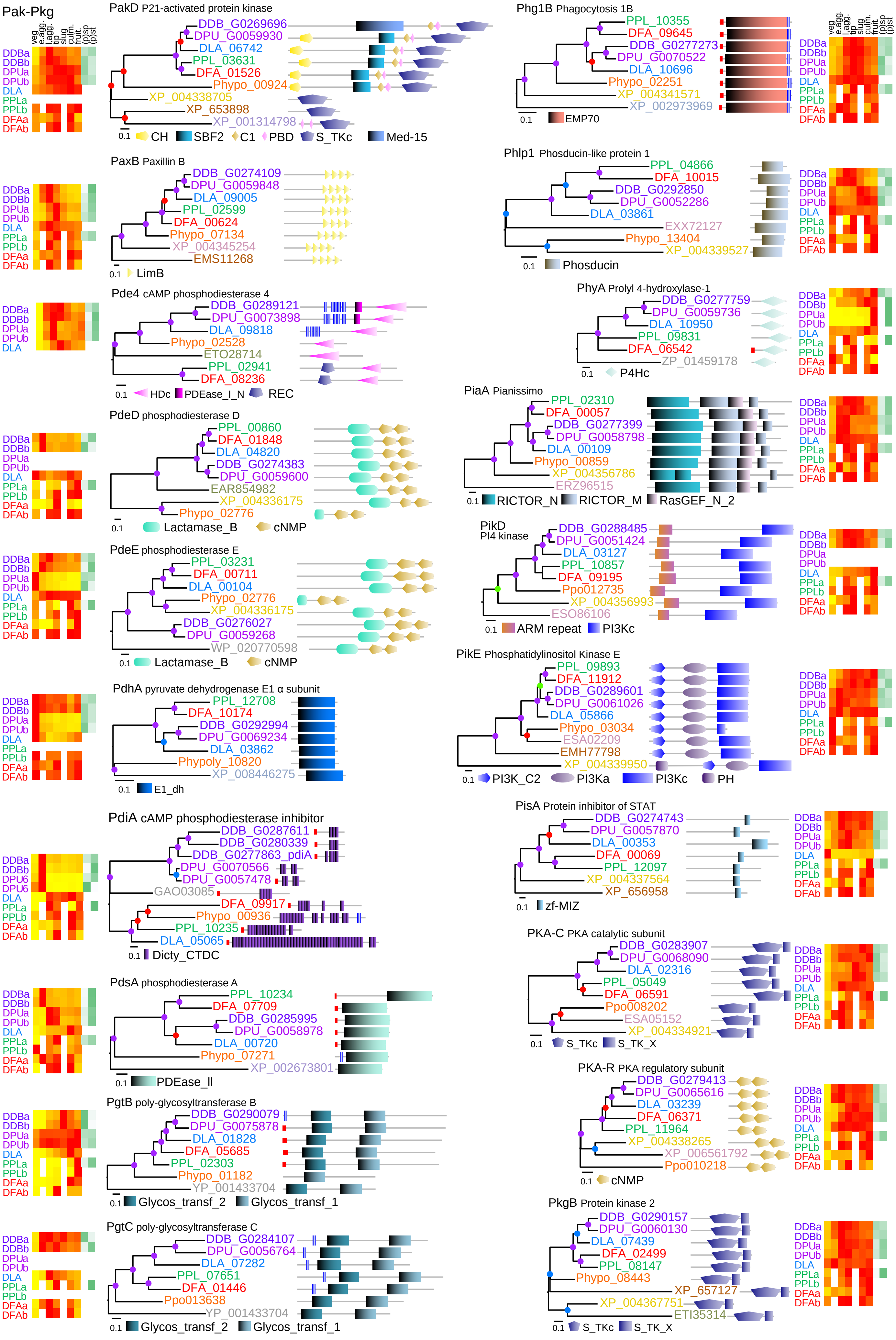
Pak-Pkg
veg
e.agg.
l.agg.
tip
slug
culm.
fruit.
(p)sp
(p)st
DDBa DDBb DPUa DPUb DLA PPLa PPLb DFAa DFAb
PakD P21-activated protein kinase
DDB_G0269696
DPU_G0059930
DLA_06742
PPL_03631
DFA_01526
Phypo_00924
XP_004338705
XP_653898
XP_001314798
0.1 CH SBF2 C1 PBD S_TKc Med-15
PaxB Paxillin B
DDB_G0274109
DPU_G0059848
DLA_09005
PPL_02599
DFA_00624
Phypo_07134
XP_004345254
EMS11268
0.1 LimB
Pde4 cAMP phosphodiesterase 4
DDB_G0289121
DPU_G0073898
DLA_09818
Phypo_02528
ETO28714
PPL_02941
DFA_08236
0.1 HDc PDEase_I_N REC
PdeD phosphodiesterase D
PPL_00860
DFA_01848
DLA_04820
DDB_G0274383
DPU_G0059600
EAR854982
XP_004336175
Phypo_02776
0.1 Lactamase_B cNMP
PdeE phosphodiesterase E
PPL_03231
DFA_00711
DLA_00104
Phypo_02776
XP_004336175
DDB_G0276027
DPU_G0059268
WP_020770598
0.1 Lactamase_B cNMP
PdhA pyruvate dehydrogenase E1 α subunit
PPL_12708
DFA_10174
DDB_G0292994
DPU_G0069234
DLA_03862
Phypoly_10820
XP_008446275
0.1 E1_dh
PdiA cAMP phosphodiesterase inhibitor
DDB_G0287611
DDB_G0280339
DDB_G0277863_pdiA
DPU_G0070566
DPU_G0057478
GAO03085
DFA_09917
Phypo_00936
PPL_10235
DLA_05065
0.1 Dicty_CTDC
DDBa DDBb DPU6 DPU6 DLA PPLa PPLb DFAa DFAb
PdsA phosphodiesterase A
PPL_10234
DFA_07709
DDB_G0285995
DPU_G0058978
DLA_00720
Phypo_07271
XP_002673801
0.1 PDEase_II
PgtB poly-glycosyltransferase B
DDB_G0290079
DPU_G0075878
DLA_01828
DFA_05685
PPL_02303
Phypo_01182
YP_001433704
0.1 Glycos_transf_2 Glycos_transf_1
PgtC poly-glycosyltransferase C
DDB_G0284107
DPU_G0056764
DLA_07282
PPL_07651
DFA_01446
Ppo013638
YP_001433704
0.1 Glycos_transf_2 Glycos_transf_1
Phg1B Phagocytosis 1B
PPL_10355
DFA_09645
DDB_G0277273
DPU_G0070522
DLA_10696
Phypo_02251
XP_004341571
XP_002973969
0.1 EMP70
Phlp1 Phosducin-like protein 1
PPL_04866
DFA_10015
DDB_G0292850
DPU_G0052286
DLA_03861
EXX72127
Phypo_13404
XP_004339527
0.1 Phosducin
PhyA Prolyl 4-hydroxylase-1
DDB_G0277759
DPU_G0059736
DLA_10950
PPL_09831
DFA_06542
ZP_01459178
0.1 P4Hc
PiaA Pianissimo
PPL_02310
DFA_00057
DDB_G0277399
DPU_G0058798
DLA_00109
Phypo_00859
XP_004356786
ERZ96515
0.1 RICTOR_N RICTOR_M RasGEF_N_2
PikD
PI4 kinase
DDB_G0288485
DPU_G0051424
DLA_03127
PPL_10857
DFA_09195
Ppo012735
XP_004356993
ESO86106
0.1 ARM repeat PI3Kc
PikE Phosphatidylinositol Kinase E
PPL_09893
DFA_11912
DDB_G0289601
DPU_G0061026
DLA_05866
Phypo_03034
ESA02209
EMH77798
XP_004339950
0.1 PI3K_C2 PI3Ka PI3Kc PH
PisA Protein inhibitor of STAT
DDB_G0274743
DPU_G0057870
DLA_00353
DFA_00069
PPL_12097
XP_004337564
XP_656958
0.1 zf-MIZ
PKA-C PKA catalytic subunit
DDB_G0283907
DPU_G0068090
DLA_02316
PPL_05049
DFA_06591
Ppo008202
ESA05152
XP_004334921
0.1 S_TKc S_TK_X
PKA-R PKA regulatory subunit
DDB_G0279413
DPU_G0065616
DLA_03239
DFA_06371
PPL_11964
XP_004338265
XP_006561792
Ppo010218
0.1 cNMP
PkgB Protein kinase 2
DDB_G0290157
DPU_G0060130
DLA_07439
DFA_02499
PPL_08147
Phypo_08443
XP_657127
XP_004367751
ETI35314
0.1 S_TKc S_TK_X

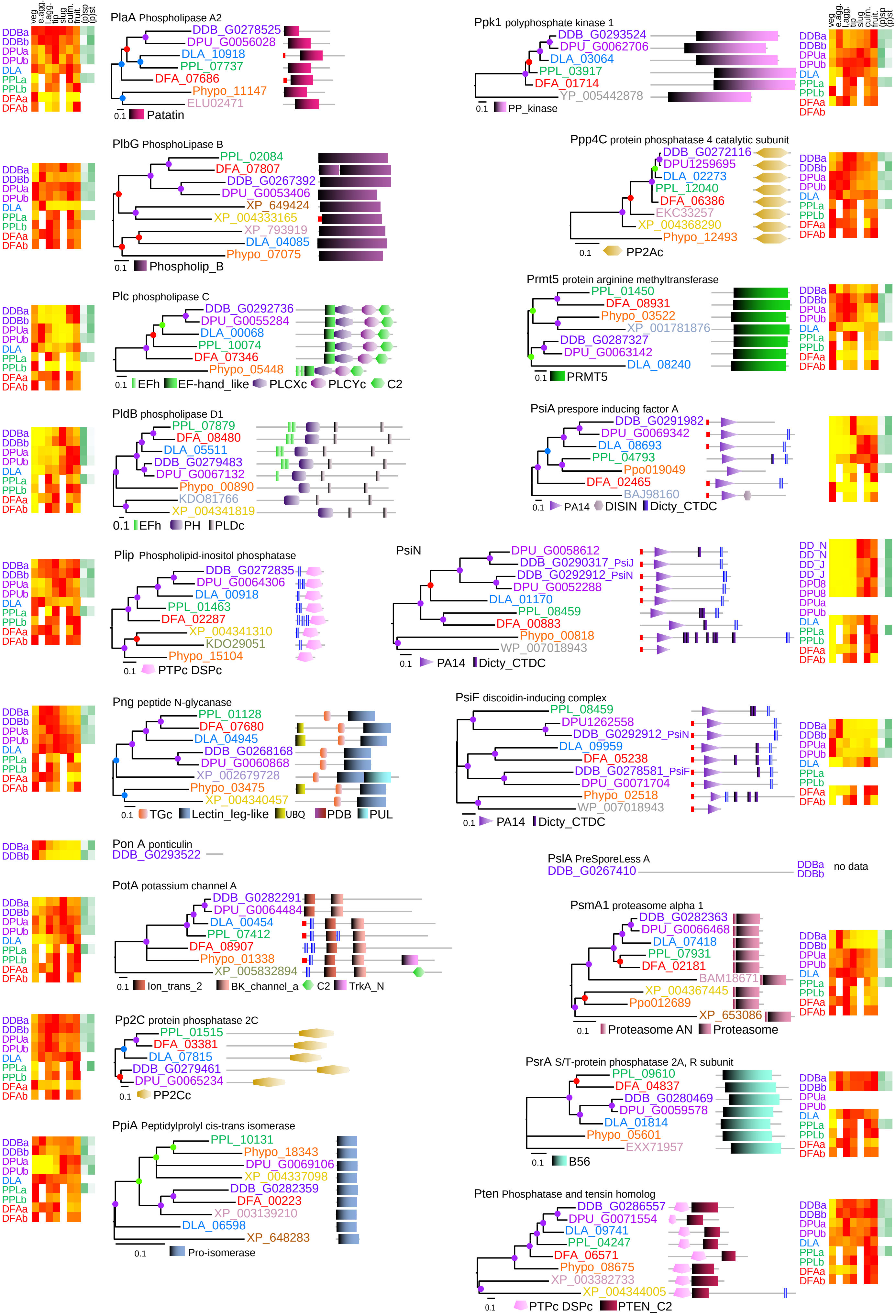
veg
e.agg.
l.agg.
tip
slug
culm.
fruit.
(p)sp
(p)st
PlaA Phospholipase A2
DDB_G0278525
DPU_G0056028
DLA_10918
PPL_07737
DFA_07686
Phypo_11147
ELU02471
0.1 Patatin
Ppk1 polyphosphate kinase 1
DDB_G0293524
DPU_G0062706
DLA_03064
PPL_03917
DFA_01714
YP_005442878
0.1 PP_kinase
PlbG PhosphoLipase B
PPL_02084
DFA_07807
DDB_G0267392
DPU_G0053406
XP_649424
XP_004333165
XP_793919
DLA_04085
Phypo_07075
0.1 Phospholip_B
Ppp4C protein phosphatase 4 catalytic subunit
DDB_G0272116
DPU1259695
DLA_02273
PPL_12040
DFA_06386
EKC33257
XP_004368290
Phypo_12493
0.1 PP2Ac
Prmt5 protein arginine methyltransferase
PPL_01450
DFA_08931
Phypo_03522
XP_001781876
DDB_G0287327
DPU_G0063142
DLA_08240
0.1 PRMT5
Plc phospholipase C
DDB_G0292736
DPU_G0055284
DLA_00068
PPL_10074
DFA_07346
Phypo_05448
0.1 EFh EF-hand_like PLCXc PLCYc C2
PldB phospholipase D1
PPL_07879
DFA_08480
DLA_05511
DDB_G0279483
DPU_G0067132
Phypo_00890
KDO81766
XP_004341819
0.1 EFh PH PLDc
PsiA prespore inducing factor A
DDB_G0291982
DPU_G0069342
DLA_08693
PPL_04793
Ppo019049
DFA_02465
BAJ98160
0.1 PA14 DISIN Dicty_CTDC
Plip Phospholipid-inositol phosphatase
DDB_G0272835
DPU_G0064306
DLA_00918
PPL_01463
DFA_02287
XP_004341310
KDO29051
Phypo_15104
0.1 PTPc DSPc
PsiN
DPU_G0058612
DDB_G0290317_PsiJ
DDB_G0292912_PsiN
DPU_G0052288
DLA_01170
PPL_08459
DFA_00883
Phypo_00818
WP_007018943
0.1 PA14 Dicty_CTDC
DD_N
DD_N
DD_J
DD_J
DPU8
DPU8
Png peptide N-glycanase
PPL_01128
DFA_07680
DLA_04945
DDB_G0268168
DPU_G0060868
XP_002679728
Phypo_03475
XP_004340457
0.1 TGc Lectin_leg-like UBQ PDB PUL
PsiF discoidin-inducing complex
PPL_08459
DPU1262558
DDB_G0292912_PsiN
DLA_09959
DFA_05238
DDB_G0278581_PsiF
DPU_G0071704
Phypo_02518
WP_007018943
0.1 PA14 Dicty_CTDC
Pon A ponticulin
DDB_G0293522
PslA PreSporeLess A
DDB_G0267410
no data
PotA potassium channel A
DDB_G0282291
DPU_G0064484
DLA_00454
PPL_07412
DFA_08907
Phypo_01338
XP_005832894
0.1 Ion_trans_2 BK_channel_a C2 TrkA_N
PsmA1 proteasome alpha 1
DDB_G0282363
DPU_G0066468
DLA_07418
PPL_07931
DFA_02181
BAM18671
XP_004367445
Ppo012689
XP_653086
0.1 Proteasome AN Proteasome
Pp2C protein phosphatase 2C
PPL_01515
DFA_03381
DLA_07815
DDB_G0279461
DPU_G0065234
0.1 PP2Cc
PsrA S/T-protein phosphatase 2A, R subunit
PPL_09610
DFA_04837
DDB_G0280469
DPU_G0059578
DLA_01814
Phypo_05601
EXX71957
0.1 B56
PpiA Peptidylprolyl cis-trans isomerase
PPL_10131
Phypo_18343
DPU_G0069106
XP_004337098
DDB_G0282359
DFA_00223
XP_003139210
DLA_06598
XP_648283
0.1 Pro-isomerase
Pten Phosphatase and tensin homolog
DDB_G0286557
DPU_G0071554
DLA_09741
PPL_04247
DFA_06571
Phypo_08675
XP_003382733
XP_004344005
0.1 PTPc DSPc PTEN_C2
DDBa
DDBb
DPUa
DPUb
DLA
PPLa
PPLb
DFAa
DFAb

Ptp-Rip

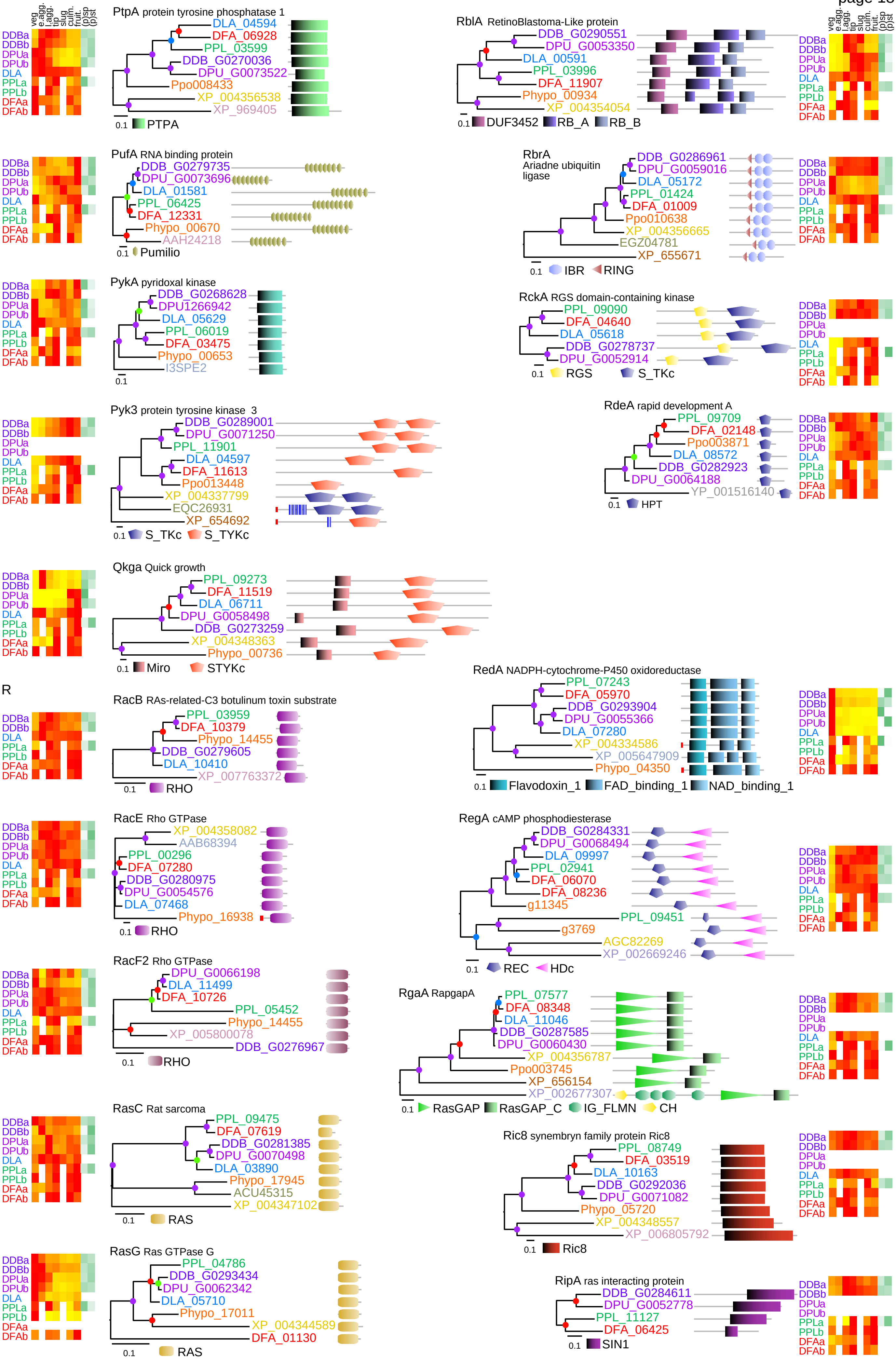
veg
e.agg.
l.agg.
tip
slug
culm.
fruit.
(p)sp
(p)st
DDBa
DDBb
DPUa
DPUb
DLA
PPLa
PPLb
DFAa
DFAb
PtpA protein tyrosine phosphatase 1
DLA_04594
DFA_06928
PPL_03599
DDB_G0270036
DPU_G0073522
Ppo008433
XP_004356538
XP_969405
0.1
PTPA
PufA RNA binding protein
DDB_G0279735
DPU_G0073696
DLA_01581
PPL_06425
DFA_12331
Phypo_00670
AAH24218
0.1
Pumilio
PykA pyridoxal kinase
DDB_G0268628
DPU1266942
DLA_05629
PPL_06019
DFA_03475
Phypo_00653
I3SPE2
0.1
Pyk3 protein tyrosine kinase 3
DDB_G0289001
DPU_G0071250
PPL_11901
DLA_04597
DFA_11613
Ppo013448
XP_004337799
EQC26931
XP_654692
0.1
S_TKc
S_TYKc
Qkga Quick growth
PPL_09273
DFA_11519
DLA_06711
DPU_G0058498
DDB_G0273259
XP_004348363
Phypo_00736
0.1
Miro
STYKc
R
RacB RAs-related-C3 botulinum toxin substrate
PPL_03959
DFA_10379
Phypo_14455
DDB_G0279605
DLA_10410
XP_007763372
0.1
RHO
RacE Rho GTPase
XP_004358082
AAB68394
PPL_00296
DFA_07280
DDB_G0280975
DPU_G0054576
DLA_07468
Phypo_16938
0.1
RHO
RacF2 Rho GTPase
DPU_G0066198
DLA_11499
DFA_10726
PPL_05452
Phypo_14455
XP_005800078
DDB_G0276967
0.1
RHO
RasC Rat sarcoma
PPL_09475
DFA_07619
DDB_G0281385
DPU_G0070498
DLA_03890
Phypo_17945
ACU45315
XP_004347102
0.1
RAS
RasG Ras GTPase G
PPL_04786
DDB_G0293434
DPU_G0062342
DLA_05710
Phypo_17011
XP_004344589
DFA_01130
0.1
RAS
RblA RetinoBlastoma-Like protein
DDB_G0290551
DPU_G0053350
DLA_00591
PPL_03996
DFA_11907
Phypo_00934
XP_004354054
0.1
DUF3452
RB_A
RB_B
RbrA
Ariadne ubiquitin
ligase
DDB_G0286961
DPU_G0059016
DLA_05172
PPL_01424
DFA_01009
Ppo010638
XP_004356665
EGZ04781
XP_655671
0.1
IBR
RING
RckA RGS domain-containing kinase
PPL_09090
DFA_04640
DLA_05618
DDB_G0278737
DPU_G0052914
0.1
RGS
S_TKc
RdeA rapid development A
PPL_09709
DFA_02148
Ppo003871
DLA_08572
DDB_G0282923
DPU_G0064188
YP_001516140
0.1
HPT
RedA NADPH-cytochrome-P450 oxidoreductase
PPL_07243
DFA_05970
DDB_G0293904
DPU_G0055366
DLA_07280
XP_004334586
XP_005647909
Phypo_04350
0.1
Flavodoxin_1
FAD_binding_1
NAD_binding_1
RegA cAMP phosphodiesterase
DDB_G0284331
DPU_G0068494
DLA_09997
PPL_02941
DFA_06070
DFA_08236
g11345
PPL_09451
g3769
AGC82269
XP_002669246
0.1
REC
HDc
RgaA RapgapA
PPL_07577
DFA_08348
DLA_11046
DDB_G0287585
DPU_G0060430
XP_004356787
Ppo003745
XP_656154
XP_002677307
0.1
RasGAP
RasGAP_C
IG_FLMN
CH
Ric8 synembryn family protein Ric8
PPL_08749
DFA_03519
DLA_10163
DDB_G0292036
DPU_G0071082
Phypo_05720
XP_004348557
XP_006805792
0.1
Ric8
RipA ras interacting protein
DDB_G0284611
DPU_G0052778
PPL_11127
DFA_06425
0.1
SIN1

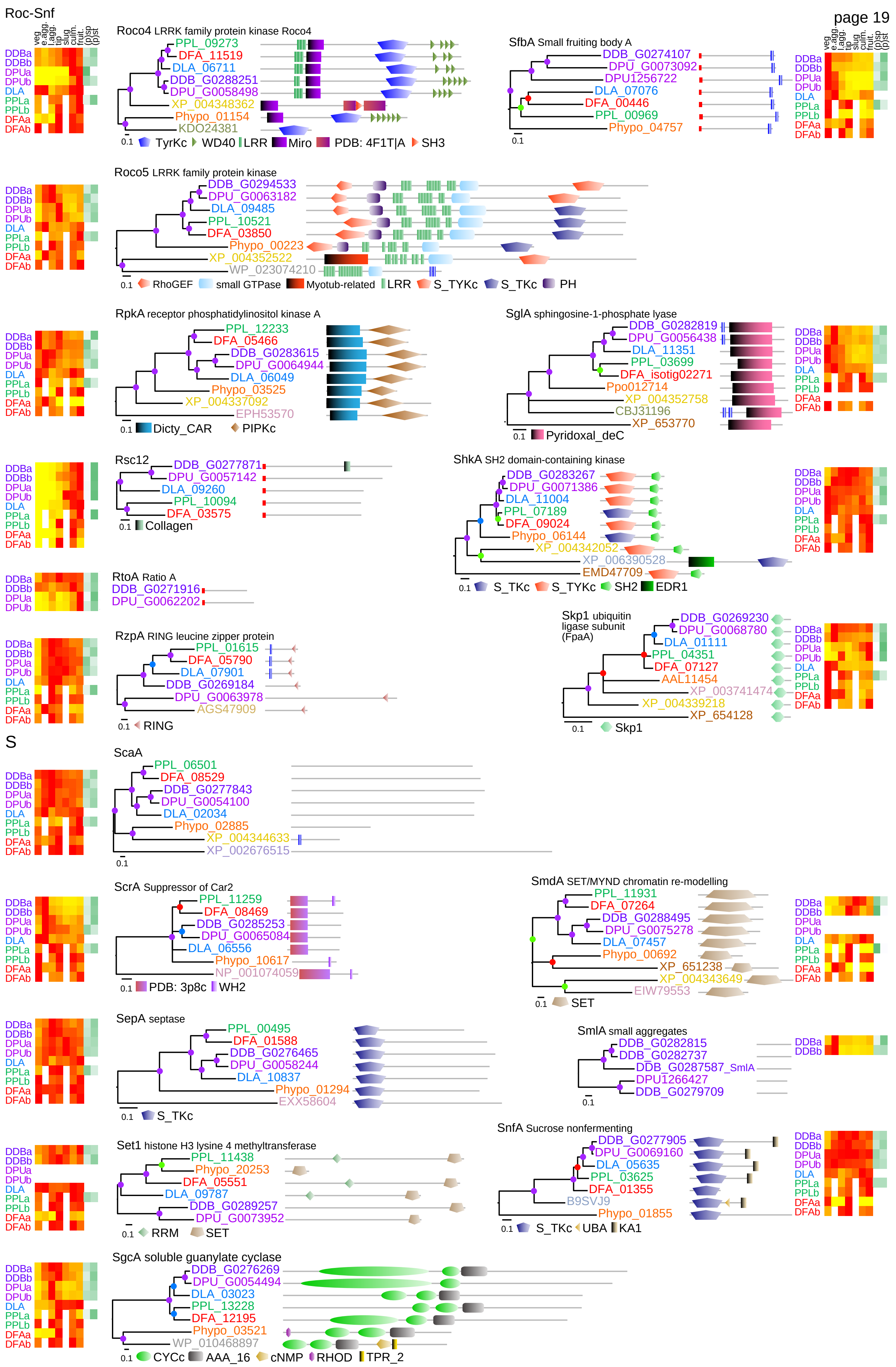
Roc-Snf
veg e.agg. l.agg. tip slug culm. fruit. (p)sp (p)st
DDBa DDBb DPUa DPUb DLA PPLa PPLb DFAa DFAb
Roco4 LRRK family protein kinase Roco4
PPL_09273
DFA_11519
DLA_06711
DDB_G0288251
DPU_G0058498
XP_004348362
Phypo_01154
KDO24381
0.1 TyrKc WD40 LRR Miro PDB: 4F1T|A SH3
SfbA Small fruiting body A
DDB_G0274107
DPU_G0073092
DPU1256722
DLA_07076
DFA_00446
PPL_00969
Phypo_04757
0.1
Roco5 LRRK family protein kinase
DDB_G0294533
DPU_G0063182
DLA_09485
PPL_10521
DFA_03850
Phypo_00223
XP_004352522
WP_023074210
0.1 RhoGEF small GTPase Myotub-related LRR S_TYKc S_TKc PH
RpkA receptor phosphatidylinositol kinase A
PPL_12233
DFA_05466
DDB_G0283615
DPU_G0064944
DLA_06049
Phypo_03525
XP_004337092
EPH53570
0.1 Dicty_CAR PIPKc
SglA sphingosine-1-phosphate lyase
DDB_G0282819
DPU_G0056438
DLA_11351
PPL_03699
DFA_isotig02271
Ppo012714
XP_004352758
CBJ31196
XP_653770
0.1 Pyridoxal_deC
Rsc12
DDB_G0277871
DPU_G0057142
DLA_09260
PPL_10094
DFA_03575
0.1 Collagen
ShkA SH2 domain-containing kinase
DDB_G0283267
DPU_G0071386
DLA_11004
PPL_07189
DFA_09024
Phypo_06144
XP_004342052
XP_006390528
EMD47709
0.1 S_TKc S_TYKc SH2 EDR1
RtoA Ratio A
DDB_G0271916
DPU_G0062202
Skp1 ubiquitin ligase subunit (FpaA)
DDB_G0269230
DPU_G0068780
DLA_01111
PPL_04351
DFA_07127
AAL11454
XP_003741474
XP_004339218
XP_654128
0.1 Skp1
RzpA RING leucine zipper protein
PPL_01615
DFA_05790
DLA_07901
DDB_G0269184
DPU_G0063978
AGS47909
0.1 RING
S
ScaA
PPL_06501
DFA_08529
DDB_G0277843
DPU_G0054100
DLA_02034
Phypo_02885
XP_004344633
XP_002676515
0.1
ScrA Suppressor of Car2
PPL_11259
DFA_08469
DDB_G0285253
DPU_G0065084
DLA_06556
Phypo_10617
NP_001074059
0.1 PDB: 3p8c WH2
SmdA SET/MYND chromatin re-modelling
PPL_11931
DFA_07264
DDB_G0288495
DPU_G0075278
DLA_07457
Phypo_00692
XP_651238
XP_004343649
EIW79553
0.1 SET
SepA septase
PPL_00495
DFA_01588
DDB_G0276465
DPU_G0058244
DLA_10837
Phypo_01294
EXX58604
0.1 S_TKc
SmlA small aggregates
DDB_G0282815
DDB_G0282737
DDB_G0287587_SmlA
DPU1266427
DDB_G0279709
0.1
Set1 histone H3 lysine 4 methyltransferase
PPL_11438
Phypo_20253
DFA_05551
DLA_09787
DDB_G0289257
DPU_G0073952
0.1 RRM SET
SnfA Sucrose nonfermenting
DDB_G0277905
DPU_G0069160
DLA_05635
PPL_03625
DFA_01355
B9SVJ9
Phypo_01855
0.1 S_TKc UBA KA1
SgcA soluble guanylate cyclase
DDB_G0276269
DPU_G0054494
DLA_03023
PPL_13228
DFA_12195
Phypo_03521
WP_010468897
0.1 CYCc AAA_16 cNMP RHOD TPR_2

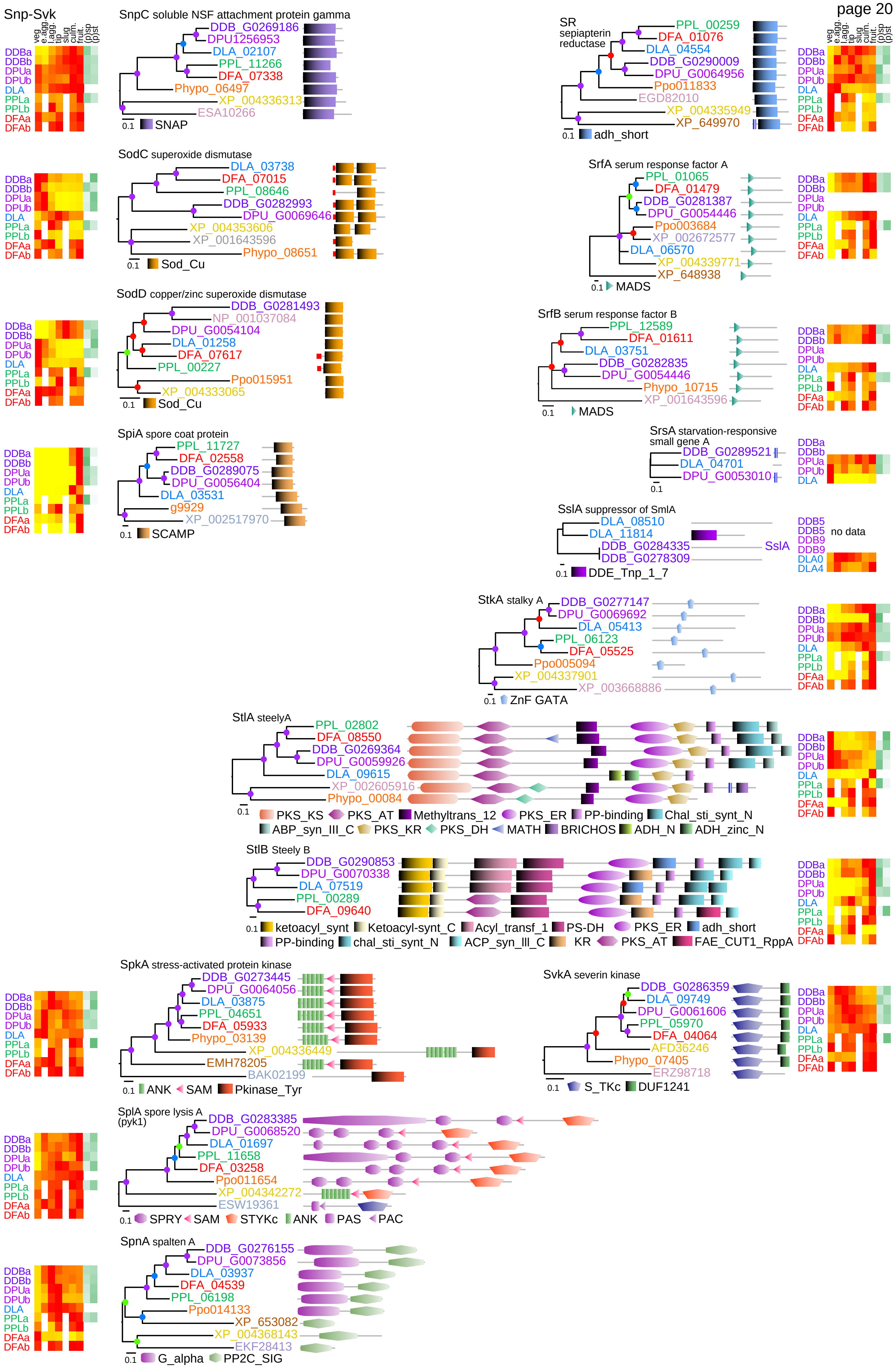

Snp-Svk
veg
e.agg.
l.agg.
tip
slug
culm.
fruit.
(p)sp
(p)st
DDBa
DDBb
DPUa
DPUb
DLA
PPLa
PPLb
DFAa
DFAb
SnpC soluble NSF attachment protein gamma
DDB_G0269186
DPU1256953
DLA_02107
PPL_11266
DFA_07338
Phypo_06497
XP_004336313
ESA10266
0.1 SNAP
SodC superoxide dismutase
DLA_03738
DFA_07015
PPL_08646
DDB_G0282993
DPU_G0069646
XP_004353606
XP_001643596
Phypo_08651
0.1 Sod_Cu
SodD copper/zinc superoxide dismutase
DDB_G0281493
NP_001037084
DPU_G0054104
DLA_01258
DFA_07617
PPL_00227
Ppo015951
XP_004333065
0.1 Sod_Cu
SpiA spore coat protein
PPL_11727
DFA_02558
DDB_G0289075
DPU_G0056404
DLA_03531
g9929
XP_002517970
0.1 SCAMP
SR sepiapterin reductase
PPL_00259
DFA_01076
DLA_04554
DDB_G0290009
DPU_G0064956
Ppo011833
EGD82010
XP_004335949
XP_649970
0.1 adh_short
SrfA serum response factor A
PPL_01065
DFA_01479
DDB_G0281387
DPU_G0054446
Ppo003684
XP_002672577
DLA_06570
XP_004339771
XP_648938
0.1 MADS
SrfB serum response factor B
PPL_12589
DFA_01611
DLA_03751
DDB_G0282835
DPU_G0054446
Phypo_10715
XP_001643596
0.1 MADS
SrsA starvation-responsive small gene A
DDB_G0289521
DLA_04701
DPU_G0053010
0.1
SslA suppressor of SmlA
DLA_08510
DLA_11814
DDB_G0284335
DDB_G0278309
SslA
0.1 DDE_Tnp_1_7
DDB5
DDB5
DDB9
DDB9
DLA0
DLA4
no data
StkA stalky A
DDB_G0277147
DPU_G0069692
DLA_05413
PPL_06123
DFA_05525
Ppo005094
XP_004337901
XP_003668886
0.1 ZnF GATA
StlA steelyA
PPL_02802
DFA_08550
DDB_G0269364
DPU_G0059926
DLA_09615
XP_002605916
Phypo_00084
0.1
PKS_KS PKS_AT Methyltrans_12 PKS_ER PP-binding Chal_sti_synt_N
ABP_syn_III_C PKS_KR PKS_DH MATH BRICHOS ADH_N ADH_zinc_N
StlB Steely B
DDB_G0290853
DPU_G0070338
DLA_07519
PPL_00289
DFA_09640
0.1 ketoacyl_synt Ketoacyl-synt_C Acyl_transf_1 PS-DH PKS_ER adh_short
PP-binding chal_sti_synt_N ACP_syn_III_C KR PKS_AT FAE_CUT1_RppA
SpkA stress-activated protein kinase
DDB_G0273445
DPU_G0064056
DLA_03875
PPL_04651
DFA_05933
Phypo_03139
XP_004336449
EMH78205
BAK02199
0.1 ANK SAM Pkinase_Tyr
SvkA severin kinase
DDB_G0286359
DLA_09749
DPU_G0061606
PPL_05970
DFA_04064
AFD36246
Phypo_07405
ERZ98718
0.1 S_TKc DUF1241
SplA spore lysis A (pyk1)
DDB_G0283385
DPU_G0068520
DLA_01697
PPL_11658
DFA_03258
Ppo011654
XP_004342272
ESW19361
0.1 SPRY SAM STYKc ANK PAS PAC
SpnA spalten A
DDB_G0276155
DPU_G0073856
DLA_03937
DFA_04539
PPL_06198
Ppo014133
XP_653082
XP_004368143
EKF28413
0.1 G_alpha PP2C_SIG

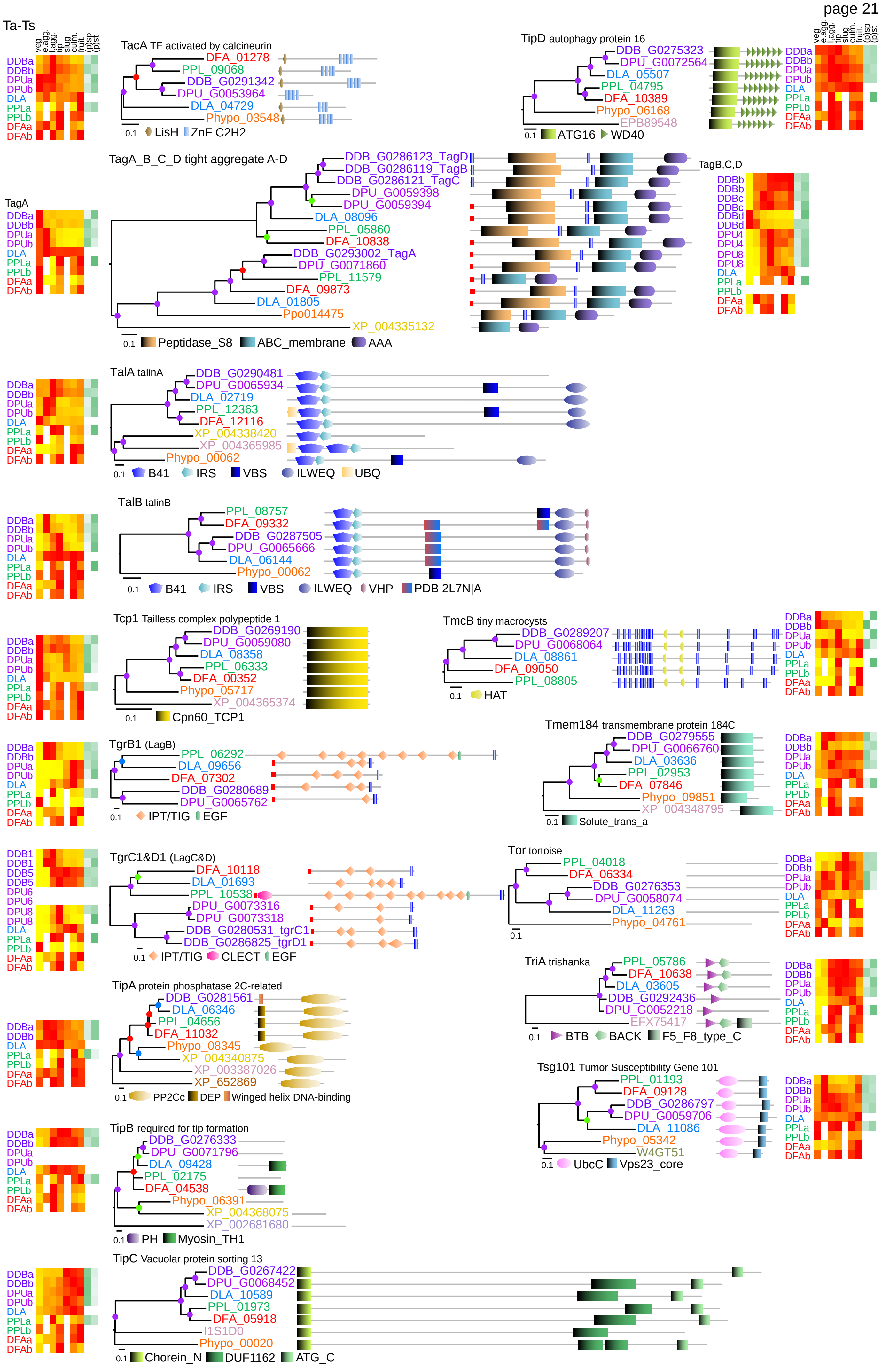
Ta-Ts
veg e.agg. l.agg. tip slug culm. fruit (p)sp (p)st
DDBa DDBb DPUa DPUb DLA PPLa PPLb DFAa DFAb
TacA TF activated by calcineurin
DFA_01278
PPL_09068
DDB_G0291342
DPU_G0053964
DLA_04729
Phypo_03548
0.1 LisH ZnF C2H2
TipD autophagy protein 16
DDB_G0275323
DPU_G0072564
DLA_05507
PPL_04795
DFA_10389
Phypo_06168
EPB89548
0.1 ATG16 WD40
TagA_B_C_D tight aggregate A-D
DDB_G0286123_TagD
DDB_G0286119_TagB
DDB_G0286121_TagC
DPU_G0059398
DPU_G0059394
DLA_08096
PPL_05860
DFA_10838
DDB_G0293002_TagA
DPU_G0071860
PPL_11579
DFA_09873
DLA_01805
Ppo014475
XP_004335132
0.1 Peptidase_S8 ABC_membrane AAA
TagA
TagB,C,D
DDBb DDBb DDBc DDBc DDBd DDBd DPU4 DPU4 DPU8 DPU8 DLA PPLa PPLb DFAa DFAb
TalA talinA
DDB_G0290481
DPU_G0065934
DLA_02719
PPL_12363
DFA_12116
XP_004338420
XP_004365985
Phypo_00062
0.1 B41 IRS VBS ILWEQ UBQ
TalB talinB
PPL_08757
DFA_09332
DDB_G0287505
DPU_G0065666
DLA_06144
Phypo_00062
0.1 B41 IRS VBS ILWEQ VHP PDB 2L7N|A
Tcp1 Tailless complex polypeptide 1
DDB_G0269190
DPU_G0059080
DLA_08358
PPL_06333
DFA_00352
Phypo_05717
XP_004365374
0.1 Cpn60_TCP1
TmcB tiny macrocysts
DDB_G0289207
DPU_G0068064
DLA_08861
DFA_09050
PPL_08805
0.1 HAT
TgrB1 (LagB)
PPL_06292
DLA_09656
DFA_07302
DDB_G0280689
DPU_G0065762
0.1 IPT/TIG EGF
Tmem184 transmembrane protein 184C
DDB_G0279555
DPU_G0066760
DLA_03636
PPL_02953
DFA_07846
Phypo_09851
XP_004348795
0.1 Solute_trans_a
TgrC1&D1 (LagC&D)
DDB1 DDB1 DDB5 DDB5 DPU6 DPU6 DPU8 DPU8 DLA PPLa PPLb DFAa DFAb
DFA_10118
DLA_01693
PPL_10538
DPU_G0073316
DPU_G0073318
DDB_G0280531_tgrC1
DDB_G0286825_tgrD1
0.1 IPT/TIG CLECT EGF
Tor tortoise
PPL_04018
DFA_06334
DDB_G0276353
DPU_G0058074
DLA_11263
Phypo_04761
0.1
TriA trishanka
PPL_05786
DFA_10638
DLA_03605
DDB_G0292436
DPU_G0052218
EFX75417
0.1 BTB BACK F5_F8_type_C
TipA protein phosphatase 2C-related
DDB_G0281561
DLA_06346
PPL_04656
DFA_11032
Phypo_08345
XP_004340875
XP_003387026
XP_652869
0.1 PP2Cc DEP Winged helix DNA-binding
Tsg101 Tumor Susceptibility Gene 101
PPL_01193
DFA_09128
DDB_G0286797
DPU_G0059706
DLA_11086
Phypo_05342
W4GT51
0.1 UbcC Vps23_core
TipB required for tip formation
DDB_G0276333
DPU_G0071796
DLA_09428
PPL_02175
DFA_04538
Phypo_06391
XP_004368075
XP_002681680
0.1 PH Myosin_TH1
TipC Vacuolar protein sorting 13
DDB_G0267422
DPU_G0068452
DLA_10589
PPL_01973
DFA_05918
I1S1D0
Phypo_00020
0.1 Chorein_N DUF1162 ATG_C

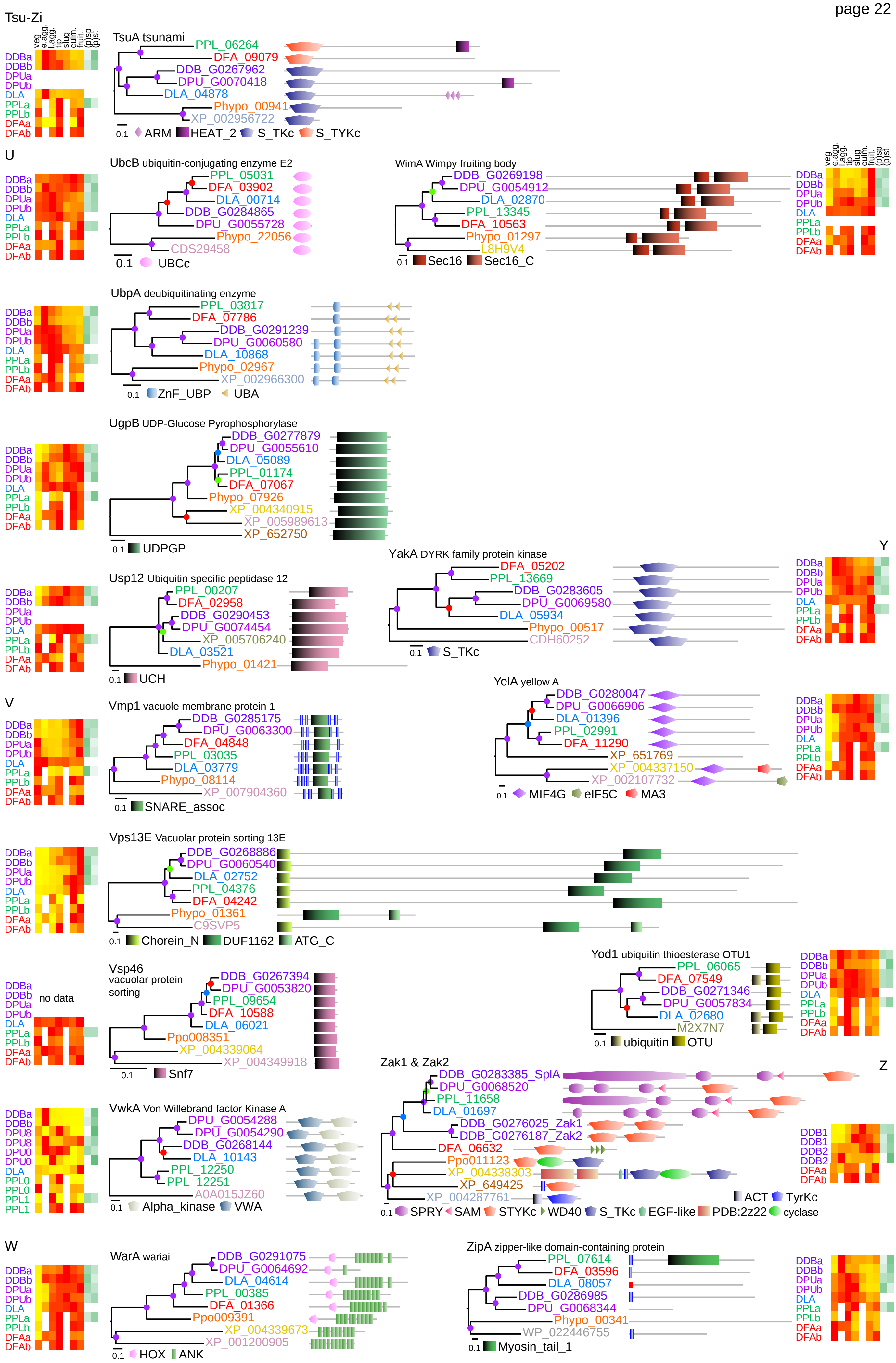
Tsu-Zi
veg. e.agg. l.agg. tip slug culm. fruit. (p)sp (p)st
DDBa DDBb DPUa DPUb DLA PPLa PPLb DFAa DFAb
TsuA tsunami
PPL_06264
DFA_09079
DDB_G0267962
DPU_G0070418
DLA_04878
Phypo_00941
XP_002956722
0.1 ARM HEAT_2 S_TKc S_TYKc
U
UbcB ubiquitin-conjugating enzyme E2
PPL_05031
DFA_03902
DLA_00714
DDB_G0284865
DPU_G0055728
Phypo_22056
CDS29458
0.1 UBCc
WimA Wimpy fruiting body
DDB_G0269198
DPU_G0054912
DLA_02870
PPL_13345
DFA_10563
Phypo_01297
L8H9V4
0.1 Sec16 Sec16_C
UbpA deubiquitinating enzyme
PPL_03817
DFA_07786
DDB_G0291239
DPU_G0060580
DLA_10868
Phypo_02967
XP_002966300
0.1 ZnF_UBP UBA
UgpB UDP-Glucose Pyrophosphorylase
DDB_G0277879
DPU_G0055610
DLA_05089
PPL_01174
DFA_07067
Phypo_07926
XP_004340915
XP_005989613
XP_652750
0.1 UDPGP
Y
YakA DYRK family protein kinase
DFA_05202
PPL_13669
DDB_G0283605
DPU_G0069580
DLA_05934
Phypo_00517
CDH60252
0.1 S_TKc
Usp12 Ubiquitin specific peptidase 12
PPL_00207
DFA_02958
DDB_G0290453
DPU_G0074454
XP_005706240
DLA_03521
Phypo_01421
0.1 UCH
V
Vmp1 vacuole membrane protein 1
DDB_G0285175
DPU_G0063300
DFA_04848
PPL_03035
DLA_03779
Phypo_08114
XP_007904360
0.1 SNARE_assoc
YelA yellow A
DDB_G0280047
DPU_G0066906
DLA_01396
PPL_02991
DFA_11290
XP_651769
XP_004337150
XP_002107732
0.1 MIF4G eIF5C MA3
Vps13E Vacuolar protein sorting 13E
DDB_G0268886
DPU_G0060540
DLA_02752
PPL_04376
DFA_04242
Phypo_01361
C9SVP5
0.1 Chorein_N DUF1162 ATG_C
Yod1 ubiquitin thioesterase OTU1
PPL_06065
DFA_07549
DDB_G0271346
DPU_G0057834
DLA_02680
M2X7N7
0.1 ubiquitin OTU
Vsp46 vacuolar protein sorting
no data
DDB_G0267394
DPU_G0053820
PPL_09654
DFA_10588
DLA_06021
Ppo008351
XP_004339064
XP_004349918
0.1 Snf7
Zak1 & Zak2
Z
DDB_G0283385_SplA
DPU_G0068520
PPL_11658
DLA_01697
DDB_G0276025_Zak1
DDB_G0276187_Zak2
DFA_06632
Ppo011123
XP_004338303
XP_649425
XP_004287761
0.1 SPRY SAM STYKc WD40 S_TKc EGF-like PDB:2z22 cyclase ACT TyrKc
VwkA Von Willebrand factor Kinase A
DPU_G0054288
DPU_G0054290
DDB_G0268144
DLA_10143
PPL_12250
PPL_12251
A0A015JZ60
0.1 Alpha_kinase VWA
DDBa DDBb DPU8 DPU8 DPU0 DPU0 DLA PPL0 PPL0 PPL1 PPL1
DDB1 DDB1 DDB2 DDB2 DFAa DFAb
W
WarA wariai
DDB_G0291075
DPU_G0064692
DLA_04614
PPL_00385
DFA_01366
Ppo009391
XP_004339673
XP_001200905
0.1 HOX ANK
ZipA zipper-like domain-containing protein
PPL_07614
DFA_03596
DLA_08057
DDB_G0286985
DPU_G0068344
Phypo_00341
WP_022446755
0.1 Myosin_tail_1
